# Esoteric beliefs and CAM impact SARS-CoV-2 immunization drivers, uptake and pediatric immunization views in Germany

**DOI:** 10.1038/s41541-024-00928-7

**Published:** 2024-08-03

**Authors:** Sebastian Jäckle, James K. Timmis

**Affiliations:** 1 Department of Political Science, University of Freiburg, Freiburg, 79085 Germany; 2 Athena Institute for Research on Innovation and Communication in Health and Life Sciences, Vrije Universiteit Amsterdam, Amsterdam, 1081 HV The Netherlands

**Keywords:** Risk factors, Viral infection

## Abstract

Recent studies demonstrate that sociopolitical attitudes partially explain variance in (SARS-CoV-2) vaccine hesitancy and uptake. Other attitudes, such as those towards esoteric beliefs, complementary and alternative medicine (CAM), and religion, have also been proposed. However, pertinent studies provide limited direction for public health efforts, as the impact of such attitudes has been tested in isolation or on different outcomes. Moreover, related associations between SARS-CoV-2 immunization drivers as well as views towards other modes of immunization (e.g., routine pediatric immunization), remain unclear. Based on a sample of ~7400 survey participants (Germany), where esoteric belief systems and CAM (Waldorf, homeopathy) are rather prevalent, and controlling for other sociological factors, we found that (i) individuals with positive attitudes towards Waldorf education and homeopathy are significantly less likely to have received a (further) dose of SARS-CoV-2 vaccine compared to those with positive views of mainstream medicine; (ii) for the former, immunization decisions are primarily driven by external pressures, and for the latter overwhelmingly by voluntary considerations; (iii) attitudes influencing adult SARS-CoV-2 vaccine uptake similarly influence views towards routine pediatric immunization. Our findings provide significant evidence informing a more nuanced design of public health and communication campaigns, and pertinent policies.

## Introduction

SARS-CoV-2 vaccines have prevented 14.4–19.8 million fatalities across the globe (between December 2020 and December 2021)^1^. Nevertheless, in most countries, SARS-CoV-2 vaccine uptake has remained suboptimal^2^. Studies have shown that this is, in no small part, due to a significant increase in the politicization of immunization^3–8^. Since political attitudes are shaped through social interactions and do not develop in isolation, several studies have explored the importance of socialization processes, and thus, factors describing the social environment of individuals as determinants of SARS-CoV-2 vaccine hesitancy (VacHes)^9–12^. These studies unequivocally demonstrate that sociopolitical attitudes are central determinants of SARS-CoV-2 VacHes.

While Germany is a particularly interesting case for the analysis of SARS-CoV-2 VacHes, due to significant regional variation in vaccine uptake, it is also a particularly urgent one, as German anti-vaccination groups are playing a central role in driving the currently unfolding, third (global) generation anti-immunization wave^13^. As recently demonstrated, support of the anti-system, right-wing party AfD (Alternative for Germany) is an especially strong predictor of lower immunization rates, particularly in East Germany^14^. However, political attitudes less robustly explain comparatively lower immunization rates in South Germany, which suggests that attitudes towards vaccination are also shaped by social interactions besides political ideology and partisanship^15^. Previous studies have shown that alternative and unorthodox worldviews, and conspiracy narratives, resonate with many vaccine hesitant individuals^16,17^. Studies exploring psychological and sociological determinants of VacHes, especially within the context of SARS-CoV-2, demonstrated that *spirituality and esoteric beliefs*, *attitudes towards complementary and alternative medicine (CAM), mainstream medicine*, and *religious association* can have a strong impact on individuals’ degree of resistance towards SARS-CoV-2 containment measures and views on immunization^18,19^. These considerations warrant the investigation of further potential drivers of SARS-CoV-2 vaccine uptake in Germany.

Several studies have shown that an anthroposophical worldview and pertinent lifestyle are significant risk factors for noncompliance with immunization programs, and outbreaks of infectious diseases^20,21^. The anthroposophical teachings of Rudolf Steiner are viewed particularly favorably in Germany^19^. Germany is also the country that has the highest number of Waldorf schools (also referred to as Steiner schools, as Waldorf education and pedagogy are rooted in Steiner’s anthroposophical teachings)—256 schools and 591 Kindergartens are officially recognized by German authorities^22–24^. The first Waldorf school was founded by Rudolf Steiner in 1919 as an on-site school for the children of employees of the Waldorf Astoria Zigarettenfabrik (cigarette manufacturer) in Stuttgart, Germany. Steiner’s aim was to “bring the Soul-Spirit into harmony with the Life-Body“^25^ and thereby counter-act the ‘social ills’ of society at the time^26^. Besides self-directed exploration, arts, physical movement and thematic (rather than subject-based) learning—spirituality is a central element of Waldorf education^27^. Steiner’s school of thought emphasizes an explicit and strong connection between pedagogical and medical ideas, i.e., anthroposophical medicine. According to the axioms of anthroposophical medicine, diseases are a consequence of an imbalance across the physical body, mind and spirit. Proponents believe that disease is, among other things, a consequence of bad karma and must be dealt with by a form of spiritual and physical healing and liberation, basically utilizing various supportive and behavioral interventions, and (medicinal) remedies of *inter alia* natural and ‘cosmic’ origin, such as planetary metals^28,29^. Although children who attend Waldorf schools and Kindergartens remain a minority in Germany (~1%), their numbers have risen steadily in the past years^30^. Recent studies have shown that children who attend Waldorf Kindergartens and schools are often un- or undervaccinated^31,32^, which has been associated with measles outbreaks in Germany and other countries^33–36^.

Also, CAM (primarily homeopathy) is viewed favorably by a substantial subset of the German population: 60% of Germans indicate that they have used homeopathic medicinal preparations at some point in time^37^. While homeopathic principles are also used in anthroposophical medicine, homeopathy is primarily concerned with physical (rather than spiritual) healing and has different axioms. Proponents of homeopathy believe that “a disease can be cured by a substance that produces similar symptoms in healthy people”—base active ingredients can be highly toxic—and, similarly, “the *lower* the dose of the medication, the *greater* its effectiveness” [italics in original]^38^, i.e. active ingredients are mostly untraceable. What unites proponents of homeopathy is their distrust of mainstream medicine and pertinent actors and institutions. Various studies have concluded that positive attitudes towards CAM are strongly associated with highly critical views of various mainstream medicine actors involved in organizing, developing and administering mainstream medicine interventions, i.e., *inter alia* vaccines^39,40^. In addition, a recent quantitative study among healthcare professionals (*N* = 2878) across four European countries found that “[…] CAM endorsement is associated with lower frequency of vaccine recommendation, lower self-vaccination rates, and being more open to patients delaying vaccination […]”, highlighting the importance of attitudes towards CAM of stakeholders that function as immunization accelerators and the potential significant impact on VacHes of patients^41^. Recently, in France, Spain and Great Britain, the reimbursement by statutory health insurance funds of homeopathic medicinal preparations was abolished —in Germany homeopathic medicinal preparations remain covered.

Regarding religious association, studies in the US and Brazil found that in particular followers of evangelical free churches view Covid-19 containment measures and immunization significantly less favorably than followers of other religious denominations^42–44^. In the US, scholars conclude that this is due to a particular form of Christian nationalism, which is formed through an amalgamation of religious beliefs and political ideologies^45^. To what extent similar associations can be found in Germany with its main denominations (Roman Catholicism and Protestantism) remains unclear.

While it has been established that, when examined in isolation or in different country-cohorts, (i) spirituality and esoteric beliefs, (ii) attitudes towards CAM and mainstream medicine, and (iii) religious association can be important determinants of (SARS-CoV-2) VacHes, it remains unclear to which degree these determinants act as confounders for vaccine uptake when collecting data on a distinct cohort and setting. Furthermore, there is substantial variation in the rationale driving SARS-CoV-2 immunization decisions^46^. Proper consideration of such motives is, therefore, particularly important for improving the likelihood that evidence-based/-informed policy-making and public health information campaigns provide intended incentives for immunization.

Lastly, studies have explored the impact of the SARS-CoV-2 pandemic more generally on parents’ degree of VacHes towards routine or SARS-CoV-2 pediatric immunization^47–50^. For instance, a US study found significant differences in attitudes towards pediatric vaccines for measles, mumps and rubella (MMR) and SARS-CoV-2 vaccines. While 72% of all adults believe that the former provides significant health benefits, only 45% think so regarding the latter. This is also partially reflected in the *de facto* adult SARS-CoV-2 immunization status: 74% of those adults who have not received a SARS-CoV-2 vaccine believe the benefits of pediatric MMR vaccines outweigh the risks^51^. To what extent similar differences exist in Germany, i.e., whether adult SARS-CoV-2 immunization status is associated with similar views on routine pediatric immunization (MMR) has not been explored. However, such an analysis is warranted because, as alluded to above, there are, in part, fundamental differences in the impact of attitudes driving VacHes in Germany vis-à-vis the US.

This study is based on three analyses (see Table 1) and aimed to extend other studies by providing a more granular account of the multifaceted phenomenon of VacHes with a view to facilitating the design of improved VacHes measurement instruments and higher immunization rates. Analysis I tested to what extent (i) attitudes toward Waldorf education (as a proxy for general attitudes toward the anthroposophical teachings of Rudolf Steiner), (ii) views on homeopathic medicine (as a proxy for CAM), on one hand, and mainstream medicine, on the other, as well as (iii) religious denomination, have affected individual’s SARS-CoV-2 vaccine uptake/immunization status (number of doses) in Germany. Since previous research has shown that SARS-CoV-2 VacHes in Germany is associated with region, age and (to a lesser extent) gender^14,52^, we additionally explored whether these sociodemographic factors moderate potential associations between the attitudinal variables and vaccine uptake. Analysis II focused on querying, among those who had received at least one dose, if drivers of SARS-CoV-2 immunization (e.g., protecting self/others, peer pressure/mandates) vary systematically regarding the attitudinal factors tested in Analysis I. In Analysis III, we tested if the associations we found in Analysis I are similar regarding the MMR vaccine. We use MMR as a case because, on the one hand, its use in US studies^51,53^ provides a good comparison to the German setting, and, on the other hand, awareness of this routine pediatric vaccine is likely highest also among individuals who are not acutely faced with the MMR vaccine decision. For a detailed discussion of theoretical considerations underpinning our three analyses and the peculiarities of the German case, see Supplementary Note 1.

**Table 1:** Overview of our study design and analyses

	Analysis I	Analysis II	Analysis III
Research Questions	What are the effects of attitudes towards Waldorf education, homeopathy, mainstream medicine, and religious denomination on SARS-CoV-2 immunization status (number of doses)?	What are the main drivers of the decision to have at least one dose of SARS-CoV-2 vaccine?	Are attitudes towards routine pediatric immunization (MMR) similarly driven by the effects observed in Analysis I?
Assumptions	See Supplementary Note 1 and Supplementary Table 6.		
Dependent variable(s)	SARS-CoV-2 immunization status (number of vaccine doses)	Importance of SARS-CoV-2 immunization drivers: (i) voluntary considerations and (ii) external pressures	Attitudes towards pediatric immunization (MMR)
Independent variables (main concepts)	• Attitudes towards Waldorf education • Attitudes towards homeopathy and mainstream medicine • Religious denomination		
Controls	• Political/ideological attitudes • Big 5 personality traits and degree of solidarity • Sociodemographic factors (age/gender/state (*Bundesland*)/education/household income)		
Population	Entire sample (*n* = 7391; German online survey)	Subset of the sample who had received at least 1 dose of SARS-CoV-2 vaccine (*n* = 6657)	Entire sample (*n* = 7391; German online survey)

## Results

### Sample

**Fig. 1:**
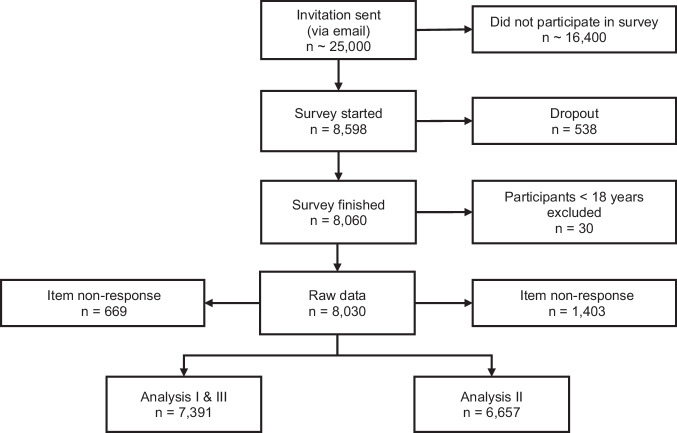
Survey participant flow diagram (initial sample to final samples). Online survey conducted in Germany between June 30th and July 17th, 2022. With 6.2% dropout was comparatively low. Item non-response was between 3.7% and 6.7% for each of the variables (see Supplementary Table 2) and the final samples for Analysis I & III and for Analysis II reduced via listwise deletion show no compositional differences to the raw data (see Supplementary Table 3).

Our statistical analysis is based on data from an online survey conducted in Germany between June 30th and July 17th, 2022. Of the 8598 individuals who started the survey, we used data provided by 7391 participants (no missing data) for Analysis I and III, and by 6657 (no missing data minus those who had not received at least one dose of vaccine) for Analysis II—see Fig. 1 for an overview, and Methods for further details on participants, missing data and representativeness.

### Descriptive statistics

On average, participants reported receiving 2.8 SARS-CoV-2 vaccine doses. Protecting self and others was the most important driver for immunization (mean = 0.85/0.78); peer pressure was the least important (mean = 0.23). Regarding routine pediatric immunization, the majority of participants viewed the MMR vaccine as highly useful (mean = 0.91). See Table 2 for an overview of the main variables.

**Table 2:** Descriptive statistics for the main variables tested in this study

	Scale	Raw data		Weighted data	
		Mean	Sd	Mean	Sd
**Dependent variables**					
*Analysis I (n* = 7391)					
# SARS-CoV-2 vaccine doses	0–4	2.77	0.97	2.81	0.96
*(crosscheck)*: Basic immunization (dummy variable: 2 or more doses)		*n* (%)		*n* (%)	
*0 or 1 dose*		700 (9.47)		658.49 (8.91)	
*2 or more doses*		6691 (90.53)		6732.51 (91.09)	
*Analysis II (n* = 6657)*					
Drivers of SARS-CoV-2 immunization decisions, self-perceived importance (0 = not at all important, 1 = very important)					
Protecting self	0–1	0.84	0.27	0.85	0.26
Protect others	0–1	0.78	0.29	0.78	0.29
Medical advice/recommendation of the Permanent Vaccination Commission (STIKO)	0–1	0.60	0.33	0.61	0.33
Participation in public events	0–1	0.57	0.33	0.58	0.33
Vocational mandates	0–1	0.37	0.37	0.38	0.37
Peer pressure	0–1	0.23	0.28	0.23	0.28
*Analysis III (n* = 7391)					
How useful do you consider routine pediatric immunization, e.g., against measles/mumps/rubella (MMR)? (0 = not at all meaningful, 1 = very meaningful)	0–1	0.91	0.20	0.91	0.20
**Independent variables (*n*** = 7391)					
Attitudes towards…					
Waldorf education (0 = negative, 1 = positive attitude)	0–1	0.40	0.25	0.40	0.25
Homeopathy (0 = negative, 1 = positive attitude)	0–1	0.39	0.31	0.42	0.30
Mainstream medicine (0 = negative, 1 = positive attitude)	0–1	0.77	0.20	0.77	0.20
Religious denomination		*n* (%)		*n* (%)	
No denomination		3808 (51.52)		3661.50 (49.54)	
Roman-Catholic		1464 (19.81)		1586.64 (21.47)	
Protestant		1252 (16.94)		1264.75 (17.11)	
Evangelical Free Church		651 (8.81)		695.03 (9.40)	
Orthodox Christian		21 (0.28)		20.54 (0.28)	
Jewish		11 (0.15)		9.38 (0.13)	
Muslim		36 (0.49)		34.00 (0.46)	
Other		148 (2.00)		119.16 (1.61)	
**Political & psychosocial controls (*n*** = 7391)					
Political ideology					
Left = 0—right = 1	0–1	0.41	0.20	0.44	0.20
Green/Alternative/Liberal (GAL) = 0—traditional/authoritarian/nationalist (TAN) = 1	0–1	0.45	0.24	0.49	0.24
Voting intention		*n* (%)		*n* (%)	
CDU/CSU (Christian democrats)		1076 (14.56)		1773.05 (23.99)	
SPD (Social democrats)		888 (12.01)		1348.04 (18.24)	
Greens		2505 (33.89)		1868.04 (25.27)	
FDP (Liberals)		631 (8.54)		575.06 (7.78)	
Left-Party (Socialist)		530 (7.17)		406.09 (5.49)	
AfD (Populist right wing)		545 (7.37)		787.00 (10.65)	
Others		1216 (16.45)		633.72 (8.57)	
Solidarity	0–1	0.66	0.17	0.65	0.17
Big five					
Agreeableness	0–1	0.53	0.19	0.53	0.19
Conscientiousness	0–1	0.67	0.20	0.69	0.20
Extraversion	0–1	0.52	0.25	0.54	0.24
Neuroticism	0–1	0.42	0.22	0.43	0.22
Openness	0–1	0.62	0.23	0.62	0.23

The weighted data uses a proportional iterative fitting (raking) procedure to adjust the data of the participants to the real distribution of the following factors in the German population: age group, gender, state, and voting intention. We apply these weights in all three analyses. For an overview of all variables, including sociodemographic controls, see Supplementary Table 3. *Analysis II uses data only on those 6657 participants who received at least one dose of SARS-CoV-2 vaccination.

### Analysis I—determinants of SARS-CoV-2 vaccine uptake

**Fig. 2:**
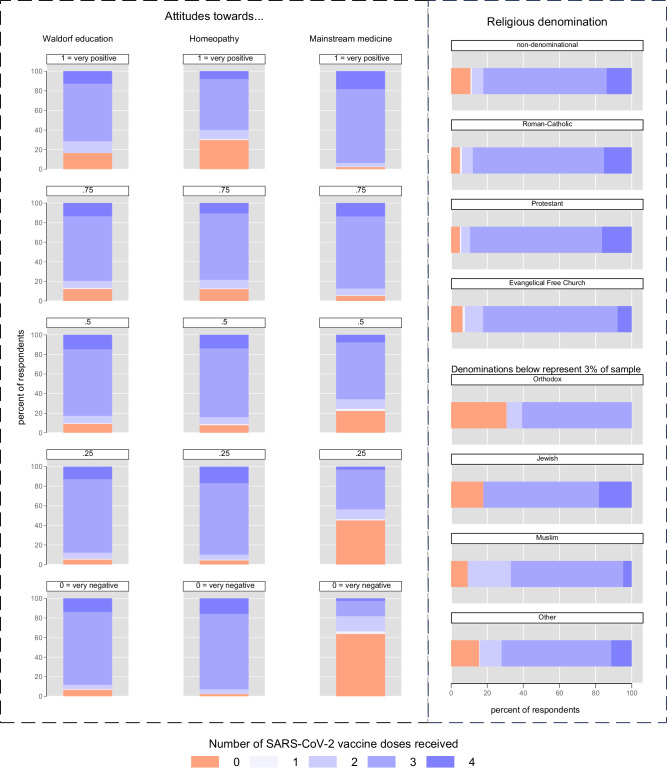
Vaccine uptake in Germany stratified by attitudes towards Waldorf education, homeopathy, mainstream medicine, and religious denomination (descriptive statistics)—analysis I. *N* = 7391. Data weighted by federal state, age group, gender und party affiliation to better represent the German population. NB: attitudes were queried separately, so a respondent could have indicated a negative, neutral or positive view towards both homeopathy and mainstream medicine concomitantly. This is, however, not only rather unlikely from a theoretical perspective—as both paradigms are, arguably, in part mutually exclusive—but our empirical data also show diverging patterns.

Figure 2 stratifies vaccine uptake (number of doses) by attitude and effect direction. Individuals who held favorable views towards Waldorf education and homeopathy were considerably less likely to have received a (further) dose of vaccine than those with less favorable views. By the same token, whereas 63% of those with very negative attitudes toward mainstream medicine were unvaccinated, 93.5% of those with very favorable views had received at least 3 doses. Substantially fewer differences exist regarding religious denominations. Among those self-classifying as non-denominational (49.5% of the sample), 10.7% were unvaccinated. Across the three largest German religious denominations—Roman-Catholics, Protestants, Free Evangelicals—only 4.9 to 6.4% were not immunized. We found comparatively high rates of unvaccinated individuals among those self-classifying as Muslim, Jewish, Orthodox Christian, or Other (<3% of the sample).

#### Models M1.1–M1.4: separate testing of associations between number of doses and main independent variables (see Table 3)

In M1.1, attitudes towards mainstream medicine remained the single most influential factor for vaccine uptake. Individuals who viewed mainstream medicine highly favorably, received on average an estimated 1.48 (*p* < 0.001) more doses of SARS-CoV-2 vaccine than those who held very negative views. In contrast, those who viewed homeopathy highly positively received on average 0.51 (*p* < 0.001) fewer doses than those who viewed homeopathy highly negatively. Attitudes towards Waldorf education were insignificant in M1.1. Regarding religious denominations, M1.2 shows that individuals self-classifying as Roman-Catholic or Protestant received on average 0.17 (*p* < 0.001) and 0.15 (*p* < 0.001) more vaccine doses than those self-classifying as non-denominational (reference category). The associations between other denominations and vaccine doses are statistically insignificant. M1.3 mirrors previous findings: partisanship was important for SARS-CoV-2 vaccine uptake^14^. As the most extreme example, supporters of the right-wing populist AfD received 1.37 (*p* < 0.001) fewer vaccine doses than the reference category Christian democrats. M1.4 shows that, on average, individuals with maximum solidarity scores received an estimated 1.32 (*p* < 0.001) additional doses relative to those with minimum solidarity scores. To a lesser degree, neuroticism also increased vaccine uptake. The other four personality traits did not play a role in M1.4.

**Table 3:** Impact on SARS-CoV-2 vaccine uptake (unstandardized b-coefficients from multiple linear regression): separate models by groups of variables (models M1.1–M1.4) and full model (M1.5)—Analysis I

Dependent variable:	M1.1	M1.2	M1.3	M1.4	M1.5
# SARS-CoV-2 vaccine doses (scale: 0–4)	Medicine and school attitudes	Religious denomination	Political factors	Psychological factors	Full model
Positive attitudes towards…					
Waldorf education	−0.0403				−0.134^**^
(scale: 0–1)	[−0.147, 0.0667]				[−0.232, −0.0355]
Homeopathy	−0.505^***^				−0.375^***^
(scale: 0–1)	[−0.602, −0.408]				[−0.461, −0.289]
Mainstream medicine	1.482^***^				1.034^***^
(scale: 0–1)	[1.316, 1.648]				[0.884, 1.185]
Religious denomination (Reference: no denomination)					
Roman-Catholic		0.174^***^			0.0732^*^
		[0.107, 0.242]			[0.0166, 0.130]
Protestant		0.153^***^			0.0304
		[0.0824, 0.223]			[−0.0290, 0.0897]
Evangelical Free Church		0.0628			0.0247
		[−0.0311, 0.157]			[−0.0562, 0.106]
Orthodox		−0.589			−0.399
		[−1.240, 0.0620]			[−0.809, 0.0105]
Jewish		−0.138			0.329
		[−1.232, 0.957]			[−0.547, 1.204]
Muslim		−0.179			−0.0677
		[−0.569, 0.211]			[−0.411, 0.276]
Other		−0.172			−0.0535
		[−0.430, 0.0857]			[−0.266, 0.159]
Left = 0/Right = 1			−0.127		−0.0378
(scale: 0–1)			[−0.345, 0.0903]		[−0.246,0.170]
GAL = 0/TAN = 1			0.137		−0.0194
(scale: 0–1)			[−0.0210, 0.294]		[−0.170, 0.131]
Voting intention (Reference: CDU/CSU: Christian democrats)					
SPD (Social democrats)			0.0697^*^		0.0509
			[0.00179, 0.138]		[−0.0160, 0.118]
Greens			0.0599		0.0397
			[−0.0164, 0.136]		[−0.0355, 0.115]
FDP (Liberals)			−0.297^***^		−0.232^***^
			[−0.396, −0.198]		[−0.329, −0.136]
Left-party (Socialists)			−0.223^**^		−0.160^*^
			[−0.365, −0.0799]		[−0.295, −0.0261]
AfD (Populist radical-right)			−1.365^***^		−1.158^***^
			[−1.498, −1.233]		[−1.287, −1.030]
Other (including Covid-19 protest party)			−0.926^***^		−0.717^***^
			[−1.070, −0.782]		[−0.847, −0.587]
Solidarity				1.318^***^	0.173^*^
(scale: 0–1)				[1.140, 1.496]	[0.00368, 0.343]
Agreeableness (Big 5)				−0.139	−0.149^*^
(scale: 0–1)				[−0.283, 0.00448]	[−0.272, −0.0255]
Conscientiousness (Big 5)				−0.106	0.0283
(scale: 0–1)				[−0.247, 0.0358]	[−0.0939, 0.150]
Extraversion (Big 5)				0.0360	0.0954
(scale: 0–1)				[−0.0770, 0.149]	[−0.00144, 0.192]
Neuroticism (Big 5)				0.296^***^	0.247^***^
(scale: 0–1)				[0.169, 0.422]	[0.139, 0.355]
Openness (Big 5)				−0.102	−0.0626
(scale: 0–1)				[−0.216, 0.0120]	[−0.162, 0.0372]
Constant	1.631^***^	2.409^***^	2.982^***^	1.730^***^	2.167^***^
	[1.347, 1.915]	[2.129, 2.690]	[2.715, 3.248]	[1.413, 2.047]	[1.836, 2.498]
Observations	7391	7391	7391	7391	7391
Adj. *R*^2^	0.216	0.0844	0.303	0.135	0.379

Data weighted by state, age group, gender and voting intention to better represent the German population. The models additionally control for state, gender, age group, education level, and household income (see Supplementary Table 7 for the effects of these sociodemographic controls). 95% confidence intervals in brackets. *p < 0.05, **p < 0.01, ***p < 0.001; *GAL* green/alternative/liberal, *TAN* traditional/authoritarian/nationalist.

#### M1.5: testing all variables in a full model (see Table 3)

First, six variables that had highly significant associations with vaccine uptake in separate models M1.1–M1.4 remained highly statistically significant in the full model M1.5 (*p* < 0.001), namely attitudes towards homeopathy and mainstream medicine, being a supporter of liberal FDP, far-right AfD and *Other* parties, and a higher degree on the Big 5 personality trait neuroticism. However, compared to M1.1 the strength of the correlations between vaccine uptake and mainstream medicine (positive association) and homeopathy (negative association) reduced by ~1/3 in M1.5. Secondly, the associations with Roman-Catholic and Protestant denominations as well as of solidarity decreased substantially compared to M1.2 and M1.4. Conversely, the associations with attitudes towards Waldorf education were considerably stronger and significant in M1.5 (*p* < 0.01). Additional stepwise control models showed that Waldorf education is significant if either political factors (left-right, GAL/TAN or voting intention) or the solidarity index are included in the model (Supplementary Table 8). Not controlling for these variables (M1.1) thus masks the association between Waldorf education and immunization status.

Regarding sociodemographic controls (not shown in Table 2) we found that vaccine uptake remained substantially lower in East German Saxony, and South German Bavaria and Baden-Wuerttemberg. Furthermore, 60+ is the only age bracket that had received significantly more doses of vaccine than the reference category of younger respondents (18–30). For income and educational level, we found no statistically significant differences in vaccine uptake.

#### Interaction models

Supplementary Figs. 1 and 2 show to what extent the associations between the main independent variables and vaccine uptake are moderated by age, gender and state. In brief: while gender had virtually no influence on these associations, we found differences regarding age and state. For the youngest age bracket (18–30 years), the positive association between vaccine uptake and negative attitudes towards Waldorf education was strongest. For the same age bracket, the associations between vaccine uptake and positive attitudes towards homeopathy (negative association) and positive attitudes towards mainstream medicine (positive association) were the weakest compared to older age brackets. Regarding regional variation, a positive view of mainstream medicine was associated with a higher number of doses in all 16 German states. We did not find this universal effect regarding attitudes towards Waldorf education and homeopathy. Bavaria was the only state in which both Waldorf education and homeopathy showed a significant negative association with vaccine uptake.

### Analysis II—drivers of SARS-CoV-2 immunization decisions

**Fig. 3:**
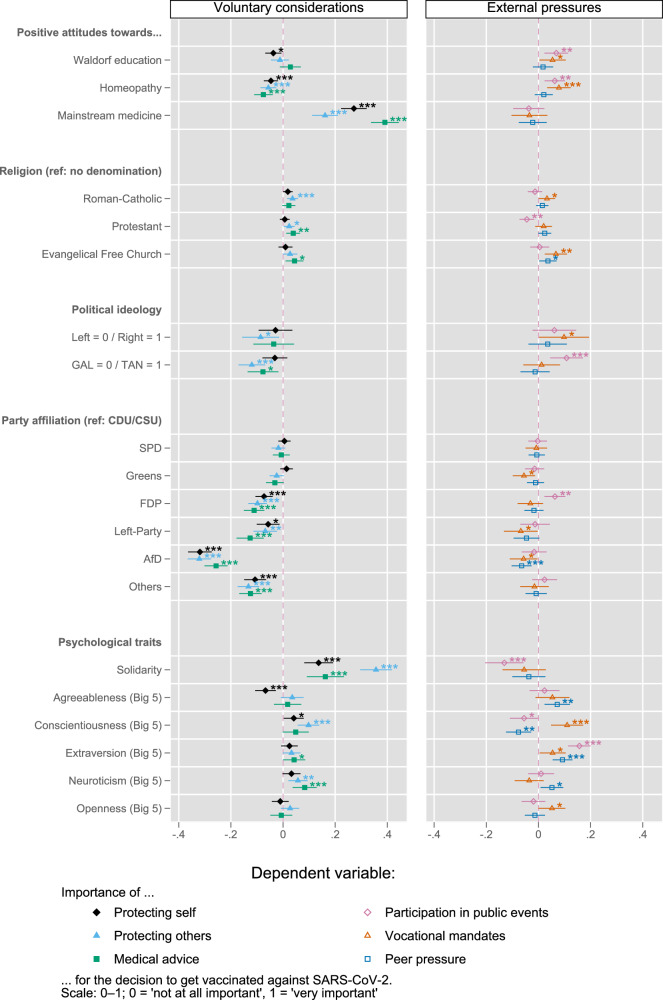
The self-perceived relative importance of six concrete reasons for SARS-CoV-2 immunization decisions—analysis II (unstandardized b-coefficients + 95% CI from multiple linear regression). *N* = 6657. Data weighted by state, age group, gender und party affiliation to better represent the German population. The models additionally control for state, gender, age group, education level, and household income (see Supplementary Table 11 for the full models). The effects of the following religious denominations are not presented due to a small number of cases resulting in very large CI: Jewish, Orthodox, Muslim, Other. *p < 0.05, **p < 0.01, ***p < 0.001; Lines represent 95% CIs. Reading example: a person who has a very positive view of mainstream medicine regards, all else being equal, medical advice to be 0.4 points more important (on a scale from 0 to 1) for their decision to get vaccinated against SARS-CoV-2 than a person who has a very negative view of mainstream medicine. All factors tested either measure on a scale from 0 to 1, or are dummy variables. Thus, the b-coefficients can be interpreted as a change in the dependent variable associated with a maximum change of an independent variable.

To determine the rationale for SARS-CoV-2 immunization, we focused on the subset of our sample who received at least one vaccine dose (*n* = 6657). For presentation purposes, we grouped the drivers into *voluntary considerations* and *external pressures*, see Fig. 3.

#### Attitudes towards Waldorf education, homeopathy, mainstream medicine, and religion

The more positive individuals viewed homeopathy or Waldorf education, the more important they regarded external pressures as drivers of their immunization decision. All else being equal and on a scale from 0 to 1, a person who held very positive views towards homeopathy (or Waldorf education), regarded *vocational mandates* 0.079 points, *p* < 0.001 (or 0.054 points, *p* < 0.05) more important for their decision to have a (further) SARS-CoV-2 vaccine dose compared with someone who held very negative views towards homeopathy (or Waldorf education). For the driver *participation in public events*, the model showed a 0.063, *p* < 0.01 (0.069, *p* < 0.01) higher point estimate for an individual who viewed homeopathy (or Waldorf education) very positively compared to those with very negative views. While more positive views towards Waldorf education were not significantly associated with voluntary considerations (*protecting self/others, medical advice*), those with positive views towards homeopathy unequivocally indicated that voluntary considerations were not important for their immunization decisions. Conversely, for individuals with very positive views towards mainstream medicine, voluntary considerations (such as *medical advice*/*self-protection*/*protecting others*) were significantly (*p* < 0.001) more important (0.391/0.272/0.161 points) for their immunization decisions than for those with very negative views on mainstream medicine. By the same token, we found no statistically significant association between attitudes towards mainstream medicine and importance of external pressures as drivers of immunization decisions. None of the attitudinal factors were significantly associated with the potential driver *peer pressure* (expectations of family and friends/acquaintances). Overall, the associations with religious denomination were marginal. However, for Catholics, *protecting others* tended to be more important whereas, for Protestants, *medical advice* tended to drive immunization decisions—*participation in public events* was irrelevant for Protestants. By tendency, vocational mandates were somewhat important for free evangelicals.

#### Sociopolitical attitudes

Regarding political ideology we found two highly significant associations (*p* < 0.001): the more traditional/authoritarian/nationalist (TAN) individuals were, the less important they rated *protection of others* and the more important they regarded the *possibility of participating in public events* for vaccine uptake. Compared to the reference category of Christian democrats (CDU/CSU), participants with party affiliation liberal FDP, socialist Left-Party, and particularly, populist radical-right AfD, deemed all voluntary considerations unimportant for their SARS-CoV-2 vaccination decision.

#### Psychosocial factors

The more solidary individuals were, the more important they regarded voluntary considerations for their immunization decision. An individual who was very solidary rated *protecting others/protecting self/medical advice* 0.357/0.137/0.163 points (*p* < 0.001) more important than someone who was completely non-solidary. For individuals with the highest solidarity score *participation in public events* was 0.131 points (*p* < 0.001) less important than for those with the lowest score. Personality traits affected immunization drivers differentially. Highly extraverted individuals rated *participation in public events*/*peer pressure* 0.157/0.093 points (*p* < 0.001) higher than highly introverted individuals. For individuals with very high scores on agreeableness, the importance of *self-protection* as a driver was 0.067 (*p* < 0.001) points lower than for those scoring very low on agreeableness. The more neurotic individuals were, the more *medical advice* (*b* = 0.083, *p* < 0.001) drove their immunization decisions. Individuals with a very high score on conscientiousness, rated both *protection of others* (voluntary consideration) and *vocational mandates* (external pressure) as significantly (*p* < 0.001) more important (0.098/0.11 points) than those with a very low score.

### Analysis III—effects on adult SARS-CoV-2 vaccine doses received vs. attitudes towards routine pediatric immunization (MMR)

**Fig. 4:**
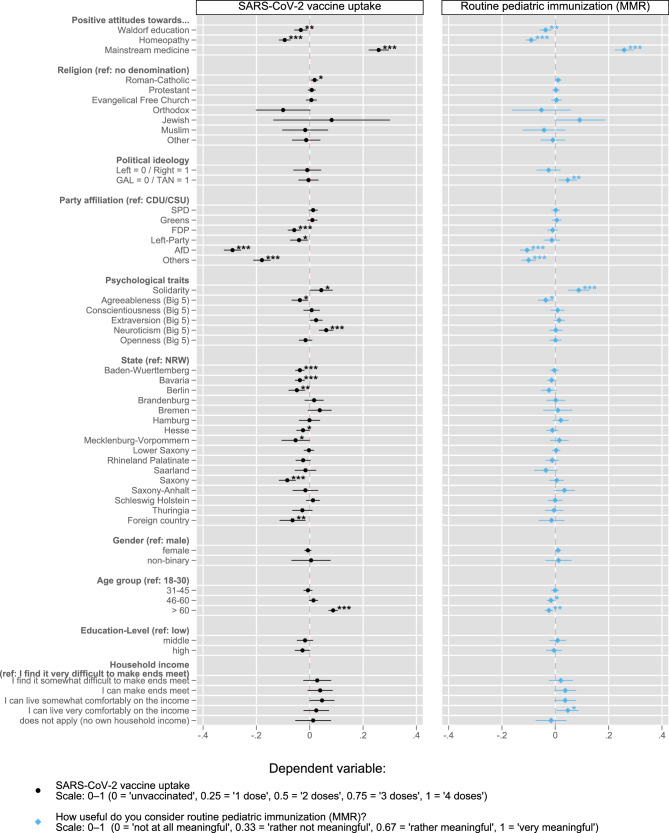
SARS-CoV-2 vaccine uptake and attitudes towards routine pediatric immunization (MMR)—Analysis III (unstandardized b-coefficients + 95% CI from multiple linear regression). *N* = 7391. Data weighted by state, age group, gender und party affiliation to better represent the German population. See Supplementary Table 13 for the full regression table. *p < 0.05, **p < 0.01, ***p < 0.001; Lines represent 95% CIs. Reading example: a person who has a very positive view of mainstream medicine regards, all else being equal, routine pediatric immunization (MMR) to be 0.26 points more meaningful (on a scale from 0 to 1) than a person who has a very negative view of mainstream medicine. All factors tested either measure on a scale from 0 to 1, or are dummy variables. Thus, the b-coefficients can be interpreted as a change in the dependent variable associated with a maximum change of an independent variable.

Figure 4 shows the highly similar pattern we found regarding the associations between our independent variables and both *SARS-CoV-2 vaccine doses received* and *attitudes towards routine pediatric immunization (MMR)*. This indicates that attitudes towards Waldorf education, homeopathy and mainstream medicine are solid predictors for both SARS-CoV-2 vaccine uptake and attitudes towards MMR vaccination. Religious denomination remained unimportant for both. However, regarding the controls some noteworthy differences exist. While the support of the AfD and *Other* parties was strongly negatively associated with vaccine uptake (*b* = −0.290/−0.179, both *p* < 0.001), the negative associations with MMR attitudes were considerably smaller (*b* = −0.106/−0.100, both *p* < 0.001). Regarding psychosocial factors, a higher degree on the solidarity index scale was, strikingly, the only association among our control variables leading to more positive attitudes towards the MMR vaccine (*b* = 0.088, *p* < 0.001). This association was twice as strong compared to SARS-CoV-2 vaccine uptake. Conversely, while positively associated with SARS-CoV-2 vaccine uptake (*b* = 0.062, *p* < 0.001), the personality trait neuroticism had no influence on MMR attitudes. What is also striking is that the association between older age (>60) and number of doses was positive (*b* = 0.88, *p* < 0.001) while the association direction was reversed for MMR attitudes (*b* = −0.024, *p* < 0.01).

## Discussion

Based on data from ~7400 respondents to a German survey from July 2022, we investigated (i) the impact of sociopolitical and psychosocial factors on adult SARS-CoV-2 vaccine uptake (number of doses), (ii) potential motive patterns, i.e., voluntary considerations and external pressures, driving immunization decisions, and (iii) whether attitudes towards routine pediatric immunization (MMR vaccine) are similarly shaped by factors tested for adult SARS-CoV-2 immunization status. The conclusions of our analysis are as follows: firstly, attitudes towards Waldorf education, homeopathy, and mainstream medicine are significantly associated with immunization status. Secondly, for individuals with positive views towards mainstream medicine, voluntary considerations (e.g., protecting self or others) primarily drive immunization decisions. Conversely, external pressures (e.g., vocational mandates) are primary drivers for those with positive views of homeopathy and, to a slightly lesser extent, Waldorf education. Thirdly, attitudes associated with SARS-CoV-2 immunization status are similarly associated with views on routine pediatric immunization (MMR). See Supplementary Table 6 for a comprehensive overview of our findings.

Scholars argue that, for certain social groups, anti-vaccine sentiments can act as social capital, i.e., can demonstrate dedication to and compliance with the (wider) community’s cause and thereby lead to social interaction rewards and cohesion^39,54^. Individuals in favor of CAM are usually organized in diverse social groups and united *inter alia* by their do-it-yourself approach to medicine, rejection of mainstream medicine (including vaccines), distrust of governmental and private (especially Big Pharma) healthcare entities, and rationalization of their views by cherry-picking (scientific) evidence of failures of mainstream medicine^39,40^. This also leads to the belief that parents who immunize their children are doing so based on inferior decision-making^40^. A 2015 study of a San Diego Waldorf school community found that, the longer families had been part of the school community, the stronger their anti-immunization views were. Strikingly, often parents were not familiar with the axioms of anthroposophy and, instead, focused on individualism and ‘good, protective parenting’. This was compounded by institutionally fostered skepticism towards governmental and private sector motives, in addition to “community rules favouring alternative perspectives and stigmatizing conventional ones”^31^. At least for this case, the indicated leverage of community-based social capital pressures implies closer alignment with CAM rationales than compliance with anthroposophical beliefs. By contrast, the knowledge of anthroposophical axioms and, in extension, anthroposophical medicine, plays a major role in vaccine hesitancy in European Waldorf school communities^31,36,39,55,56^. Supplementary Note 2 provides further information regarding vaccine exemption rates in anthroposophical communities in the US and European countries.

We found that the association between solidarity and vaccine uptake is strongly confounded. The strong and highly significant effect of solidarity in model M1.4, which tests psychological factors only, becomes weak and borderline significant in full model M1.5. A recent study investigating solidarity behaviors during the first and second lockdowns in Germany found that there are three clusters of individuals, namely ‘helpers’, ‘non-helpers’, and ‘help-receivers & helpers’. Non-helpers demonstrated (and received) the lowest degree of solidarity and tended to be young(er), indicated the lowest self-perceived risk and fear of infection with (a serious course of) SARS-CoV-2, displayed the riskiest behaviors and had the least trust in governmental institutions^57^. The characteristics of non-helpers likely have explanatory value for the confounding of solidarity. An individual who has a low-risk perception, and distrusts governmental advice and intervention, will likely not be vaccinated for voluntary reasons^58,59^. If these factors are compounded by positive attitudes towards individualism^39^, rejecting mass interventions^54^, and the belief that risk can be better managed through certain behaviors—e.g., ‘good, protective parenting’^54,60^—and/or that vaccines prevent important childhood developmental processes^39^, the likelihood of immunization could further decrease^61^.

Our comparison shows that the independent variables we tested have a largely identical impact on the likelihood of having *a (further) dose of SARS-CoV-2 vaccine,* i.e., Analysis I, and on attitudes towards *routine pediatric immunization (MMR)*, i.e., Analysis III. A high degree of solidarity and a marginally higher score on the TAN (traditional, authoritarian, nationalistic) spectrum were the only two factors more strongly associated with routine pediatric immunization (in favor of). While we found studies that investigated parents’ willingness to vaccinate their children against SARS-CoV-2^58,62^, or that used the pandemic itself as the independent variable to explore its impact on attitudes towards routine pediatric immunization^47–50^, we found none that directly compared adult SARS-CoV-2 vaccination status and attitudes towards routine pediatric immunization. As far as we are aware, we therefore provide the first, more complete overview demonstrating that, quite dissimilar to the findings of recent US studies^51,53^, adult SARS-CoV-2 vaccination status can be indicative of views on routine pediatric immunization (MMR), as we demonstrate in our German sample. A range of specific sociopolitical and psychosocial attitudes are robustly associated with both the willingness to receive the adult SARS-CoV-2 vaccine and views on routine pediatric immunization.

Our results have a range of implications for practice and policy. Recent studies found that, on the one hand, SARS-CoV-2 immunization status (number of doses) is the most important differential when stratifying narratives for public health campaigns, and, on the other, community benefits-based messaging (solidarity) is key to improving immunization rates in the unvaccinated^39,63^. Another study found that appeals based on personal benefits might be more persuasive for vaccine-hesitant individuals^64^. While we echo these findings in general, our results show that specific immunization drivers resonate differentially with certain attitudinal patterns—in particular, those highlighting individual social capital rewards. Hornsey & Fielding^65^ argue that individual, underlying (virtually unmodifiable) attitude roots can lead to the rejection of scientific consensus and advice on certain subjects if the latter is not compatible with existing worldviews, ideology, identity needs, etc. Moreover, countering evidence rejection by increasing the frequency of science communication likely has adverse effects, namely to push those who dismiss scientific evidence further towards their peer groups and strengthen their standpoints. Hornsey & Fielding suggest an approach they call *Jiu Jitsu Persuasion*—namely the utilization of (public health) narratives that reconcile potentially conflicting views by providing rationalizations for the harmonization of specific attitudes and motives of target demographics with societally desired outcomes^65^. For instance, our findings indicate that for vaccine-hesitant individuals in Germany who have received at least one vaccine dose and belong to mainstream medicine-sceptical groups, public health campaigns might be particularly effective if pertinent narratives are focused on positively perceived personal benefits enabled through immunization, e.g., participation in public events and the ensuing social rewards.

In any case, substantially more challenge study evidence is needed to a priori establish the (un)intended outcomes of different types of immunization messaging and policies, such as mandates, and understand if outcomes change over time. For example, it has been shown that in the later stages of immunization campaigns, mandates further decrease vaccine uptake by vaccine-hesitant individuals^66^. In addition, further experimental evidence is needed regarding attitudes towards, and the degree of societal support for, different nationally organized and provisioned immunization incentives (e.g., monetary rewards). Furthermore, vaccine hesitancy assessment instruments—such as the Vaccine Hesitancy Determinants Matrix (VHDMx)^67^—should not only be modified to include political attitudes, as we argued recently^14^, but also to incorporate items that briefly and accurately elicit attitudes towards alternative belief systems and those querying individually desirable social capital interactions and benefits.

Finally, while positive attitudes towards mainstream medicine are the single most important determinant driving vaccine uptake in our study, this stands in stark contrast with the effects of positive attitudes towards homeopathy (significantly decreasing uptake). The dichotomous nature of these results suggests that we have *de facto* documented a cleavage in German society that does not coincide with other well-established cleavages, such as left/right position or educated/non-educated, and is highly relevant for immunization decisions.

This study has a number of strengths that set it apart from previous research. First, it does not examine the hypothetical question of vaccine willingness, but rather actual immunization status. While several studies that tend to come from the medical/epidemiological field consider social background variables only peripherally, this study focuses on social interaction network effects while controlling for a large number of other potentially relevant explanatory factors. In parallel, the following limitations must be noted. First, the study is a survey conducted in Germany only. Direct transferability of the results to other countries is not readily possible. In view of the strong prevalence of both homeopathy and anthroposophy in Germany compared to other countries, the identified effects might be weaker elsewhere. However, good transferability of the results to Austria and Switzerland can be assumed, due to similar attitude patterns regarding Waldorf education and homeopathy^19^. Potentially, there might exist other forms of esoteric and CAM attitudes as a functional equivalent to homeopathy and anthroposophy in Germany, that might likewise condition attitudes toward vaccination (e.g., Traditional Chinese Medicine, see Supplementary Note 3). Because the sample was self-selected, the raw data are not truly representative of the German population. However, due to the sample size of ~7400 participants, it is possible to weight the data for all three analyses based on age, gender, state and voting intention, so that the results reflect the situation across Germany regarding relevant variables. Furthermore, as this is a cross-sectional analysis, potential changes over time cannot be detected. Although the association found between VacHes and CAM or esoteric attitudes proved to be robust even after controlling for various other variables, further research applying panel data and/or experimental approaches is needed to more adequately determine the direction of causality for these effects. Finally, as individuals self-classifying as Muslim, Jewish, Orthodox Christian or *Other* comprised <3% of our sample, solid inference regarding the associations between these religious denominations and our dependent variables is not possible.

## Methods

### Survey design

The survey was planned and designed in June 2022. Its primary goal was to provide empirical evidence regarding the effects of attitudes towards esoteric beliefs, CAM, mainstream medicine, and religious associations on SARS-CoV-2 vaccine uptake (Analysis I). Furthermore, in order to test if, and if so, to what extent, positive immunization decisions of individuals with specific attitudes towards esoteric beliefs, CAM, mainstream medicine, and religious association are motivated differentially, the survey included questions concerning two sets of potential immunization drivers, namely voluntary considerations and external pressures (Analysis II). Finally, in order to test whether the effects identified in Analysis I (on SARS-CoV-2 vaccine uptake) can also be observed for routine pediatric immunization, the survey also queried survey participants’ views on routine pediatric immunization MMR (Analysis III). After conceptualization, the questionnaire was programmed and pre-tested by student assistants and lay persons, which resulted in some minor adjustments. The field time of the survey was from June 30th to July 17th, 2022.

### Operationalization of variables

The statistical models were based exclusively on data queried in our survey—no secondary data was used. The exact formulation of the survey questions, the answer categories and the values used for the operationalization are shown in Supplementary Table 1 (in the German original and English translation). Since official guidelines varied over time in terms of how many doses from which manufacturer were required to be considered fully immunized, we decided to ask about the number of doses and not the immunization status as our dependent variable for Analysis I (see Supplementary Note 4). For Analysis II, those respondents who indicated that they had received at least one vaccine dose were asked how important six potential drivers were for their immunization decision (scale 0–1 with five levels from *completely unimportant* to *very important*). Analysis III assesses how useful the respondents consider routine pediatric immunization, e.g., against MMR. This question uses a scale 0–1 with four levels from *not at all meaningful* to *very meaningful*.

The independent variables measured attitudes towards Waldorf education, homeopathy and mainstream medicine (all on a scale of 0–1 with five levels from *very negative* to *very positive*) as well as religious denomination (eight categories). In order to facilitate comparison of the regression coefficients, all non-categorical independent and control variables were rescaled to a scale from 0 to 1 (see Supplementary Table 1). The unstandardized b-coefficients estimated in the linear regression models can, therefore, be interpreted readily as the associated change in the dependent variable when the independent variable is changed from its minimum to maximum value (*ceteris paribus*). To minimize social desirability bias and priming of our respondents as much as possible and thus reduce the introduction of systematic errors in data collection, we avoided the presentation of a stark contrast between educational systems and medical approaches, which otherwise would have immediately revealed our concrete interest in attitudes towards Waldorf education, homeopathy and mainstream medicine. The questions thus also provided alternative response options, i.e., other types of schools (state schools and private schools) and medical procedures/approaches (physiotherapy and acupuncture/acupressure). Correlations between our three main variables are relatively low (range: −0.162 to 0.342), indicating that they primarily measure distinct, mutually exclusive concepts and only, to a minor extent, the same underlying latent dimension. Exploratory factor analysis confirms this (see Supplementary Table 4).

We used scales and concepts that are common in attitudinal research as control variables. We applied the left/right- and GAL/TAN (Green, Alternative, Liberal/Traditional, Authoritarian, Nationalism)-scales to measure political ideology^68–70^. Within political survey research, the left-right self-ranking (left/right-scale) is the most commonly used instrument to measure an individual’s political ideology. It is utilized in many national and international surveys, e.g., the Eurobarometer, or the German Longitudinal Election Study (GLES) Long-term Panel. The left/right-scale is considered an instrument with a high degree of reliability (retests reliability) and validity (content and criterion validity), as tested by the German General Social Survey (ALLBUS)^71^. While the left/right-scale focuses primarily on economic differences (labor vs. capital), the GAL/TAN scale can detect ideological differences that are also rooted in cultural, social and morality domains. Regarding the sociopolitical dimensions of Covid-19, it has been shown that similar patterns apply to other identity-political issues (e.g., gender, climate crisis)—especially the belief in alternative, scientifically unsupported facts as adopted and propagated by certain social groups are central—which is why it is prudent to measure political ideological attitudes using the GAL/TAN scale in addition to the left-right scale. For both variables, we applied a graphical slider with an underlying 11-point Likert scale that showed only the two ending anchors (but no values or descriptors in between)^72^. Using this type of slider, an uneven and larger number of categories than in standard 5- or 7-point Likert scales is advantageous for rating political ideologies since it provides participants with an intuitive method of indicating their views on a one-dimensional political spectrum allowing for both the possibility of choosing a centre point but also a sufficiently granular self-positioning. For party affiliation, we used the *Sonntagsfrage*, i.e., Sunday question (“If federal elections were held next Sunday, which party would you vote for?”), as this is standard practice in Germany to query party affiliation. Participants could indicate one of the seven major parties (Christian democratic CDU and CSU, social democratic SPD, Greens, liberal FDP, right-wing populist AfD, socialist Left-Party) or *Other*.

Since psychosocial factors were not the main focus of our study and we wanted to balance the burden of including further sets of questions on our survey participants, we used short forms to measure personality traits and the degree of solidarity. We used the short form Big Five Inventory (BFI 10), a battery of 10 items which is considered highly efficient while still approximating the original Big Five Inventory (44 items^73^), with five item pairs each querying one of the following five personality traits: extraversion, neuroticism, conscientiousness, agreeableness and openness^74^. Each pair consists of a positively and a negatively formulated item. In the first step of analysis, the algebraic sign is reversed for the negatively coded items. In a second step the scores are calculated as the arithmetic mean of each item pair. Debates on SARS-CoV-2 immunization, as well as on MMR vaccination for children in Germany, have been partially framed as questions of solidarity. We, therefore, included a measure of solidarity which we assessed by means of a scale of four items (of which we calculated the mean value) on attitudes towards social and personal solidarity questions. These items were based on the German translation of the Social Responsibility Scale, consisting of a total of 22 items^75,76^. See below for a discussion of the reliability and validity of the solidarity and the Big Five scales applied in the survey.

The survey queried the following additional sociodemographic factors: age in years (for analysis, grouped into four age brackets: 18–30, 31–45, 46–60, 60+), gender (male, female, non-binary), place of residence (16 German states and foreign country), educational attainment by general school certificate (grouped into low, middle and high educational attainment according to the International Standard Classification of Education (ISCED) 2011) and household income. For the latter, we queried household income in relative terms. On one hand, answering questions about household income can be difficult and antagonizing for some individuals, which can increase the risk of the survey being abandoned or of item non-response. On the other, absolute numbers would introduce issues regarding comparability due to varying levels of cost of living in different German cities and regions. Instead, the survey queried a self-classification of individuals regarding how comfortably they felt they could live based on their household income. This question about the subjective evaluation of household income is easy to answer and allows good comparability of the participants even if they have different incomes in absolute terms. This question was adapted from the European Social Survey^77^.

### Participants, missing data, and representativeness

The survey was based on a long-time panel study at the University of Freiburg, Germany (*Politikpanel Deutschland*) that has surveyed several tens of thousands of people since 2017. The panel was initially recruited via diverse channels (participation in real-time response measurement experiments for political debates, local newspapers, and social media) and currently consists of a wide and heterogeneous group of ~25,000 individuals. For this work, all participants were informed via email about the possibility of participating in the survey. The duration of the survey was 15–20 minutes. In order to increase the response rate, we used an incentive: participants had the chance to win 20 Euro coupons (to be used at Amazon, a German Bookstore, an online organic supermarket, or donated to UNICEF). We also sent two reminders.

8598 individuals started the survey, providing informed consent at the very beginning of the online survey—8060 completed it (dropout 6.2%). Participants younger than 18 (*n* = 30) were excluded from the analysis. Item non-response was comparatively low (between 3.7 and 6.7% for the main dataset used in Analysis I and III, see Supplementary Table 2). Importantly, a comparison between the complete data and the datasets we used in the regression analyses (reduced via listwise deletion to 7391 cases for Analysis I and Analysis III and 6657 for Analysis II) shows virtually no compositional differences (Supplementary Table 3). Therefore, item non-response does not introduce substantial systematic bias into the dataset, which in turn means that a complete cases regression analysis (listwise deletion) is feasible and alternative imputation approaches are unnecessary. Supplementary Fig. 3 compares the raw data (already reduced to 7391 cases, including no missing values for all variables applied in Analysis I and III) with official statistics from the Federal Statistical Office of Germany on gender, age, education and place of residence by state (*Bundesland*). The data show that the proportion of male participants is indeed higher in our respondent sample than in the German population. The same is true for higher educated persons and participants from the city-state of Bremen and the southwestern state of Baden-Wuerttemberg. Regarding the age distribution, the sample lacks participants in the age bracket 75+. However, in direct comparison, the age distribution in our sample deviates less from the overall population than in other online surveys. For example, in the SoSciPanel^78^, another German, self-selecting online panel often used in the social sciences, 40.18% of the participants are between 21 and 34 years of age, whereas, according to the German Federal Statistical Office, this share is 20.38% in the general public (18+)^79^. In our sample, this age group represents 20.74%. Supplementary Fig. 3 also shows a comparison of the mean of our sample with respect to party affiliation to the mean of four representative opinion polls that were conducted in Germany between June 28th and July 6th, 2022 (Forschungsgruppe Wahlen, FORSA, Infratest Dimap, YouGov). These polls use exactly the same question (*Sunday Question*) as we do in our survey to query voting intention. In our sample, individuals with a party affiliation towards the Green party, liberal FDP and *Other* parties are overrepresented.

Overall, because of the different recruitment channels, the distribution of participants in terms of the aforementioned sociodemographic characteristics differs less markedly from the overall population than in many comparable self-selecting online surveys. Nevertheless, the raw data cannot be considered representative of the German population. We therefore weighted the raw data in order to more closely represent the German population.

### Weighting procedure

In order to achieve a more representative sample in terms of certain basic sociodemographic factors and political ideology, we applied an iterative proportional fitting (raking) weighting scheme^80^. Data of the participants were adjusted to the real distribution in the population using the marginal frequencies of age group (18–30, 31–45, 46–60, 60+), gender (m/f), state (*Bundesland*) and party affiliation (Sunday question). This weighting makes it possible to formulate generalizable statements about the entire population, similar to a random sample (see Supplementary Table 3 for a comparison of the raw data and the weighted data for all variables used in the study).

### Reliability and validity of survey instruments

Bierhoff demonstrated the reliability and validity of the 22-item German version of the Social Responsibility Scale, from which the measure of solidarity applied in this survey was adapted^76^. The four items used to construct the solidarity scale show high factor loadings in a principal components analysis (0.68–0.73) and a high Cronbach’s Alpha (*α* = 0.66), which—given the small number of only four items—both indicate a good internal consistency and thus reliability of the solidarity scale. The Big Five Inventory (BFI 10) applied in this survey has been tested extensively with regard to retest-reliability and validity by Rammstedt et al.^74^. According to their results, the BFI 10, although consisting of only two items per personality trait, can be regarded as a “reliable and valid recording of the Big Five. The BFI 10 allows a rough measurement of the individual personality structure of adult interviewees from the German-speaking general population”^74^. We validated by means of factor analysis—using the principle factor method—that the correct items were grouped together so that averaging their values (after reversing the negative formulated item) was meaningful. In our study, the internal consistency of four of the five scales was moderate to good, with Cronbach’s Alpha ranging between 0.41 and 0.73—again, keeping in mind the low number of only two items per trait. Only the measure for agreeableness shows a weak internal consistency (*α* = 0.16), yet this measure has also been found to have the lowest retest-reliability in earlier studies^74,81^. See Supplementary Table 5 for an overview of the internal consistency of the solidarity and the Big Five scales.

### Statistical analysis

#### Analysis I

To provide a first and descriptive overview, we stratified SARS-CoV-2 vaccine uptake (number of doses) by attitudes towards Waldorf education, homeopathy and mainstream medicine as well as by self-classified religious denomination. We presented these statistics as stacked bar plots (Fig. 2). Second, we ran multiple linear regression analysis to test whether the bivariate effects of our independent variables, as reported above, change, and if so how, when controlling for potential confounders. As further non-sociodemographic controls, we included political attitudes and psychosocial factors because, as alluded to above, these have been shown to substantially affect vaccine uptake.

Model building involved five steps (M1.1–M1.5 in Table 3): the first four models tested the effects of attitudes towards Waldorf education, homeopathy and mainstream medicine (M1.1), religion (M1.2), political attitudes (M1.3) and psychosocial factors (M1.4) on SARS-CoV-2 immunization status. M1.5 integrated all explanatory factors into a single model. All models additionally controlled for sociodemographic variables. Results are presented in a regression table (see Table 3 and Supplementary Table 7, including also the effects of the sociodemographic controls). In order to better understand why the coefficient of Waldorf education turned significant in the full model, we stepwise introduced our controls in additional models (Supplementary Table 8). Since the number of doses can also be interpreted as a count variable, we crosschecked model M1.5 with Poisson regression (Supplementary Fig. 4). The predicted numbers of doses from this Poisson model are for all values of the three main independent variables virtually identical to the ones from the multiple linear regression model. As a further crosscheck, the metric dependent variable was recoded into a dummy “basic immunization received” (0 = 0 or 1 dose, 1 = 2 or more doses, i.e., basic immunization). Binary logistic regressions replicating models M1.1–M1.5 show no significant differences (Supplementary Table 9). Additionally, models estimating interaction effects between attitudes towards Waldorf education, homeopathy and mainstream medicine, on the one hand, and age, gender and state, on the other, tested to what extent the effects of the attitudinal variables are universal or vary systematically with respect to certain sociodemographic characteristics. The results of these models are presented in Supplementary Table 10 and as plots of predicted values and plots of average marginal effects in Supplementary Fig. 1 and 2.

#### Analysis II

To explain the self-described importance of six potential drivers of SARS-CoV-2 immunization decisions among those participants of the survey who at least received one dose of vaccine (*n* = 6657), we applied linear regression. The drivers were: (i) *protecting self*, (ii) *protecting others*, (iii) *medical advice/recommendation by public health authorities* (e.g., German permanent vaccination commission STIKO), (iv) *participation in public events*, (v) *vocational mandates* and (vi) *peer pressure*. We grouped drivers into *voluntary considerations* (drivers i–iii) and *external pressures* (drivers iv–vi). In order to simplify interpretation and comparison of the effects, the results (Fig. 3) are presented in the form of a graph of unstandardized b-coefficients and 95% confidence intervals (Supplementary Table 11 for the full regression table). A crosscheck applying ordinal logistic regression yielded virtually the same results (Supplementary Table 12).

#### Analysis III

Our final analysis tested if, and if so, to which extent, the factors tested in, and pertinent effects observed in Analysis I (on SARS-CoV-2 vaccine uptake) also affect views towards routine pediatric immunization (MMR). We ran two linear regressions, one for the dependent variable vaccine uptake and one for attitudes towards routine pediatric immunization. In order to simplify comparison between the results of these two models, we rescaled vaccine uptake (0 to 4 doses) to the same scale we used to measure views towards MMR—range from 0 to 1. The results (unstandardized b-coefficients and 95% confidence intervals) are presented in Fig. 4. Supplementary Table 13 shows the results of the linear regression and of a crosscheck which applied ordinary logistic regression to the MMR data. Again, this type of regression analysis, interpreting the dependent variable as ordinal (and not as metric as in linear regression), did not produce any significantly different results.

#### Study registration

The three analyses were not pre-registered. This was neither required under local guidelines nor did it seem appropriate, given the, at least partially, exploratory nature of the research question.

#### Software used, replication code, and data

All statistical analyses were conducted using Stata 15. The underlying survey data and the analytical code are publicly available at Harvard Dataverse (10.7910/DVN/VRODRJ).

### Supplementary information

Supplementary Information

## Data Availability

The datasets generated and/or analyzed during the current study are available in the Harvard Dataverse repository, 10.7910/DVN/VRODRJ.

## Code availability

The underlying code for this study is available in Harvard Dataverse and can be accessed via this link 10.7910/DVN/VRODRJ.
