# Supplementary material for: Esoteric beliefs and CAM impact SARS-CoV-2 immunization drivers, uptake and pediatric immunization views in Germany
Source: NPJ Vaccines. 2024 Aug 3;9:137. doi: 10.1038/s41541-024-00928-7 (PMC11297982; doi:10.1038/s41541-024-00928-7)
Supplement: Supplementary file 1 — Supplementary Information [file 41541_2024_928_MOESM1_ESM.pdf]

Supplementary Information

Esoteric beliefs and CAM impact SARS-CoV-2 immunization drivers, uptake and pediatric immunization views in Germany

Supplementary Note 1: Theoretical background and assumptions guiding our analyses 3

Supplementary Note 2: Additional information on vaccine refusal and hesitancy in anthroposophic communities 9

Supplementary Note 3: Additional information on vaccine refusal and hesitancy Traditional Chinese Medicine 9

Supplementary Note 4: Additional information on the decision to use the number of SARS-CoV-2 vaccine doses as dependent variable in Analysis I 10

Supplementary Table 1: Survey Questions 11

Supplementary Table 2: Missing values – item non-response 15

Supplementary Table 3: Descriptive statistics – comparison of the original dataset and the final dataset used in the regression models, reduced via listwise deletion (unweighted and weighted data) 16

Supplementary Table 4: (a) Correlations between the main independent variables, and (b) exploratory factor analysis 18

Supplementary Table 5: Internal consistency of survey instruments 19

Supplementary Table 6: Overview of main assumptions and findings stratified by Analysis 21

Supplementary Table 7: Analysis I – Impact on SARS-CoV-2 vaccine uptake (unstandardized b-coefficients from multiple linear regression): separate models by groups of variables (models M1.1-M1.4) and full model (M1.5); complete regression table presenting also controls for state, gender, age group, education level and household income 22

Supplementary Table 8: Analysis I crosscheck – Impact on SARS-CoV-2 vaccine uptake (unstandardized b-coefficients from stepwise multiple linear regression) 26

Supplementary Table 9: Analysis I crosscheck – Binary logistic regression explaining whether a participant has received at least basic SARS-CoV-2 immunization (odds ratios) 28

Supplementary Table 10: Analysis I – interaction models (unstandardized b-coefficients from multiple linear regression) 32

Supplementary Table 11: Analysis II – Impact on the self-perceived relative importance of six concrete reasons for SARS-CoV-2 immunization decisions (unstandardized b-coefficients from multiple linear regression) 38

Supplementary Table 12: Analysis II crosscheck – Ordinal logistic regression explaining the level of self-perceived relative importance of six concrete reasons for SARS-CoV-2 immunization decisions (odds ratios) 42

Supplementary Table 13: Analysis III – Comparison between the impact on SARS-CoV-2 vaccine uptake and the impact on views towards routine pediatric immunization (MMR) (unstandardized b-coefficients from multiple linear regression + odds ratios from crosscheck with ordinal logistic regression) 46

Supplementary Figure 1: Estimated number of SARS-CoV-2 vaccine doses by attitudes towards Waldorf education, homeopathy and mainstream medicine over age group and gender (+ 95% CI) 49

Supplementary Figure 2: Average Marginal Effect of attitudes towards Waldorf schools, homeopathy and mainstream medicine on the number of SARS-CoV-2 vaccine doses by state (+ 95% CI)...50

Supplementary Figure 3: Distribution of main demographic variables in the sample compared to reality.....................52

Supplementary Figure 4: Predicted number of SARS-CoV-2 vaccine doses (+ 95% CI): Crosscheck multiple linear regression vs. Poisson regression 53

Literature 54

Supplementary Note 1: Theoretical background and assumptions guiding our analyses

The following sections explain in detail the theoretical basis of each of the three analyses, the main assumptions tested as well as the additional confounders controlled for in the statistical models. Parts of the explanations below are partial repetitions of information in the main article. This is because this section should be conclusive independently from the main article.

Analysis I – the impact of attitudes towards esoteric beliefs and CAM on SARS-CoV-2 vaccine uptake

Esoteric beliefs and spirituality

Recent studies demonstrate that spirituality and esoteric beliefs, as well as favourable attitudes towards homeopathy, can have a strong influence on resistance towards Covid-19 containment measures[1]. In Germany, such beliefs are chiefly rooted in the anthroposophical teachings of Rudolf Steiner, which are *inter alia* the basis of contemporary Waldorf schools, biodynamic farming (Demeter), and in turn anthroposophical medicine which, and with reference to mainstream medicine, is positioned as a holistic, individual-focused and complementary approach to health, disease and healing[2]. Followers of Steiner's teachings are particularly wide-spread in South Germany, Austria and Switzerland[1]. While not axiomatically identical, the ideas behind homeopathic medicine are also used in anthroposophical medicine and by those that might have similar worldviews.

Waldorf education

Waldorf, or Steiner, schools emphasise the creative, social, and emotional development of each individual pupil[3]. While catering to the abilities of each child/student self-directed exploration and thematic rather than subject-based learning, arts and physical movement, and spirituality are central pillars of Waldorf education. Eurythmy, an expressive movement art, developed by Rudolf Steiner plays an important role in Waldorf education. This form of modern dance is performed with the intention to stimulate the imagination and ideation capabilities of children, but it is also regarded as a relevant part of anthroposophical therapies[4]. Here the connection between pedagogical and medical ideas inherent in the anthroposophical worldview of Rudolf Steiner becomes apparent.

As of September 2022, there existed 1270 Waldorf schools and 1928 Waldorf Kindergartens worldwide, the majority of which were located in Germany: 256 schools and 591 Kindergartens[5]. Although children who attend Waldorf schools and Kindergartens remain a comparatively small minority regarding overall annual enrolment in Germany (about 1%), their numbers have nevertheless risen steadily in recent years[6]. These numbers indicate that perhaps even without exact knowledge about the principles of Steiner pedagogic, the Waldorf school system is seen by many Germans as a somewhat positive alternative to the state school system. Since the Waldorf pedagogical system is probably the most 'visible' component of the anthroposophical construct as compared to, for instance, biodynamic agriculture – many people do not know that biodynamic agriculture is rooted in the teachings of Rudolf Steiner – or anthroposophical medicine, it makes sense to use attitudes towards Waldorf schools as an indicator as to how favourably individuals view anthroposophical teachings in general. Furthermore, with respect to immunization, studies have shown that children who attend Waldorf Kindergartens and Schools are often un- or undervaccinated[7,8] which is discussed as the cause for the more frequent occurrence of measles outbreaks in German Waldorf schools compared to public schools[9–11].

Anthroposophical and homeopathic medicine

According to anthroposophical medicine, diseases (and health) are consequences of an (im)balance across the physical body, mind and spirit. Reincarnation, and pertinent good and bad deeds in previous lives play an important role in anthroposophical teachings and karma. Proponents reject the notion that a diseased individual, i.e. their body, is

considered ‘broken’ and must, therefore, be ‘fixed’ but that the “threefold anatomical nature, the nerve-sense pole, the rhythmic pole and the metabolic pole”[12] of man is out of balance. According to Kienle and colleagues, “anthroposophic medicine employs, in addition to conventional treatments, special medications and special therapeutic procedures, including eurythmy therapy, rhythmical massage, anthroposophic art therapy, and counseling. In addition, there are special anthroposophic nursing techniques”[13]. Although specific ‘active’ ingredients are selected according to different paradigms, the preparation of special medication-based remedies of anthroposophical medicine, in essence, is similar to that of homeopathic substances, or *vice versa*: the putative active ingredient is *potentised*, i.e. diluted (in stages) by a certain factor until, often, the initial ingredient is virtually undetectable.

The major difference between the healing substances used in anthroposophical medicine and in homeopathical medicine is that based on Samuel Hahnemanns principle similia similibus curentur (like cures like) the latter are harmful substances which are repeatedly diluted (‘potentised’) until they are finaly chemically indistinguishable from the diluent. Anthroposophical medicine instead assigns certain plant, animal and mineral substances to specific organs or organ disorders based on color and shape associations: e.g. yellow dandelion leaves, for example, serve as therapeutic agents for diseases of the liver, because these diseases are known to turn the skin and eyes yellow. So-called ‘cosmic materials’ which are said to be enriched with cosmic forces through certain procedures, are also important for anthroposophic medicines14. There exist different dilution factors, e.g. depending on the target indication and patient. Subsequently, a medium, such as a water, alcohol or lactose, is *dynamised*, i.e. mixed/infused with the diluted ingredient, and thence given as a treatment. While homeopathy is also used in anthroposophical medicine, homeopathic medicine is in principle and primarily concerned with physical rather than spiritual healing and has different axioms. Proponents of homeopathy believe in the notion that “a disease can be cured by a substance that produces similar symptoms in healthy people” – base active ingredients can be highly toxic – and, similarly, “the *lower* the dose of the medication, the *greater* its effectiveness” [italics in original][15], i.e. active ingredients are mostly untraceable. Also, disease classification and diagnostic approaches are different in homeopathic vis-à-vis anthroposophical medicine. Studies with solid research designs and reliable results demonstrating efficacy of anthroposophical or homeopathic substances are scarce. Existing studies frequently test multiple complementary interventions and endpoints – the *systems approach* of anthroposophical medicine – which, arguably, appears intuitive when viewing all clinical and extra-clinical elements of a therapeutic regimen as one holistic intervention. However, this also renders solid inference regarding the efficacy of specific elements – in particular of anthroposophical or homeopathic medications – a challenge, if not impossible. A systematic review study of Cochrane reviews finds for homeopathic medicine “no effects beyond placebo”[16]. A number of government level reviews (Switzerland, Great Britain, Australia) derived at largely similar results[17–19]. The European Academies’ Science Advisory Council also concludes in its statement on homeopathic products and practices that “there are no known diseases for which there is robust, reproducable evidence that homeopathy is effective beyond the placebo effect”[20].

Regarding immunization, vaccines protecting against infectious diseases and designed for broad application remain one-size fits all interventions. While Hornsey et al. found – among a Spanish sample of n=5.200 individuals (pre-Covid-19) – that distrust in mainstream medicine has a much stronger effect on general VacHes than positive attitudes towards complementary or alternative medicine (primarily homeopathy)[21], proponents of anthroposophical medicine axiomatically tend to be critical of national immunization schedules, because, as alluded to above, the focus of anthroposophical medicine and remedy is on specific factors pertinent to spirituality and the individual. In addition, proponents tend to believe that natural immunity, conferred by *de facto* infection, is superior to immunization[2]. There also exist homeopathic vaccinations, but evidence for their efficacy is lacking. Although portrayed as a complementary approach to mainstream medicine, proponents of anthroposophical and homeopathic medicine, and those of

mainstream medicine, tend to disagree on a range of axiomatic principles, such as what constitutes proper scientific enquiry and (convincing) evidence (e.g. in Rudolf Steiner's anthroposophical teachings, evidence is inter alia based on enquiry through spiritual science, as performed by meditation), the causes of diseases and symptoms, when medical interventions are necessary, approaches to treatment, etc. – distrust across paradigms is not unsubstantial. Similarly, and likely often closely linked to support of anthroposophical medicine and homeopathy, a low degree or lack of trust in governmental bodies and policy makers affiliated with health functions, public health institutes and pertinent advisory committees, and organisations and individuals delivering mainstream medical care, i.e. health systems, has been associated with a higher degree of vaccine hesitancy[22,23]. Ernst reports for example several measles outbreaks in the Netherlands, Germany, Great Britain and Austria that occurred within anthroposophical communities or Waldorf Kindergartens/Schools[24]. With respect to SARS-CoV-2 immunization, a German proponent of anthroposophy argues that immunization has serious consequences for the soul and the spirit after death:

> "we are not dealing here with a 'harmless prick', but rather with a powerful impulse to change the human body, so that it is hindered in its function as the vehicle for the soul-spiritual human being. This can also have a blocking effect on life after death. This means that the fundamental question concerning the future of humanity is at stake: Do we want to become a kind of 'machine-man' or do we want to remain beings of soul and spirit?"[25]

Although this quote is not representative of the entire anthroposophical following, and the publication it stems from has been coined "unscientific and manipulative" by the official anthroposophical society *Goetheanum*[26], the SARS-COV-2 vaccine-critical ideas from this book are widely propagated among adherents of the anthroposophical school of thought, particularly in German speaking countries.

Homeopathy and anthroposophical medicine are both considered forms of alternative medicine and are often confused. Since homeopathy is the much more widespread form of complementary/alternative medicine in Germany[27], it makes sense to understand attitudes toward it as a proxy in general for healing methods that position themselves as complements/alternatives to mainstream medicine.

Religious denomination

In the US, religious vaccine exemptions for school children are increasing, although on the absolute, less people identify as religious. Williams et al. recently explored religious belief or, more precisely, affiliation with a specific denomination as a further explanation for SARS-CoV-2 VacHes[28]. The correlation between VacHes and religious denomination as well as religiosity have been tested in numerous studies both in the context of the Covid-19 pandemic[29,30] as well as before with respect to other immunization campaigns[28,31]. Recent studies in the US and Brazil found that particularly followers of evangelical free churches view Covid-19 containment measures and, in turn, immunization significantly less favourably than other denominations[32,33]. Corcoran et al. argue that, in the US, this is due to a particular form of Christian nationalism – an amalgamation of religious beliefs and political ideologies – that "is one of the strongest predictors of COVID-19 vaccine hesitancy and is negatively associated with having received or planning to receive a COVID-19 vaccine"[34]. Given the fact that evangelical free churches represent, among other denominations, a small minority in Germany, it is not clear how these effects compare to, and interact, with political attitudes as determinants of VacHes in Germany. In 2020, 42% of the German population was non-denominational, about 26% were Catholic and 23% Protestant (with both of these groups significantly losing ground). Adherents of free evangelical churches represent less than 1% of the population, orthodox Christians about 2% and Muslims (incl. Alevism) about 6%, respectively[35].

Assumptions Analysis I

In line with the above explanations, we assume that individuals who have a positive attitude towards Waldorf schools and homeopathy have received fewer vaccine doses or have not been vaccinated, whereas individuals who have a high level of confidence in mainstream medicine should also have received significantly more vaccine doses. With regard to religion, given that in Germany, Evangelical Christians represent only a small minority, they should be less closely associated with right-wing nationalist ideas than in e.g., the US. Additionally, since neither of the two major religious communities (Catholic and Protestant Churches in Germany) have positioned themselves negatively towards immunization, we assume that religion has no strong influence on individuals' immunization status.

Analysis II – drivers of SARS-CoV-2 vaccine uptake

Very different reasons can drive the decisions of individuals to have a vaccine against SARS-CoV-2. On one hand, the fear of COVID-19 and thus self-protection might be the primary driver, but, on the other, the protection of third parties, especially vulnerable groups, may also be key. For others, the decision to have one (or more) shot(s) might have been less voluntary and self-driven and rather externally dictated, for example, if the employer required immunization, or if SARS-CoV-2 immunization was a necessary condition for participation in public events. These externally dictated drivers led to extensive debate in Germany. In the survey we therefore asked all participants who had received at least one dose of a SARS-CoV-2 vaccine to rate the importance of a total of six potential drivers for their own decision to get vaccinated on a five-point scale ranging from 1 "completely unimportant" to 5 "completely important". These drivers are 1) protecting self, 2) protecting others, 3) medical advice and recommendations by public health authorities (e.g., by the German Permanent Vaccination Commission (STIKO)), 4) unproblematic participation in public events (e.g., visiting a restaurant), 5) vocational mandates, and 6) peer pressure by family, friends and acquaintances.

Assumptions Analysis II

In light of the arguments discussed for Analysis I, our assumptions were that individuals who are more positive about anthroposophical ideas and homeopathic medicine are not only less likely to be vaccinated, but when they are vaccinated, they are more likely to mention external constraints/drivers and less likely to cite protection of their own or others' health as specific reasons for immunization. Medical advice is also less likely to be cited as a reason for SARS-CoV-2 immunization among this group. Conversely, participants with more positive views towards mainstream medicine should more often mention voluntary considerations as drivers. In particular we assumed that the more positive an individual views mainstream medicine, the more important they deem medical advice and recommendations by public health authorities for their decision to get vaccinated. With respect to religious denomination, it could be argued that members of Christian religious communities, for whom charity is often cited as a central principle, consider the protection of others from COVID-19 to be a more important driver than for people without religious affiliation.

Analysis III – comparing SARS-CoV-2 immunization status with attitudes towards routine pediatric immunization

Estimation of the extent to which our findings in Analysis I (selected determinants of adult SARS-CoV-2 immunization status) might be generalizable, i.e., indicative of hesitancy towards routine immunization, warranted comparison with immunization decisions that are based on motives besides self-protection. Immunizations for international travel and visits to regions that have other endemic infectious diseases are usually given for self-protection purposes, so do not fulfil this criterion and, in any case, most individuals are not frequently confronted with such necessities. By the same token, *routine pediatric immunization* in most cases serves to establish herd-immunity and thereby disrupt transmission routes while concomitantly protecting the individual child receiving the vaccine. However, *routine pediatric immunization*,

and in particular MMR (measles, mumps, and rubella) immunization, is a highly contentious issue for individuals and parents associated with certain subsets of populations[36]. An important propellant for the substantial increase in vaccine hesitancy (VacHes) was the 1998 publication in The Lancet medical journal of Andrew Wakefield's false claims stating that the recently developed MMR-vaccine caused autism. Regrettably, and although they have been extensively and repeatedly disproven, Wakefield's claims let the proverbial genie out of the bottle. Meanwhile, a quarter of a century later (Wakefield's paper was not retracted until 2010)[37], and fuelled by a range of actors, diverse anti-immunization movements exist and continue to cause pervasive uncertainty, doubt, confusion and distress among individuals, and groups thereof, who are confronted with, but *inter alia* unsure about, immunization – in particular parents considering pediatric immunization. Two recent studies explored the impact of the COVID-19 pandemic on parents' degree of VacHes towards routine childhood immunization. Based on a relatively small sample (n=252) of parents of children treated at two tertiary care centres in Los Angeles (USA), He et al. found evidence that parents' degree of VacHes regarding routine pediatric immunization (primarily due to increased risk perception) had risen during the first year of the pandemic – this, however, did not change their intention to routinely immunize their children[38]. Based on a much larger sample (n=4,562) of parents residing in Washington state and Colorado, Opel et al. found that, initially, VacHes towards routine pediatric immunization considerably decreased vis-à-vis before the onset of the pandemic but returned to pre-pandemic levels over the course of a few months – Opel et al. also used data collected during the first year of the pandemic[39]. A range of studies have explored similar relationships[40–42]. However, what is surprising is that – to the best of our knowledge – no studies collected the data nor ran proper analysis comparing parents' de facto SARS-CoV-2 immunization status, on one hand, with their views towards routine pediatric immunization, on the other. In consequence, it is unclear if (parents') attitudes towards adult SARS-CoV-2 immunization are indicative of (parents') attitudes towards routine pediatric immunization, or not. Importantly, as parents are likewise embedded within and receptive to social interaction/social capital-based systems, it is imperative to understand overall attitudes of a broader sample of individuals who likely, in turn, contribute to shaping the attitudes of others (*inter alia* parents) towards routine pediatric immunization. To explore a potential relationship between attitudes towards adult SARS-CoV-2 immunization and views towards routine pediatric immunization, we use the example of MMR vaccine. The MMR vaccine is probably *the* routine pediatric vaccine that has received the most sustained (and often highly controversial) media coverage and rigorous scientific scrutiny over the past decades. Therefore, awareness of this routine pediatric vaccine among individuals who are not acutely faced with the MMR vaccine decision is likely highest.

Assumptions Analysis III

As we have detailed in the introduction and shown in Analysis I and II, individuals that hold positive attitudes towards Waldorf schools and homeopathy (and likely also identify and socialize with like-minded peers) received fewer (or no) doses of vaccine and are mostly persuaded to have a vaccine based on external pressures. There exists some evidence, for example from Turkey, indicating an association between parent hesitancy towards childhood immunization and adult SARS-CoV-2 immunisation. However, this was based on the usual vaccine hesitancy measurement scales and does not take sociopolitical and psychosocial aspects into consideration[43]. Another study of parents in the US found that confidence in the benefit of vaccination was the most important predictor of child immunization behaviour[44]. Similarly, a key finding of a qualitative study in Canadian mothers was that negative experience or peer influences led to an increase in hesitancy[45]. However, there exists scarcity of evidence demonstrating to what extent the attitudes we show to have an impact on SARS-CoV-2 vaccination also have an impact on routine pediatric immunization. Based on the evidence found for other predictors as mentioned above, we assume that the impact of attitudes towards esoteric beliefs, CAM and mainstream medicine on adult *SARS-CoV-2 immunization* status is highly similar vis-à-vis overall attitudes towards *routine pediatric immunization* (as tested via the MMR vaccine).

Control variables

Political attitudes, personality traits, and socio-demographic characteristics

In order to draw as meaningful a picture as possible of the effects of esoteric spiritual worldviews, attitudes towards medical systems (homeopathy and mainstream medicine) and religious denomination on SARS-CoV-2 vaccine uptake (Analysis I), drivers of immunization (Analysis II) and attitudes towards routine pediatric immunization (Analysis III), our models additionally control for factors that have been shown to have explanatory value in previous research on VacHes, namely political ideology and party affiliation, psychological dispositions (personality traits and degree of solidarity) and sociodemographic characteristics.

In line with the results of previous studies[46–48], it is assumed that people with a more left-wing, green-alternative political attitude are more likely to have received more vaccine doses than people with a more right-wing conservative authoritarian attitude. Since certain parties in Germany, such as the right-wing populist Alternative for Germany (AfD) or the newly founded anti-COVID-19 parties (die Basis) and to a lesser extent also liberal Free Democrats (FDP) and socialist Left Party (Linke) vividly advocated against government-mandated SARS-CoV-2 containment measures and in particular against the immunization campaign[49], we assume that supporters of these parties have received fewer vaccine doses or have not been vaccinated compared to the supporters of the (other) mainstream parties.

Particularly over the past two decades, personality profiles, i.e. psychological dispositions, have attracted increasing interest among sociology and political science scholars as explanations for variation in *inter alia* political attitudes and behaviours. Personality profiles are often elicited by utilisation of data collection instruments based on the Big Five trait taxonomy, which classifies individuals' psychological dispositions towards the following personality traits: *Openness*, *Conscientiousness*, *Extraversion*, *Emotional Stability* (here *Neuroticism*), and *Agreeableness*. The five traits are defined by John et al.[50] and condensed by Gerber as follows:

> "Extraversion implies an *energetic approach* to the social and material world and includes traits such as sociability, activity, assertiveness, and positive emotionality... Agreeableness contrasts a *prosocial and communal orientation* toward others with antagonism and includes traits such as altruism, tender-mindedness, trust, and modesty... Conscientiousness describes *socially prescribed impulse* control that facilitates task- and goal-directed behavior, such as thinking before acting, delaying gratification, following norms and rules, and planning, organizing, and prioritizing tasks... [Emotional Stability describes even-temperedness and contrasts] with negative emotionality, such as feeling anxious, nervous, sad, and tense... Openness describes the breadth, depth, originality, and complexity of an individual's *mental and experiential life* (italics in original)."[51]

For instance, Gerber et al. report that individuals with higher scores (in relative terms) on personality traits *Openness* and, to a certain degree, *Emotional Stability*, and weighing the social benefits versus associated costs of participating in elections, tend to be more easily persuaded by political messaging/narrative that is based on social pressures and their intensions to vote increase accordingly[51]. By the same token, according to Huber et al. individuals with a higher score on personality trait *Extraversion* are more likely to – based on their disposition towards social interaction – participate in political debate and, in turn, collective political activities and action[52]. Measurement instruments based on the Big Five range from 120 items (statements) testing, and controlling for, various psychological dispositions towards one of the Big Five traits down to validated short forms that can include 10 items only (5 pairs of 2 items, each pair controlling for the psychological disposition towards one of the Big Five). Several recent studies have tested the association personality traits on SARS-COV-2 immunization decisions. However, results are mixed. For example, while Howard finds a negative effect of extraversion and positive effects of conscientiousness and neuroticism on VacHes[53],

Reagu et al. conclude that openness and neuroticism are negatively related to VacHes[54], while others find that only extraversion is significantly positively related with VacHes[55].

Finally, the following sociodemographic factors that have been applied in previous studies on SARS-COV-2 VacHes in Germany[48] will be used as controls: age, gender, federal state (Bundesland), household income and education level. In addition, these controls provide the means to test whether potential effects of esoteric beliefs and religious denomination are more or less universal among all Germans or whether they vary for example with age, gender or state (see Supplementary Figures 1 and 2).

Supplementary Table 6 gives an overview of the main assumptions for our three Analyses together with the major findings.

Supplementary Note 2: Additional information on vaccine refusal and hesitancy in anthroposophic communities

For the school year 2020-2021, kindergarten children at entry into public schools had an average immunization rate of 94% across the US, i.e. ≤6% exemptions[56]. Vaccine exemption rates at Waldorf schools across California – the state with the greatest number of (and publicly funded) Waldorf schools in the US – averaged at 45.1%[57]. Another study found that among a San Diego Waldorf school community, 51% of pupils were not, or not fully, immunized[7]. In a recent systematic literature review investigating the relationship between anthroposophical beliefs and vaccine willingness, Herzig van Wees et al. found that children attending various anthroposophic schools and kindergartens in Germany, the Netherlands, the USA, and Switzerland, have significantly lower immunization rates for poliomyelitis, diphteria, pertussis, haemophilus influenzae, hepatits, pneumococcal vaccine, meningococcus C, and, in particular, measles, mumps and rubella[58]. The review also includes a number of studies that focus on the factors influencing parent immunisation decision making within anthroposophic communities. Some parents reject immunizing their children in general because they are either concerned about vaccine ingredients and potential side effects or believe that natural immunity is superior to 'artificial immunity' as established by immunization. Other parents decide based on their perception of risk. For example, immunization against tetanus, on one hand, is significantly less strongly opposed within anthroposophical communities than other vaccination. Immunisation against measles, mumps and rubella, on the other, is particularly strongly opposed[58]. This is another reason why we used MMR vaccination as our dependent variable in Analysis III.

Supplementary Note 3: Additional information on vaccine refusal and hesitancy Traditional Chinese Medicine

Potentially, analogous negative effects on the vaccine uptake as the ones we found for attitudes towards Waldorf education and homeopathy for the German case could exist for other CAMs. One example might be traditional Chinese medicine TCM, which "differs in substance, methodology and philosophy to modern medicine"[59] and despite distinct differences exhibits an astonishing variety of similarities with homeopathy[60,61]. Recent evidence suggests that TCM in mainland China is primarily favored by younger (ages 25–35), highly educated and urban cohorts[62]. Qualitative evidence also points toward a connection between the TCM concept of *Ti Zhi* – a multifaceted construct covering physical, mental and cultural aspects – and SARS-CoV-2 vaccine refusal in younger Chinese adults (ages 18-40). Most of those who used *Ti Zhi* as a reason for vaccine refusal stated that they believe Chinese vaccines are safe but that their *Ti Zhi* is not

suitable for immunization/specific vaccines. Strikingly, most of this group were not clear on what the concept of *Ti Zhi* actually means[63].

Supplementary Note 4: Additional information on the decision to use the number of SARS-CoV-2 vaccine doses as dependent variable in Analysis I

It might be confusing for respondents to self-classify into immunization status categories, such as *fully immunized* (for a certain period) or *'boostered'*, based on the number and type(s) of SARS-CoV-2 vaccines received – in particular for those who had the Janssen COVID-19 Vaccine (Johnson&Johnson). Initially, Janssen COVID-19 Vaccine was the only vaccine for which official German guidance specified that one dose would suffice to be considered fully immunized. All other available vaccines – Comirnaty (BionTech/Pfizer), Spikevax (Moderna) or Vaxzevria (AstraZeneca) – required two doses to attain fully immunized status. However, official guidance changed in January 2022[64]. Those who had received one dose of Janssen COVID-19 Vaccine were required to have a dose of mRNA vaccine (Comirnaty or Spikevax) to retain fully immunized status. This change in guidance took place before we executed our survey (July 2022). Based on the assumption that remembering the number of shots rather than the type of vaccine received (and consequences thereof for immunization status) is easier, we deliberately decided to query the number of SARS-CoV-2 vaccine doses received as our dependent variable for Analysis I.

Supplementary Table 1: Survey Questions

Variables	*Exact question (German original)*	*Exact question (English translation)*
Dependent variables		
Analysis I<br># SARS-CoV-2 vaccine doses (scale: 0–4)	Haben Sie sich gegen Covid-19 impfen lassen?<br>• 0 = Nein, ich bin nicht geimpft<br>• 1 = Ja, Erstimpfung erhalten<br>• 2 = Ja, zwei Impfungen erhalten<br>• 3 = Ja, drei Impfungen erhalten<br>• 4 = Ja, vier Impfungen erhalten	Have you been vaccinated against Covid-19?<br>• 0 = No, I have not been vaccinated.<br>• 1 = Yes, I got one dose.<br>• 2 = Yes, I got two doses.<br>• 3 = Yes, I got three doses.<br>• 4 = Yes, I got four doses.
(crosscheck): Basic immunization (values: 0/1)		*Derived from above:*<br>• *0 = 0 or 1 dose*<br>• *1 = 2 or more doses*
Analysis II<br>Drivers of SARS-CoV-2 immunization decisions, self-perceived importance (This question was only asked to those who had received at least one dose of SARS-CoV-2 vaccine; scale: 0–1)	Wie wichtig waren die folgenden Punkte für Ihre Impfentscheidung?<br>➢ Mich selbst vor Covid-19 schützen<br>➢ Andere vor Covid-19 zu schützen<br>➢ Ärztlicher Ratschlag bzw. allgemeine Impfempfehlung der Ständigen Impfkommission (STIKO)<br>➢ Unproblematische Teilnahme am öffentlichen Geschehen (z.B. Restaurantbesuch)<br>➢ Notwendigkeit der Impfung bei meiner Arbeit<br>➢ Mein Bekannten- und Freundeskreis erwartete es<br><br>Level:<br>• 0 = Völlig unwichtig<br>• 0.25 = Eher unwichtig<br>• 0.5 = Mittel wichtig<br>• 0.75 = Eher wichtig<br>• 1 = Sehr wichtig	How important were the points below to your vaccination decision?<br>➢ Protect myself<br>➢ Protect others<br>➢ Medical advice/ recommendation of the Permanent Vaccination Commission (STIKO)<br>➢ Participation in public events (e.g. visit a restaurant)<br>➢ Vocational mandates, necessity of vaccination at work<br>➢ Peer pressure, expectations of my acquaintances<br><br>Level:<br>• 0 = Highly unimportant<br>• 0.25 = Mostly unimportant<br>• 0.5 = Semi-important<br>• 0.75 = Mostly important<br>• 1 = Highly important
Analysis III<br>How useful do you consider routine pediatric immunization, e.g. against measles/mumps/rubella (MMR)?<br>(scale: 0–1)	Für wie sinnvoll erachten Sie Kinderimpfungen (z.B. gegen Masern/Mumps/Röteln)?<br><br>Level:<br>• 0 = Gar nicht sinnvoll<br>• 0.33 = Eher nicht sinnvoll<br>• 0.67 = Eher sinnvoll<br>• 1 = Sehr sinnvoll	How useful do you consider routine pediatric immunization, e.g. against measles/mumps/ rubella (MMR)?<br><br>Level:<br>• 0 = Not at all meaningful<br>• 0.33 = Rather not meaningful<br>• 0.66 = Rather meaningful<br>• 1 = Very meaningful
Independent variables		
Positive attitudes towards…		
Waldorf education<br>(scale: 0–1)	Wie stehen Sie zu den folgenden Dingen?<br>➢ Waldorfschulen<br><br>Level:<br>• 0 = Sehr negativ<br>• 0.25 = Eher negativ<br>• 0.5 = Neutral<br>• 0.75 = Eher positiv<br>• 1.0 = Sehr positiv	How do you feel about the items below?<br>➢ Waldorf schools<br><br>Level:<br>• 0 = Very negative<br>• 0.25 = Rather negative<br>• 0.50 = Neutral<br>• 0.75 = Rather positive<br>• 1.0 = Very positive
Homeopathy<br>(scale: 0–1)	Wie stehen Sie zu den folgenden Dingen?<br>➢ Homeopathie<br><br>Level:	How do you feel about the items below?<br>➢ Homeopathy<br><br>Level:

	• 0 = Sehr negativ<br>• 0.25 = Eher negativ<br>• 0.5 = Neutral<br>• 0.75 = Eher positiv<br>• 1.0 = Sehr positiv	• 0 = Very negative<br>• 0.25 = Rather negative<br>• 0.50 = Neutral<br>• 0.75 = Rather positive<br>• 1.0 = Very positive
Mainstream medicine (scale: 0–1)	Wie stehen Sie zu den folgenden Dingen?<br>➢ Schulmedizin<br><br>Level:<br>• 0 = Sehr negativ<br>• 0.25 = Eher negativ<br>• 0.5 = Neutral<br>• 0.75 = Eher positiv<br>• 1.0 = Sehr positiv	How do you feel about the items below?<br>➢ Mainstream medicine<br><br>Level:<br>• 0 = Very negative<br>• 0.25 = Rather negative<br>• 0.50 = Neutral<br>• 0.75 = Rather positive<br>• 1.0 = Very positive
Religious denomination	Welcher Religion gehören Sie an?<br>• Keiner, ich bin konfessionslos<br>• Römisch-katholisch<br>• Protestantisch<br>• Evangelische/Evangelikale Freikirche<br>• Orthodoxe Kirche<br>• Jüdisch<br>• Muslimisch<br>• Sonstige, und zwar … (Freitexteingabe)	Which religious denomination do you belong to?<br>• none, I am non-denominational<br>• Roman-Catholic<br>• Protestant<br>• Evangelical Free Church<br>• Orthodox Christian<br>• Jewish<br>• Muslim<br>• Other, und zwar … (free text entry)
Political and psychosocial controls		
Political ideology (scale: 0–1)	Wie würden Sie sich politisch verorten?<br>Klicken Sie die grau unterlegte Leiste an und schieben Sie dann den Punkt an die gewünschte Position (*Schieberegler basierend auf einer 11-Punkte Skala*)	How would you position yourself politically?<br>Click the gray bar and then move the point to the desired position (*slider based on an 11-point scale*)
Left–Right	0 = links – 1 = rechts	0 = left – 1 = right
Green/Alternative/Liberal – Traditional/Authoritarian/Nationalist (GAL–TAN)	0 = ökologisch / alternativ – 1 = traditionell / konservativ	0 = ecological / alternative – 1 = traditional / conservative
Voting intention	Wenn am Sonntag Bundestagswahl wäre, welche Partei würden Sie mit Ihrer Zweitstimme wählen?	If there were a federal election on Sunday, which party would you vote for with your Second Vote?+
CDU/CSU (Christian democrats)<br>SPD (Social democrats)<br>Greens<br>FDP (Liberals)<br>Left-Party (Socialist)<br>AfD (Populist right wing)<br>Others	• CDU / CSU<br>• SPD<br>• Grüne<br>• FDP<br>• Linke<br>• AfD<br>• Andere, und zwar … (Freitexteingabe)	• CDU / CSU++<br>• SPD<br>• Greens<br>• FDP<br>• Left-Party<br>• AfD<br>• Others, namely … (Free text entry)
Solidarity<br><br>*The solidarity score is the arithmetic mean of a battery of four questions on attitudes towards social and personal solidarity*<br>(scale: 0–1)	Nun möchten wir Ihnen ein paar Fragen zu Ihren Einstellungen stellen. Wie stehen Sie zu den folgenden Aussagen?<br>➢ Wer viel Steuern zahlt, tut damit schon genug für die Gesellschaft.<br>➢ Die Gesellschaft ist auf freiwilliges Engagement der Bevölkerung angewiesen.<br>➢ Es ist wichtiger, für das Wohl der Gemeinschaft zu arbeiten, als für das eigene Wohl.<br>➢ Wenn ein Fremder auf meine Unterstützung angewiesen ist, versuche	Now we would like to ask you a few questions about your attitudes. How do you feel about the following statements?<br>➢ Those who pay a lot of taxes are already doing enough for society. (*reversed for the calculation of the solidarity score)*<br>➢ Society depends on the voluntary commitment of the population.<br>➢ It is more important to work for the good of the community than for your own good.<br>➢ If a stranger is in need of my support, I try to give it to them.

	ich, sie ihm zu geben.<br>Level:<br>• 0 = Stimme überhaupt nicht zu<br>• 0.25 = Stimme eher nicht zu<br>• 0.5 = Neutral<br>• 0.75 = Stimme eher zu<br>• 1 = Stimme vollkommen zu	Level:<br>• 0 = Completely disagree<br>• 0.25 = Rather disagree<br>• 0.5 = Neutral<br>• 0.75 = Rather agree<br>• 1 = Completely agree
Big Five<br>*Personality is measured according to the five-factor model using the validated Big Five Inventory (BFI 10) developed by Rammstedt et al.[65]. The Big Five Dimensions scores are the arithmetic mean of each of two personality questions, one of which is formulated negatively and is therefore included in the calculation in reverse order.*	An dieser Stelle geht es um unterschiedliche Eigenschaften, die eine Person haben kann. Inwiefern treffen die folgenden Aussagen auf Sie zu?	At this point, we are talking about different characteristics that a person can have. To what extent do the following statements apply to you?
Agreeableness (scale: 0–1)	➢ Ich schenke anderen leicht Vertrauen, glaube an das Gute im Menschen.<br>➢ Ich neige dazu, andere zu kritisieren.	➢ I see myself as someone who is generally trusting.<br>➢ I see myself as someone who tends to find fault with others (*reversed for the calculation*)
Conscientiousness (scale: 0–1)	➢ Ich bin bequem, neige zur Faulheit.<br>➢ Ich erledige Aufgaben gründlich.	➢ I see myself as someone who tends to be lazy (*reversed for the calculation*).<br>➢ I see myself as someone who does a thorough job.
Extraversion (scale: 0–1)	➢ Ich bin eher zurückhaltend, reserviert.<br>➢ Ich gehe aus mir heraus, bin gesellig.	➢ I see myself as someone who is reserved (*reversed for the calculation*).<br>➢ I see myself as someone who is outgoing, sociable.
Neuroticism (scale: 0–1)	➢ Ich bin entspannt, lasse mich durch Stress nicht aus der Ruhe bringen.<br>➢ Ich werde leicht nervös und unsicher.	➢ I see myself as someone who is relaxed, handles stress well (*reversed for the calculation*).<br>➢ I see myself as someone who gets nervous easily.
Openness (scale: 0–1)	➢ Ich habe nur wenig künstlerisches Interesse.<br>➢ Ich habe eine aktive Vorstellungskraft, bin fantasievoll.	➢ I see myself as someone who has few artistic interests (*reversed for the calculation*).<br>➢ I see myself as someone who has an active imagination.
	Level:<br>• 0 = Trifft überhaupt nicht zu<br>• 0.25 = Trifft eher nicht zu<br>• 0.5 = Trifft mittelmäßig zu<br>• 0.75 = Trifft eher zu<br>• 1 = Trifft voll und ganz zu	Level:<br>• 0 = Not true at all<br>• 0.25 = Rather not true<br>• 0.5 = Medium true<br>• 0.75 = Rather true<br>• 1 = Completely true
State (Bundesland)	In welchem Bundesland leben Sie?<br>• Baden-Württemberg<br>• Bayern<br>• Berlin<br>• Brandenburg<br>• Bremen<br>• Hamburg<br>• Hessen<br>• Mecklenburg-Vorpommern<br>• Niedersachsen<br>• Nordrhein-Westfalen<br>• Rheinland-Pfalz<br>• Saarland<br>• Sachsen	In which state do you live?<br>• Baden-Wuerttemberg<br>• Bavaria<br>• Berlin<br>• Brandenburg<br>• Bremen<br>• Hamburg<br>• Hesse<br>• Mecklenburg-Vorpommern<br>• Lower Saxony<br>• North Rhine-Westphalia<br>• Rhineland Palatinate<br>• Saarland<br>• Saxony

	• Sachsen-Anhalt<br>• Schleswig-Holstein<br>• Thüringen<br>• Ich lebe im Ausland, und zwar … (Freitexteingabe)	• Saxony-Anhalt<br>• Schleswig Holstein<br>• Thuringia<br>• Foreign country
Gender	Welches Geschlecht haben Sie?<br>• Männlich<br>• Weiblich<br>• Nicht binär	What is your gender?<br>• Male<br>• Female<br>• Non-binary
Age group<br>18-30<br>31-45<br>46-60<br>> 60	Wie alt sind Sie?<br>• 12 – 99 Jahre	How old are you?<br>• 12 – 99 years (*the answers were grouped into the 4 age brackets, participants younger than 18 years were excluded from the analysis*)
Educational attainment	Was ist Ihr höchster allgemeinbildender Schulabschluss?	What is the highest level of general education you have completed?
Low	• Kein Schulabschluss<br>• Volks-/ Hauptschul-abschluss bzw. Poly-technische Oberschule mit Abschluss 8. oder 9. Klasse<br>• Sonstiges	• No school-leaving certificate<br>• Secondary school diploma or polytechnic high school diploma with completion of 8th or 9th grade<br>• Other
Middle	• Mittlere Reife, Realschulabschluss bzw. Polytechnische Oberschule mit Abschluss 10. Klasse	• Secondary school diploma or polytechnic high school diploma with completion of 10th grade
High	• Fachabitur<br>• Abitur bzw. Erweiterte Oberschule mit Abschluss 12. Klasse (Hochschulreife)<br>• Noch in der Schule	• Vocational high school diploma<br>• High school diploma or extended high school with completion of grade 12 (university entrance qualification)<br>• Still at school
Household income	Wie beurteilen Sie Ihr gegenwärtiges Haushaltseinkommen? Mit meinem/unserem Einkommen kann ich/können wir...	How would you rate your current household income? With my/our income I/we can...
	Level:<br>• Eigentlich gar nicht zurechtkommen<br>• Nur schwer zurechtkommen<br>• Zurechtkommen<br>• Bequem leben<br>• Sehr bequem leben<br>• Trifft nicht zu (noch kein eigenes Haushaltseinkommen)	Level:<br>• I find it very difficult to make ends meet<br>• I find it quite difficult to make ends meet<br>• I can make ends meet<br>• I can live quite comfortably on the income<br>• I can live very comfortably on the income<br>• Does not apply (no own household income)

Note: [+] In Germany, voters have two votes for the election of the German parliament, the Bundestag. With the so called “First Vote” (*Erststimme*) voters determine a direct candidate of their constituency, with the “Second Vote” (*Zweitstimme*) electors vote for a party. This second vote determines the distribution of seats in the Bundestag and is therefore more important than the First Vote.
[++] CDU (Christian Democratic Union) and CSU (Christian Social Union in Bavaria) are sister parties. Together they are called the Union. Both always form a single fraction in the Bundestag. While the CSU operates only in Bavaria, the CDU operates only in the 15 other German states. In the survey CDU and CSU were queried as separate items. In the analysis both were combined.

Supplementary Table 2: Missing values – item non-response

	Missings #	Missings %
**Dependent variables**		
Analysis I		
# SARS-CoV-2 vaccine doses	429	5.01
(crosscheck): Basic immunization (dummy variable: 2 or more doses)	429	5.01
Analysis II+		
Drivers of SARS-CoV-2 immunization decisions, self-perceived importance		
Protecting self	1,156	13.49
Protect others	1,171	13.67
Medical advice/recommendation of the Permanent Vaccination Commission (STIKO)	1,166	13.61
Participation in public events	1,229	14.34
Vocational mandates	1,168	13.63
Peer pressure	1,189	13.88
Analysis III		
How useful do you consider routine pediatric immunization, e.g. against measles/mumps/rubella (MMR)?	516	6.02
**Independent variables**		
Positive attitudes towards…	321	3,75
Waldorf education	317	3.70
Homeopathy	330	3.85
Mainstream medicine	542	6.33
Religious denomination	321	3.75
**Political & psychosocial controls**		
Political ideology		
Left–Right	525	6.13
Green/Alternative/Liberal (GAL) –Traditional/Authoritarian/Nationalist (TAN)	630	7.35
Voting intention	544	6.35
Solidarity	310	3.62
Big 5		
Agreeableness	430	5.02
Conscientiousness	428	5.00
Extraversion	432	5.04
Neuroticism	422	4.93
Openness	429	5.01
**Socio-demographic controls**		
State (Bundesland)	538	6.28
Gender	571	6.66
Age group	527	6.15
Educational attainment	530	6.19
Household income	542	6.33

Note: N = 8,568 (participants >= 18 years who started the survey).
+ Only those participants who received at least one dose of SARS-CoV-2 vaccine were asked about the drivers of their decision to get immunized. This explains the higher number of missing values for these variables.

Supplementary Table 3: Descriptive statistics – comparison of the original dataset and the final dataset used in the regression models, reduced via listwise deletion (unweighted and weighted data)

	Original data			Reduced datset for regression analysis (listwise deletion)				
					Raw data – not weighted		Weighted data	
Dependent variables	N	Mean	Sd	N	Mean	Sd	Mean	Sd
Analysis I								
# SARS-CoV-2 vaccine doses (scale: 0–4)	8,168	2.77	0.98	7,391	2.77	0.97	2.81	0.96
(crosscheck): Basic immunization (dummy variable: 2 or more doses)	8,168	n (%)		7,391	n (%)		n (%)	
0 or 1 dose		789 (9.66)			700 (9.47)		658.49 (8.91)	
2 or more doses		7,379 (90.34)			6,691 (90.53)		6,732.51 (91.09)	
Analysis II								
Drivers of SARS-CoV-2 immunization decisions, self-perceived importance (scale: 0–1)								
Protecting self	7,441	0.84	0.27	6,657	0.84	0.27	0.85	0.26
Protect others	7,426	0.78	0.29	6,657	0.78	0.29	0.78	0.29
Medical advice/recommendation of the Permanent Vaccination Commission (STIKO)	7,429	0.60	0.33	6,657	0.60	0.33	0.61	0.33
Participation in public events	7,431	0.58	0.33	6,657	0.57	0.33	0.58	0.33
Vocational mandates	7,367	0.37	0.37	6,657	0.37	0.37	0.38	0.37
Peer pressure	7,408	0.23	0.28	6,657	0.23	0.28	0.23	0.28
Analysis III								
How useful do you consider routine pediatric immunization, e.g. against measles/mumps/rubella (MMR)? (scale: 0–1)	8,081	0.91	0.21	7,391	0.91	0.20	0.91	0.20
Independent variables								
Positive attitudes towards…								
Waldorf education (scale: 0–1)	8,268	0.40	0.25	7,391	0.40	0.25	0.40	0.25
Homeopathy (scale: 0–1)	8,281	0.40	0.31	7,391	0.39	0.31	0.42	0.30
Mainstream medicine (scale: 0–1)	8,277	0.77	0.20	7,391	0.77	0.20	0.77	0.20
Religious denomination	8,055	n (%)		7,391	n (%)		n (%)	
No denomination		4,145 (51.46)			3,808 (51.52)		3,661.50 (49.54)	
Roman-Catholic		1,584 (19.66)			1,464 (19.81)		1,586.64 (21.47)	
Protestant		1,378 (17.11)			1,252 (16.94)		1,264.75 (17.11)	
Evangelical Free Church		702 (8.72)			651 (8.81)		695.04 (9.40)	
Orthodox Christian		24 (0.30)			21 (0.28)		20.54 (0.28)	
Jewish		13 (0.16)			11 (0.15)		9.38 (0.13)	
Muslim		37 (0.46)			36 (0.49)		34.00 (0.46)	
Other		172 (2.14)			148 (2.00)		119.16 (1.61)	
Political & psychosocial controls								
Political ideology								
Left – Right (scale: 0–1)	8,073	0.42	0.20	7,391	0.41	0.20	0.44	0.20
Green/Alternative/Liberal (GAL) - Traditional/Authoritarian/Nationalist (TAN) (scale: 0–1)	7,968	0.45	0.24	7,391	0.45	0.24	0.49	0.24
Voting intention	8,053	n (%)		7,391	n (%)		n (%)	
CDU/CSU (Christian democrats)		1,188 (14.75)			1,076 (14.56)		1,773.05 (23.99)	
SPD (Social democrats)		971 (12.06)			888 (12.01)		1,348.04 (18.24)	
Greens		2,679 (33.27)			2,505 (33.89)		1,868.04 (25.27)	
FDP (Liberals)		675 (8.38)			631 (8.54)		575.06 (7.78)	
Left-Party (Socialist)		569 (7.07)			530 (7.17)		406.09 (5.49)	
AfD (Populist right wing)		616 (7.65)			545 (7.37)		787.00 (10.65)	
Others		1,355 (16.83)			1,216 (16.45)		633.72 (8.57)	
Solidarity (scale: 0–1)	8,288	0.65	0.17	7,391	0.66	0.17	0.65	0.17
Big Five								
Agreeableness (scale: 0–1)	8,167	0.53	0.19	7,391	0.53	0.19	0.53	0.19

Conscientiousness (scale: 0–1)	8,169	0.67	0.20	7,391	0.67	0.20	0.69	0.20
Extraversion (scale: 0–1)	8,165	0.52	0.25	7,391	0.52	0.25	0.54	0.24
Neuroticism (scale: 0–1)	8,176	0.42	0.22	7,391	0.42	0.22	0.43	0.22
Openness (scale: 0–1)	8,168	0.62	0.23	7,391	0.62	0.23	0.62	0.23
Socio-demographic controls								
State (Bundesland)	8,059	n (%)		7,391	n (%)		n (%)	
Baden-Wuerttemberg		1,703 (21.13)			1,561 (21.12)		993.71 (13.44)	
Bavaria		1,107 (13.74)			1,020 (13.80)		1,179.43 (15.96)	
Berlin		327 (4.06)			306 (4.14)		315.86 (4.27)	
Brandenburg		172 (2.13)			160 (2.16)		238.27 (3.22)	
Bremen		381 (4.73)			357 (4.83)		61.85 (0.84)	
Hamburg		156 (1.94)			140 (1.89)		158.70 (2.15)	
Hesse		476 (5.91)			434 (5.87)		552.53 (7.48)	
Mecklenburg-Vorpommern		114 (1.41)			106 (1.43)		150.62 (2.04)	
Lower Saxony		732 (9.08)			660 (8.93)		684.24 (9.26)	
North Rhine-Westphalia		1,550 (19.23)			1,429 (19.33)		1,581.45 (21.40)	
Rhineland Palatinate		325 (4.03)			294 (3.98)		358.32 (4.85)	
Saarland		76 (0.94)			68 (0.92)		86.63 (1.17)	
Saxony		292 (3.62)			269 (3.64)		372.90 (5.05)	
Saxony-Anhalt		108 (1.34)			97 (1.31)		163.03 (2.21)	
Schleswig Holstein		256 (3.18)			228 (3.08)		263.70 (3.57)	
Thuringia		192 (2.38)			178 (2.41)		215.64 (2.92)	
Foreign country		92 (1.14)			84 (1.14)		14.12 (0.19)	
Gender	8,026	n (%)		7,391	n (%)		n (%)	
Male		5,014 (62.47)			4,649 (62.90)		3,687.31 (49.89)	
Female		2,966 (36.95)			2,699 (36.52)		3,647.77 (49.35)	
Non-binary		46 (0.57)			43 (0.58)		55.92 (0.76)	
Age group	8,041	n (%)		7,391	n (%)		n (%)	
18-30		1,474 (18.33)			1,410 (19.08)		1,279.86 (17.32)	
31-45		1,930 (24.00)			1,824 (24.68)		1,624.58 (21.98)	
46-60		2,482 (30.87)			2,267 (30.67)		2,051.58 (27.76)	
> 60		2,155 (26.80)			1,890 (25.57)		2,434.98 (32.95)	
Educational attainment	8,068	n (%)		7,391	n (%)		n (%)	
		345 (4.28)			295 (3.99)		347.47 (4.70)	
		1,638 (20.30)			1,468 (19.86)		1,643.09 (22.23)	
		6,085 (75.42)			5,628 (76.15)		5,400.44 (73.07)	
Household income	8,055	n (%)		7,391	n (%)		n (%)	
		192 (2.38)			172 (2.33)		185.45 (2.51)	
		616 (7.65)			559 (7.56)		555.20 (7.51)	
		2,408 (29.89)			2,210 (29.90)		2,286.45 (30.94)	
		3,337 (41.43)			3,075 (41.60)		3,059.45 (41.39)	
		1,396 (17.33)			1,279 (17.30)		1,234.06 (16.70)	
		106 (1.32)			96 (1.30)		70.40 (0.95)	

Note: The comparison between the original dataset (using all existing information from the survey) and the dataset reduced by listwise deletion (dropping all cases that have at least one missing value), the latter being the dataset used in the regression analyses, shows only marginal differences. The means and standard deviations are virtually identical. Therefore, item non-response does not introduce systematic bias and a complete cases regression analysis is appropriate. The weighted data uses a proportional iterative fitting (raking) procedure to adjust the data of the participants to the real distribution of the following factors in the German population: age group, gender, state and voting intention. The official population data are derived from the Federal Statistical Office of Germany (Genesis database: https://www-genesis.destatis.de/genesis/online) and for voting intention the results of the German federal election on 26 September 2021 as reported by the Federal Returning Officer (https://www.bundeswahlleiterin.de/en/bundestagswahlen/2021.html) were used as basis for the raking. We apply this weighted data in all regression models in the article.

Supplementary Table 4: (a) Correlations between the main independent variables, and (b) exploratory factor analysis

a) Correlations (Pearsons r)

Attitude towards…	Waldorf education	Homeopathy	Mainstream medicine
Waldorf education	1.000		
Homeopathy	0.342	1.000	
Mainstream medicine	-0.162	-0.302	1.000

b) Exploratory factor analysis

Factor	Eigenvalue
1	0.68329
2	-0.05404
3	-0.23394

Factor loadings	Factor 1	Uniqueness
Waldorf education	0.4553	0.7927
Homeopathy	0.5549	0.6921
Mainstream medicine	-0.4100	0.8319

Note: N = 7,391, Data weighted by state, age group, gender and voting intention to better represent the German population. Method for factor analysis: principal factors, unrotated.

Supplementary Table 5: Internal consistency of survey instruments

a) Solidarity Index

Pearson correlations

	Solidarity item 1	Solidarity item 2	Solidarity item 3	Solidarity item 4
Solidarity item 1	1.0000			
Solidarity item 2	-0.2915	1.0000		
Solidarity item 3	-0.3737	0.2879	1.0000	
Solidarity item 4	-0.2935	0.2928	0.3239	1.0000

Factor analysis - Principal Components method
(Overall Kaiser-Meyer-Olkin measure of sampling adequacy: 0.7279)

	Eigenvalue		Factor Loading	Uniqueness
Factor 1	1.98607	Solidarity item 1	-0.7135	0.4909
Factor 2	0.714866	Solidarity item 2	0.6843	0.5317
Factor 3	0.684659	Solidarity item 3	0.7326	0.4633
Factor 4	0.614405	Solidarity item 4	0.6870	0.5280

Cronbachs Alpha	0.6549

Note: N = 7,391, Data weighted by state, age group, gender and voting intention to better represent the German population.

- Solidarity item 1: Those who pay a lot of taxes are already doing enough for society.
- Solidarity item 2: Society depends on the voluntary commitment of the population.
- Solidarity item 3: It is more important to work for the good of the community than for your own good.
- Solidarity item 4: If a stranger is in need of my support, I try to give it to them.

b) Big 5

Pearson correlations

	Item 1	Item 2	Item 3	Item 4	Item 5	Item 6	Item 7	Item 8	Item 9	Item10
Item 1	1.0000									
Item 2	-0.0734	1.0000								
Item 3	0.1975	0.0251	1.0000							
Item 4	-0.0511	0.0589	-0.0477	1.0000						
Item 5	0.0787	-0.0359	0.0821	-0.0004	1.0000					
Item 6	-0.5787	0.1720	-0.1581	0.1337	-0.0541	1.0000				
Item 7	-0.0225	-0.0878	0.1659	-0.1259	0.0438	0.0186	1.0000			
Item 8	-0.0194	-0.0288	-0.2696	0.0960	-0.0285	0.0968	-0.0055	1.0000		
Item 9	0.3333	0.0510	0.2458	-0.4088	0.0387	-0.2587	0.1024	-0.1020	1.0000	
Item10	-0.1536	0.0449	-0.0436	0.0667	-0.2999	0.2024	0.0391	0.0944	-0.0927	1.0000

Factor Analysis - Principal Factor method
(Overall Kaiser-Meyer-Olkin measure of sampling adequacy: 0.5825)

	Eigenvalue	Factor Loadings (Rotation: orthogonal varimax, Kaiser off)						Uniqueness
			Factor 1	Factor 2	Factor 3	Factor 4	Factor 5	0.4950
Factor 1	1.49843	Item 1	0.6988	0.0917	0.0528	-0.0369	0.0395	0.8745
Factor 2	0.53031	Item 2	-0.1453	0.0221	0.0818	0.0467	0.3067	0.7253
Factor 3	0.35757	Item 3	0.1877	0.1474	0.4617	-0.0352	-0.0091	0.6994
Factor 4	0.25814	Item 4	-0.0766	-0.5330	-0.0088	0.0232	0.0870	0.8051
Factor 5	0.16240	Item 5	0.0731	-0.0009	0.0654	-0.4256	-0.0336	0.5077
		Item 6	-0.6829	-0.1029	-0.0366	0.0717	0.0695	0.8626
		Item 7	-0.0482	0.1784	0.1627	0.0103	-0.2378	0.8297
		Item 8	-0.0519	-0.1057	-0.3607	0.0799	-0.0387	0.5789
		Item 9	0.3120	0.5434	0.1482	-0.0109	0.0635	0.7599
		Item10	-0.1996	-0.0454	-0.0226	0.4408	-0.0099	0.4950

Cronbachs Alpha		Positive item	Reversed item
Extraversion	0.7321	Item 1: I see myself as someone who is reserved	Item 6: I see myself as someone who is outgoing, sociable.
Neuroticism	0.5801	Item 9: I see myself as someone who gets nervous easily.	Item 4: I see myself as someone who is relaxed, handles stress well.

Conscientiousness	0.4092	Item 8: I see myself as someone who does a thorough job.	Item 3: I see myself as someone who tends to be lazy.
Openness	0.4515	Item 10: I see myself as someone who has an active imagination.	Item 5: I see myself as someone who has few artistic interests.
Agreeableness	0.1601	Item 2: I see myself as someone who is generally trusting.	Item 7: I see myself as someone who tends to find fault with others.

Note: N = 7,391; Data weighted by state, age group, gender and voting intention to better represent the German population.

Supplementary Table 6: Overview of main assumptions and findings stratified by Analysis

	Analysis I	Analysis II	Analysis III
Research Questions	What are the effects of attitudes towards Waldorf education, homeopathy and mainstream medicine, and religious denomination on SARS-CoV-2 immunization status (number of doses)?	What are the main drivers of the decision to have at least one dose of SARS-CoV-2 vaccine?	Are attitudes towards routine pediatric immunization (MMR) similarly driven by the effects observed in Analysis I?
Assumptions	*Positively* correlated with the number of SARS-CoV-2 vaccine doses:<br>• Positive attitudes towards mainstream medicine<br>• A higher relative score on the personality traits openness, neuroticism and conscientiousness<br>• A higher relative score in relative terms on the solidarity index<br><br>*Negatively* correlated with the number of SARS-CoV-2 vaccine doses:<br>• Positive attitudes towards Waldorf education and homeopathy<br>• Right-wing populist political ideology<br><br>No effect assumed:<br>• Religious denomination	For the outcome of having a (further dose of) SARS-CoV-2 vaccine…<br>• Voluntary considerations compel individuals who have positive attitudes towards mainstream medicine<br>• Vaccine mandates compel individuals who have positive attitudes towards Waldorf education and homeopathy	The effects of our independent variables and controls on SARS-CoV-2 immunization status (Analysis I) are similar to the effects of our independent variables on attitudes towards routine pediatric immunization (MMR).<br><br>*Positively* correlated with the attitude towards MMR:<br>• Positive attitudes towards mainstream medicine<br><br>*Negatively* correlated with the attitude towards MMR:<br>• Positive attitudes towards Waldorf education and homeopathy
Principal findings	On the number of SARS-CoV-2 vaccine doses, the effect of…<br>• positive attitude towards Waldorf education and homeopathy is moderately to strongly *negative*.<br>• positive attitude towards mainstream medicine is strongly *positive*.<br>• religious denomination is marginal.	To have a (further) vaccine dose…<br><br>• primarily *voluntary considerations* drove the reasoning of individuals with positive views of mainstream medicine, and those who are more solidary and conscientious.<br>• primarily *external pressures* drove the immunization decision for individuals with positive views of homeopathy and Waldorf education (the latter to a slightly lesser extent), those who are more conservative / right-wing and highly extraverted.	• Overall the impact of Waldorf education, homeopathy and mainstream medicine on attitudes towards childhood immunization is very much comparable to that on number of SARS-CoV-2 vaccine doses.<br>• A stronger sense of solidarity and being more politically traditional/authoritarian/nationalist were the only two variables that had a stronger impact on positive attitudes towards childhood immunization vis-à-vis number of SARS-CoV-2 vaccine doses.
Other findings	• being a supporter of (liberal) FDP, (right wing populist) AfD or *Other* parties is moderately to strongly *negative*.<br>• personality trait neuroticism is moderately *positive*.	• religious denomination did not have a strong impact on immunization motives.	• Support for AfD and protest parties is moderately negatively correlated with attitudes towards childhood immunization.<br>• Age bracket 60+ had a reverse (negative) effect on attitudes towards childhood immunization while being moderately positive on SARS-CoV-2 vaccine doses.

Supplementary Table 7: Analysis I – Impact on SARS-CoV-2 vaccine uptake (unstandardized b-coefficients from multiple linear regression): separate models by groups of variables (models M1.1-M1.4) and full model (M1.5); complete regression table presenting also controls for state, gender, age group, education level and household income

Dependent variable: # SARS-CoV-2 vaccine doses (scale: 0–4)	M1.1 Medicine and school attitudes	M1.2 Religious denomination	M1.3 Political factors	M1.4 Psychological factors	M1.5 Full model
Positive attitudes towards…					
Waldorf education (scale: 0–1)	-0.0403 [-0.147,0.0667]				-0.134** [-0.232,-0.0355]
Homeopathy (scale: 0–1)	-0.505*** [-0.602,-0.408]				-0.375*** [-0.461,-0.289]
Mainstream medicine (scale: 0–1)	1.482*** [1.316,1.648]				1.034*** [0.884,1.185])
Religious denomination (Reference: no denomination)					
Roman-Catholic		0.174*** [0.107,0.242]			0.0732* [0.0166,0.130]
Protestant		0.153*** [0.0824,0.223]			0.0304 [-0.0290,0.0897]
Evangelical Free Church		0.0628 [-0.0311,0.157]			0.0247 [-0.0562,0.106]
Orthodox		-0.589 [-1.240,0.0620]			-0.399 [-0.809,0.0105]
Jewish		-0.138 [-1.232,0.957]			0.329 [-0.547,1.204]
Muslim		-0.179 [-0.569,0.211]			-0.0677 [-0.411,0.276]
Other		-0.172 (-1.31)			-0.0535 (-0.49)
Left = 0 / Right = 1 (scale: 0–1)			-0.127 [-0.345,0.0903]		-0.0378 [-0.246,0.170]
GAL = 0 / TAN = 1 (scale: 0–1)			0.137 [-0.0210,0.294]		-0.0194 [-0.170,0.131]
Voting Intention (Reference: CDU/CSU: Christian democrats)					
SPD (Social democrats)			0.0697* [0.00179,0.138]		0.0509 [-0.0160,0.118]
Greens			0.0599		0.0397

			[-0.0164,0.136]		[-0.0355,0.115]
FDP (Liberals)			-0.297***		-0.232***
			[-0.396,-0.198]		[-0.329,-0.136]
Left-Party (Socialists)			-0.223**		-0.160*
			[-0.365,-0.0799]		[-0.295,-0.0261]
AfD (Populist radical-right)			-1.365***		-1.158***
			[-1.498,-1.233]		[-1.287,-1.030]
Other (including Covid-19 protest party)			-0.926***		-0.717***
			[-1.070,-0.782]		[-0.847,-0.587]
Solidarity				1.318***	0.173*
(scale: 0–1)				[1.140,1.496]	[0.00368,0.343]
Agreeableness (Big 5)				-0.139	-0.149*
(scale: 0–1)				[-0.283,0.00448]	[-0.272,-0.0255]
Conscientiousness (Big 5)				-0.106	0.0283
(scale: 0–1)				[-0.247,0.0358]	[-0.0939,0.150]
Extraversion (Big 5)				0.0360	0.0954
(scale: 0–1)				[-0.0770,0.149]	[-0.00144,0.192]
Neuroticism (Big 5)				0.296***	0.247***
(scale: 0–1)				[0.169,0.422]	[0.139,0.355]
Openness (Big 5)				-0.102	-0.0626
(scale: 0–1)				[-0.216,0.0120]	[-0.162,0.0372]
State (Reference: North Rhine-Westphalia)					
Baden-Wuerttemberg	-0.108**	-0.112**	-0.163***	-0.0677	-0.148***
	[-0.186,-0.0300]	[-0.194,-0.0304]	[-0.234,-0.0912]	[-0.147,0.0118]	[-0.218,-0.0786]
Bavaria	-0.195***	-0.221***	-0.158***	-0.202***	-0.148***
	[-0.278,-0.113]	[-0.310,-0.132]	[-0.235,-0.0800]	[-0.290,-0.115]	[-0.221,-0.0756]
Berlin	-0.285***	-0.192*	-0.174*	-0.216**	-0.194**
	[-0.424,-0.147]	[-0.346,-0.0388]	[-0.307,-0.0414]	[-0.361,-0.0702]	[-0.318,-0.0697]
Brandenburg	-0.0701	-0.00565	0.0756	-0.0516	0.0656
	[-0.219,0.0791]	[-0.164,0.153]	[-0.0710,0.222]	[-0.207,0.104]	[-0.0766,0.208]
Bremen	0.278**	0.256*	0.0792	0.281*	0.150
	[0.0753,0.481]	[0.00776,0.503]	[-0.121,0.280]	[0.0422,0.520]	[-0.0276,0.328]
Hamburg	-0.0577	-0.00527	0.00526	-0.0319	-0.00399
	[-0.219,0.104]	[-0.189,0.179]	[-0.167,0.178]	[-0.222,0.158]	[-0.163,0.155]

Hesse	-0.167**	-0.174**	-0.113*	-0.160**	-0.101*
	[-0.275,-0.0588]	[-0.291,-0.0559]	[-0.217,-0.00958]	[-0.275,-0.0448]	[-0.199,-0.00315]
Mecklenburg-Vorpommern	-0.331**	-0.295*	-0.236*	-0.382***	-0.212*
	[-0.548,-0.114]	[-0.533,-0.0577]	[-0.459,-0.0123]	[-0.608,-0.157]	[-0.423,-0.00167]
Lower Saxony	0.0874*	0.114*	-0.0396	0.0867	-0.0121
	[0.00274,0.172]	[0.0249,0.204]	[-0.117,0.0377]	[-0.00118,0.175]	[-0.0890,0.0648]
Rhineland Palatinate	-0.111	-0.178*	-0.145*	-0.152*	-0.0987
	[-0.242,0.0206]	[-0.322,-0.0348]	[-0.267,-0.0227]	[-0.290,-0.0144]	[-0.211,0.0137]
Saarland	-0.00454	-0.0428	-0.0356	-0.00987	-0.0634
	[-0.180,0.171]	[-0.260,0.175]	[-0.216,0.145]	[-0.214,0.195]	[-0.224,0.0973]
Saxony	-0.501***	-0.449***	-0.342***	-0.473***	-0.336***
	[-0.647,-0.354]	[-0.608,-0.291]	[-0.474,-0.210]	[-0.622,-0.325]	[-0.463,-0.210]
Saxony-Anhalt	-0.195	-0.156	-0.0863	-0.163	-0.0651
	[-0.396,0.00650]	[-0.373,0.0612]	[-0.278,0.106]	[-0.370,0.0443]	[-0.255,0.125]
Schleswig Holstein	0.0860	0.111	0.0236	0.0706	0.0488
	[-0.0399,0.212]	[-0.0203,0.242]	[-0.0791,0.126]	[-0.0517,0.193]	[-0.0557,0.153]
Thuringia	-0.238**	-0.183*	-0.0909	-0.230**	-0.111
	[-0.402,-0.0741]	[-0.359,-0.00670]	[-0.254,0.0722]	[-0.403,-0.0568]	[-0.262,0.0410]
Foreign country	-0.426***	-0.444***	-0.275**	-0.461***	-0.258**
	[-0.665,-0.187]	[-0.700,-0.189]	[-0.475,-0.0749]	[-0.708,-0.214]	[-0.453,-0.0636]
Gender (Reference: Male)					
Female	0.0852**	-0.0269	-0.0993***	-0.104***	-0.0275
	[0.0333,0.137]	[-0.0820,0.0281]	[-0.151,-0.0478]	[-0.160,-0.0484]	[-0.0799,0.0248]
Non-binary	-0.0148	0.0318	0.103	-0.122	0.0194
	[-0.346,0.317]	[-0.317,0.380]	[-0.223,0.428]	[-0.472,0.228]	[-0.274,0.313]
Age group (Reference: 18-30)					
31-45	-0.0837*	-0.116**	-0.0599	-0.0729	-0.0262
	[-0.156,-0.0113]	[-0.193,-0.0387]	[-0.129,0.00903]	[-0.148,0.00239]	[-0.0923,0.0399]
46-60	-0.0252	-0.127**	-0.0368	-0.0627	0.0562
	[-0.0986,0.0482]	[-0.204,-0.0494]	[-0.106,0.0329]	[-0.139,0.0135]	[-0.0118,0.124]
> 60	0.329***	0.232***	0.251***	0.228***	0.351***
	[0.257,0.400]	[0.157,0.307]	[0.183,0.319]	[0.153,0.304]	[0.283,0.419]
Educational attainment (Reference: Low)					
Middle	-0.0575	-0.0246	-0.0688	-0.0242	-0.0708
	[-0.196,0.0813]	[-0.175,0.126]	[-0.197,0.0599]	[-0.169,0.120]	[-0.193,0.0510]

High	-0.0632	0.0457	-0.0440	0.0111	-0.109
	[-0.194,0.0678]	[-0.0962,0.188]	[-0.165,0.0773]	[-0.124,0.147]	[-0.224,0.00720]
Household income (Reference: I find it very difficult to make ends meet)					
I find it quite difficult to make ends meet	0.113	0.137	0.119	0.166	0.112
	[-0.121,0.347]	[-0.131,0.405]	[-0.111,0.348]	[-0.0878,0.420]	[-0.0962,0.320]
I can make ends meet	0.286**	0.342**	0.155	0.344**	0.156
	[0.0758,0.497]	[0.0964,0.588]	[-0.0541,0.365]	[0.113,0.575]	[-0.0314,0.344]
I can live quite comfortably on the income	0.382***	0.498***	0.223*	0.476***	0.184
	[0.174,0.590]	[0.255,0.742]	[0.0155,0.430]	[0.248,0.705]	[-0.00175,0.371]
I can live very comfortably on the income	0.286**	0.481***	0.182	0.468***	0.0977
	[0.0733,0.499]	[0.233,0.729]	[-0.0297,0.394]	[0.234,0.702]	[-0.0932,0.289]
does not apply (no own household income)	0.229	0.333	0.113	0.273	0.0514
	[-0.0729,0.530]	[-0.0169,0.683]	[-0.186,0.411]	[-0.0567,0.602]	[-0.217,0.320]
Constant	1.631***	2.409***	2.982***	1.730***	2.167***
	[1.347,1.915]	[2.129,2.690]	[2.715,3.248]	[1.413,2.047]	[1.836,2.498]
Adj. R^2	0.216	0.0844	0.303	0.135	0.379

Note: N = 7,391. Data weighted by state, age group, gender and voting intention to better represent the German population.
95% confidence intervals in brackets. * p < 0.05, ** p < 0.01, *** p < 0.001; GAL, green/alternative/liberal; TAN, traditional/authoritarian/nationalist.

Supplementary Table 8: Analysis I crosscheck – Impact on SARS-CoV-2 vaccine uptake (unstandardized b-coefficients from stepwise multiple linear regression)

Dependent variable: # SARS-CoV-2 vaccine doses (scale: 0–4)	M1a	M1b + left-right	M1c + GAL/TAN	M1d + voting intention	M1e + solidarity index	M1.5 Full model
Positive attitudes towards…						
Waldorf education (scale: 0–1)	-0.0528 [-0.160,0.0545]	-0.117* [-0.222,-0.0120]	-0.145** [-0.253,-0.0376]	-0.125* [-0.222,-0.0272]	-0.115* [-0.220,-0.00946]	-0.134** [-0.232,-0.0355]
Homeopathy (scale: 0–1)	-0.522*** [-0.621,-0.424]	-0.461*** [-0.558,-0.363]	-0.490*** [-0.586,-0.393]	-0.379*** [-0.465,-0.293]	-0.476*** [-0.572,-0.379]	-0.375*** [-0.461,-0.289]
Mainstream medicine (scale: 0–1)	1.446*** [1.280,1.612]	1.424*** [1.260,1.587]	1.454*** [1.290,1.618]	1.043*** [0.894,1.193]	1.335*** [1.170,1.500]	1.034*** [0.884,1.185]
Left = 0 / Right = 1 (scale: 0–1)		-0.870*** [-1.023,-0.718]				-0.0378 [-0.246,0.170]
GAL = 0 / TAN = 1 (scale: 0–1)			-0.625*** [-0.747,-0.503]			-0.0194 [-0.170,0.131]
Voting Intention (Reference: CDU/CSU: Christian democrats)						
SPD (Social democrats)				0.0780** [0.0290,0.127]		0.0509 [-0.0160,0.118]
Greens				0.0708* [0.0158,0.126]		0.0397 [-0.0355,0.115]
FDP (Liberals)				-0.240*** [-0.335,-0.145]		-0.232*** [-0.329,-0.136]
Left-Party (Socialists)				-0.126* [-0.242,-0.0108]		-0.160* [-0.295,-0.0261]
AfD (Populist radical-right)				-1.190*** [-1.316,-1.064]		-1.158*** [-1.287,-1.030]
Other (including Covid-19 protest party)				-0.710*** [-0.837,-0.584]		-0.717*** [-0.847,-0.587]
Solidarity (scale: 0–1)					1.001*** [0.831,1.171]	0.173* [0.00368,0.343]
Constant	1.278*** [0.957,1.600]	1.818*** [1.493,2.142]	1.696*** [1.373,2.020]	2.206*** [1.914,2.499]	0.950*** [0.629,1.271]	2.167*** [1.836,2.498]

Adj. R^2	0.226	0.254	0.246	0.378	0.254	0.379

Note: The models test how the significant effect of Waldorf education in the full model M1.5 emerges from not being significant in the simple model only including Waldorf education, homeopathy and mainstrem medicine (M1a). It shows that Waldorf education is significant if either political factors (left-right, GAL/TAN or voting intention) or the solidarity index are included in the model. Not controlling for these variables as in M1a thus masks the association between Waldorf education and immunization status. N = 7,391. Data weighted by state, age group, gender and voting intention to better represent the German population. All models additionally control for state, gender, age group, education level, household income, religion and the Big 5 personality traits. Exponentiated coefficients; 95% confidence intervals in brackets.* $p < 0.05$, ** $p < 0.01$, *** $p < 0.001$; GAL, green/alternative/liberal; TAN, traditional/authoritarian/nationalist.

Supplementary Table 9: Analysis I crosscheck – Binary logistic regression explaining whether a participant has received at least basic SARS-CoV-2 immunization (odds ratios)

Dependent variable:<br>Basic immunization<br>(0/1 dose = 0;<br>2 or more doses = 1)	M1 log.1<br>Medicine and school attitudes	M1 log.2<br>Religious denomination	M1 log.3<br>Political factors	M1 log.4<br>Psychological factors	M1 log.5<br>Full model
Positive attitudes towards…					
Waldorf education<br>(scale: 0–1)	0.831<br>[0.512,1.349]				0.485*<br>[0.273,0.860]
Homeopathy<br>(scale: 0–1)	0.120***<br>[0.0757,0.189]				0.137***<br>[0.0796,0.237]
Mainstream medicine<br>(scale: 0–1)	84.66***<br>[47.46,151.0]				29.44***<br>[15.07,57.55]
Religious denomination (Reference: no denomination)					
Roman-Catholic		1.986***<br>[1.453,2.715]			1.262<br>[0.863,1.844]
Protestant		1.882***<br>[1.338,2.646]			1.140<br>[0.733,1.771]
Evangelical Free Church		1.549*<br>[1.032,2.325]			1.261<br>[0.720,2.206]
Orthodox		0.321*<br>[0.117,0.883]			0.440<br>[0.0997,1.943]
Jewish		0.409<br>[0.0380,4.396]			1.666<br>[0.151,18.39]
Muslim		1.038<br>[0.253,4.260]			2.102<br>[0.0805,54.88]
Other		0.783<br>[0.408,1.506]			1.128<br>[0.407,3.122]
Left = 0 / Right = 1<br>(scale: 0–1)			0.570<br>[0.243,1.336]		0.551<br>[0.204,1.489]
GAL = 0 / TAN = 1<br>(scale: 0–1)			2.269*<br>[1.162,4.431]		1.328<br>[0.626,2.816]
Voting Intention (Reference: CDU/CSU: Christian democrats)					
SPD (Social democrats)			6.412*<br>[1.458,28.20]		5.677*<br>[1.309,24.62]
Greens			2.293*		2.132

			[1.024,5.133]		[0.939,4.839]
FDP (Liberals)			0.153***		0.174***
			[0.0826,0.283]		[0.0916,0.330]
Left-Party (Socialists)			0.213***		0.246***
			[0.101,0.448]		[0.112,0.539]
AfD (Populist radical-right)			0.0209***		0.0297***
			[0.0120,0.0362]		[0.0163,0.0539]
Other (including Covid-19 protest party)			0.0463***		0.0801***
			[0.0261,0.0821]		[0.0434,0.148]
Solidarity (scale: 0–1)				50.61***	1.264
				[28.31,90.47]	[0.514,3.112]
Agreeableness (Big 5) (scale: 0–1)				0.809	0.908
				[0.450,1.457]	[0.423,1.948]
Conscientiousness (Big 5) (scale: 0–1)				0.818	1.496
				[0.458,1.462]	[0.710,3.152]
Extraversion (Big 5) (scale: 0–1)				1.148	1.541
				[0.721,1.826]	[0.850,2.793]
Neuroticism (Big 5) (scale: 0–1)				3.304***	3.569***
				[1.951,5.593]	[1.801,7.071]
Openness (Big 5) (scale: 0–1)				0.729	0.925
				[0.453,1.174]	[0.507,1.685]
State (Reference: North Rhine-Westphalia)					
Baden-Wuerttemberg	0.790	0.904	0.685	1.036	0.658
	[0.503,1.240]	[0.597,1.369]	[0.422,1.111]	[0.684,1.569]	[0.383,1.131]
Bavaria	0.475***	0.531***	0.572**	0.547***	0.522**
	[0.323,0.699]	[0.379,0.745]	[0.389,0.842]	[0.384,0.778]	[0.336,0.812]
Berlin	0.356***	0.610*	0.510*	0.539*	0.427**
	[0.209,0.606]	[0.376,0.990]	[0.294,0.886]	[0.328,0.885]	[0.230,0.796]
Brandenburg	0.906	1.117	1.444	0.929	1.487
	[0.419,1.959]	[0.567,2.202]	[0.699,2.982]	[0.467,1.845]	[0.630,3.510]
Bremen	3.233	2.313	0.602	2.512	1.285
	[0.783,13.34]	[0.385,13.88]	[0.158,2.288]	[0.392,16.10]	[0.477,3.466]
Hamburg	0.783	1.033	1.047	0.953	0.864
	[0.296,2.068]	[0.445,2.398]	[0.400,2.737]	[0.370,2.450]	[0.281,2.655]

Hesse	0.595*	0.650	0.741	0.651	0.728
	[0.364,0.971]	[0.419,1.010]	[0.448,1.225]	[0.412,1.029]	[0.402,1.319]
Mecklenburg-Vorpommern	0.358**	0.535	0.531	0.388**	0.436
	[0.165,0.775]	[0.274,1.045]	[0.246,1.145]	[0.194,0.779]	[0.171,1.109]
Lower Saxony	1.250	1.671	0.686	1.502	0.700
	[0.737,2.120]	[0.996,2.805]	[0.381,1.234]	[0.884,2.554]	[0.373,1.315]
Rhineland Palatinate	0.582	0.568*	0.564*	0.604	0.499*
	[0.337,1.006]	[0.342,0.944]	[0.322,0.987]	[0.356,1.024]	[0.271,0.920]
Saarland	1.001	0.860	0.868	0.960	0.775
	[0.392,2.558]	[0.293,2.524]	[0.276,2.729]	[0.322,2.863]	[0.265,2.265]
Saxony	0.213***	0.367***	0.387***	0.304***	0.296***
	[0.127,0.357]	[0.237,0.567]	[0.233,0.642]	[0.196,0.473]	[0.160,0.547]
Saxony-Anhalt	0.460	0.676	0.741	0.629	0.776
	[0.204,1.035]	[0.340,1.345]	[0.350,1.569]	[0.314,1.259]	[0.293,2.053]
Schleswig Holstein	0.954	1.313	0.838	1.119	0.631
	[0.445,2.047]	[0.647,2.666]	[0.419,1.676]	[0.562,2.228]	[0.270,1.473]
Thuringia	0.419**	0.716	0.854	0.567	0.522
	[0.222,0.791]	[0.385,1.333]	[0.420,1.738]	[0.299,1.077]	[0.252,1.084]
Foreign country	0.229***	0.304***	0.389**	0.248***	0.327*
	[0.105,0.497]	[0.159,0.582]	[0.193,0.785]	[0.130,0.475]	[0.136,0.786]
Gender (Reference: Male)					
Female	1.547***	0.966	0.643**	0.694**	0.791
	[1.211,1.976]	[0.778,1.200]	[0.474,0.872]	[0.551,0.874]	[0.555,1.127]
Non-binary	0.713	0.905	1.247	0.598	0.571
	[0.222,2.287]	[0.308,2.657]	[0.357,4.352]	[0.176,2.029]	[0.132,2.463]
Age group (Reference: 18-30)					
31-45	0.532**	0.495***	0.567*	0.580**	0.617
	[0.344,0.822]	[0.331,0.742]	[0.361,0.891]	[0.386,0.872]	[0.369,1.032]
46-60	0.500**	0.395***	0.462***	0.493***	0.642
	[0.325,0.768]	[0.266,0.587]	[0.296,0.721]	[0.331,0.734]	[0.388,1.061]
> 60	0.768	0.592*	0.545**	0.577**	0.697
	[0.493,1.198]	[0.395,0.888]	[0.345,0.861]	[0.383,0.870]	[0.415,1.171]
Educational attainment (Reference: Low)					
Middle	0.876	0.983	0.729	0.958	0.775
	[0.534,1.436]	[0.602,1.606]	[0.418,1.272]	[0.577,1.591]	[0.440,1.366]

High	0.816	1.163	0.722	1.020	0.571*
	[0.510,1.307]	[0.725,1.864]	[0.425,1.227]	[0.628,1.656]	[0.330,0.985]
Household income (Reference: I find it very difficult to make ends meet)					
I find it quite difficult to make ends meet	1.495	1.383	1.355	1.582	1.646
	[0.818,2.730]	[0.796,2.400]	[0.716,2.563]	[0.899,2.785]	[0.812,3.338]
I can make ends meet	2.181**	2.117**	1.393	2.315**	1.714
	[1.284,3.707]	[1.281,3.497]	[0.776,2.499]	[1.395,3.841]	[0.924,3.177]
I can live quite comfortably on the income	3.483***	3.979***	2.076*	4.000***	2.314**
	[2.042,5.939]	[2.392,6.621]	[1.149,3.753]	[2.390,6.695]	[1.251,4.279]
I can live very comfortably on the income	2.496**	3.627***	1.513	3.823***	1.397
	[1.373,4.538]	[2.086,6.304]	[0.800,2.858]	[2.184,6.692]	[0.697,2.800]
does not apply (no own household income)	1.163	1.297	0.665	1.102	0.485
	[0.365,3.709]	[0.412,4.084]	[0.155,2.851]	[0.344,3.529]	[0.117,2.011]
Mc Fadden Pseudo R^2	0.248	0.0766	0.357	0.139	0.465
AIC	19936.4	24470.4	17071.1	22815.0	14253.1

Note: The models correspond to those in Supplementary Table 7, except for using a dichotomous outcome variable measuring whether a participant has received at least 2 doses of the vaccine (=basic immunization), or not, instead of the metric one (number of doses). Therefore, a binary logistic regression is modelled. The three main independent variables (Waldorf, homeopathy and mainstream medicine) show similar results (in terms of direction and significance) compared to the multiple linear regression models in Supplementary Table 7. The same is true for most of the other variables that have shown highly significant correlations in the full model M1.5 in Supplementary Table 7, e.g. voting intention, state (Bavaria and Saxony) or the Big 5 trait neuroticism. N = 7,391. Data weighted by state, age group, gender and voting intention to better represent the German population. Exponentiated coefficients; 95% confidence intervals in brackets.* $p < 0.05$, ** $p < 0.01$, *** $p < 0.001$; GAL, green/alternative/liberal; TAN, traditional/authoritarian/nationalist; AIC, Akaike Information Criterion.

Supplementary Table 10: Analysis I – interaction models (unstandardized b-coefficients from multiple linear regression)

Dependent variable:<br># SARS-CoV-2 immunization doses<br>(scale: 0–4)	Interaction 1<br>Attitudes x Age	Interaction 2<br>Attitudes x Gender	Interaction 3<br>Attitudes x Bundesland
Positive attitudes towards…			
Waldorf education<br>(scale: 0–1)	-0.344*** [-0.544,-0.144]	-0.0883 [-0.229,0.0519]	-0.0529 [-0.243,0.137]
Homeopathy<br>(scale: 0–1)	-0.0961 [-0.271,0.0787]	-0.309*** [-0.435,-0.183]	-0.292*** [-0.454,-0.130]
Mainstream medicine<br>(scale: 0–1)	0.505*** [0.209,0.801]	0.969*** [0.747,1.192]	0.748*** [0.453,1.044]
Religious denomination (Reference: no denomination)			
Roman-Catholic	0.0630* [0.00649,0.119]	0.0744* [0.0176,0.131]	0.0724* [0.0154,0.129]
Protestant	0.0301 [-0.0293,0.0895]	0.0313 [-0.0279,0.0906]	0.0262 [-0.0332,0.0856]
Evangelical Free Church	0.0156 [-0.0652,0.0965]	0.0265 [-0.0546,0.108]	0.0189 [-0.0616,0.0994]
Orthodox	-0.364 [-0.766,0.0378]	-0.407 [-0.814,0.000355]	-0.415* [-0.803,-0.0265]
Jewish	0.315 [-0.559,1.189]	0.366 [-0.393,1.126]	0.317 [-0.585,1.219]
Muslim	-0.0647 [-0.409,0.279]	-0.0707 [-0.413,0.272]	-0.0600 [-0.384,0.264]
Other	-0.0817 [-0.292,0.129]	-0.0528 [-0.264,0.159]	-0.0496 [-0.263,0.163]
Left = 0 / Right = 1<br>(scale: 0–1)	-0.0458 [-0.254,0.163]	-0.0265 [-0.236,0.183]	-0.0600 [-0.266,0.146]
GAL = 0 / TAN = 1<br>(scale: 0–1)	-0.0204 [-0.171,0.130]	-0.0187 [-0.169,0.132]	-0.00957 [-0.159,0.140]
Voting Intention (Reference: CDU/CSU: Christian democrats)			
SPD (Social democrats)	0.0555 [-0.0113,0.122]	0.0538 [-0.0131,0.121]	0.0372 [-0.0299,0.104]
Greens	0.0461 [-0.0292,0.121]	0.0377 [-0.0374,0.113]	0.0282 [-0.0470,0.103]
FDP (Liberals)	-0.232*** [-0.329,-0.135]	-0.232*** [-0.329,-0.135]	-0.236*** [-0.332,-0.139]
Left-Party (Socialists)	-0.160* [-0.294,-0.0260]	-0.156* [-0.290,-0.0209]	-0.171* [-0.304,-0.0370]
AfD (Populist radical-right)	-1.148*** [-1.276,-1.019]	-1.165*** [-1.294,-1.036]	-1.168*** [-1.296,-1.040]
Other (including Covid-19 protest party)	-0.707*** [-0.836,-0.578]	-0.713*** [-0.843,-0.584]	-0.713*** [-0.843,-0.583]
Solidarity<br>(scale: 0–1)	0.176* [0.00691,0.346]	0.169 [-0.000918,0.339]	0.178* [0.00789,0.349]
Agreeableness (Big 5)<br>(scale: 0–1)	-0.143* [-0.267,-0.0203]	-0.148* [-0.271,-0.0249]	-0.131* [-0.254,-0.00743]

Conscientiousness (Big 5) (scale: 0–1)	0.0354 [-0.0863,0.157]	0.0325 [-0.0902,0.155]	0.0208 [-0.102,0.144]
Extraversion (Big 5) (scale: 0–1)	0.100* [0.00418,0.196]	0.0963 [-0.000601,0.193]	0.101* [0.00412,0.197]
Neuroticism (Big 5) (scale: 0–1)	0.249*** [0.141,0.357]	0.248*** [0.140,0.356]	0.249*** [0.141,0.356]
Openness (Big 5) (scale: 0–1)	-0.0701 [-0.170,0.0295]	-0.0629 [-0.163,0.0370]	-0.0550 [-0.155,0.0445]
State (Reference: North Rhine-Westphalia)			
Baden-Wuerttemberg	-0.151*** [-0.220,-0.0821]	-0.146*** [-0.215,-0.0769]	-0.264 [-0.727,0.199]
Bavaria	-0.156*** [-0.229,-0.0831]	-0.149*** [-0.222,-0.0762]	-0.379 [-0.800,0.0415]
Berlin	-0.198** [-0.321,-0.0749]	-0.189** [-0.313,-0.0649]	-0.224 [-0.959,0.511]
Brandenburg	0.0573 [-0.0844,0.199]	0.0660 [-0.0769,0.209]	0.0806 [-0.752,0.913]
Bremen	0.162 [-0.0166,0.341]	0.160 [-0.0177,0.337]	-0.612 [-1.676,0.453]
Hamburg	-0.0172 [-0.177,0.143]	-0.000575 [-0.159,0.157]	-0.271 [-1.786,1.243]
Hesse	-0.0987* [-0.196,-0.00117]	-0.0963 [-0.194,0.00137]	-0.256 [-0.816,0.303]
Mecklenburg-Vorpommern	-0.216* [-0.427,-0.00517]	-0.216* [-0.426,-0.00545]	-1.018 [-2.245,0.209]
Lower Saxony	-0.0217 [-0.0981,0.0546]	-0.0114 [-0.0886,0.0658]	-0.285 [-0.751,0.180]
Rhineland Palatinate	-0.0989 [-0.210,0.0127]	-0.106 [-0.219,0.00695]	-0.204 [-0.888,0.479]
Saarland	-0.0612 [-0.225,0.103]	-0.0594 [-0.219,0.100]	-0.345 [-1.169,0.479]
Saxony	-0.343*** [-0.470,-0.217]	-0.330*** [-0.457,-0.203]	-0.460 [-1.196,0.276]
Saxony-Anhalt	-0.0728 [-0.263,0.118]	-0.0644 [-0.254,0.126]	-0.403 [-1.500,0.695]
Schleswig Holstein	0.0355 [-0.0691,0.140]	0.0509 [-0.0543,0.156]	-0.0307 [-0.609,0.548]
Thuringia	-0.124 [-0.275,0.0276]	-0.111 [-0.262,0.0408]	-0.0646 [-1.096,0.967]
Foreign country	-0.262** [-0.458,-0.0668]	-0.254* [-0.449,-0.0596]	-0.809 [-1.994,0.375]
Gender (Reference: Male)			
Female	-0.0323 [-0.0847,0.0201]	-0.0355 [-0.313,0.242]	-0.0292 [-0.0815,0.0230]
Non-binary	0.0312 [-0.262,0.324]	0.378 [-2.128,2.884]	0.0113 [-0.282,0.304]
Age group (Reference: 18-30)			
31-45	-0.424* [-0.826,-0.0219]	-0.0287 [-0.0949,0.0374]	-0.0321 [-0.0978,0.0335]

46-60	-0.581** [-0.976,-0.186]	0.0576 [-0.0104,0.126]	0.0420 [-0.0255,0.109]
> 60	-0.0730 [-0.451,0.305]	0.349*** [0.280,0.417]	0.342*** [0.274,0.411]
Educational attainment (Reference: Low)			
Middle	-0.0799 [-0.201,0.0413]	-0.0725 [-0.194,0.0492]	-0.0744 [-0.195,0.0465]
High	-0.116* [-0.231,-0.00102]	-0.111 [-0.226,0.00447]	-0.115* [-0.230,-0.000139]
Household income (Reference: I find it very difficult to make ends meet)			
I find it quite difficult to make ends meet	0.108 [-0.0979,0.314]	0.118 [-0.0919,0.327]	0.126 [-0.0818,0.334]
I can make ends meet	0.145 [-0.0407,0.331]	0.166 [-0.0234,0.355]	0.166 [-0.0224,0.354]
I can live quite comfortably on the income	0.176 [-0.00849,0.360]	0.192* [0.00460,0.380]	0.200* [0.0139,0.387]
I can live very comfortably on the income	0.0854 [-0.104,0.275]	0.105 [-0.0874,0.297]	0.108 [-0.0831,0.299]
does not apply (no own household income)	0.0657 [-0.197,0.328]	0.0618 [-0.208,0.331]	0.0477 [-0.217,0.312]
Interactions			
18-30 # Mainstream medicine	Reference		
31-45 # Mainstream medicine	0.491* [0.0685,0.913]		
46-60 # Mainstream medicine	0.813*** [0.394,1.233]		
> 60 # Mainstream medicine	0.610** [0.208,1.011]		
18-30 # Homeopathy	Reference		
31-45 # Homeopathy	-0.237 [-0.483,0.00879]		
46-60 # Homeopathy	-0.289* [-0.523,-0.0544]		
> 60 # Homeopathy	-0.433*** [-0.664,-0.203]		
18-30 # Waldorf education	Reference		
31-45 # Waldorf education	0.222 [-0.0548,0.499]		
46-60 # Waldorf education	0.251 [-0.0221,0.525]		
> 60 # Waldorf education	0.259 [-0.00458,0.523]		
Male # Mainstream medicine		Reference	
Female # Mainstream medicine		0.116 [-0.180,0.413]	

Non-binary # Mainstream medicine	-0.0133<br>[-3.006,2.980]	
Male # Homeopathy	Reference	
Female # Homeopathy	-0.119<br>[-0.287,0.0477]	
Non-binary # Homeopathy	-0.689<br>[-2.018,0.639]	
Male # Waldorf education	Reference	
Female # Waldorf education	-0.0817<br>[-0.275,0.112]	
Non-binary # Waldorf education	-0.334<br>[-1.252,0.583]	
Baden-Wuerttemberg # Mainstream medicine		0.204<br>[-0.288,0.695]
Bavaria # Mainstream medicine		0.570*<br>[0.118,1.023]
Berlin # Mainstream medicine		0.364<br>[-0.383,1.112]
Brandenburg # Mainstream medicine		0.0578<br>[-0.741,0.857]
Bremen # Mainstream medicine		0.832<br>[-0.352,2.016]
Hamburg # Mainstream medicine		0.491<br>[-1.180,2.161]
Hesse # Mainstream medicine		0.348<br>[-0.245,0.940]
Mecklenburg-Vorpommern # Mainstream medicine		0.822<br>[-0.469,2.112]
Lower Saxony # Mainstream medicine		0.176<br>[-0.339,0.691]
North Rhine-Westphalia # Mainstream medicine		Reference
Rhineland Palatinate # Mainstream medicine		0.382<br>[-0.387,1.150]
Saarland # Mainstream medicine		0.392<br>[-0.572,1.355]
Saxony # Mainstream medicine		0.369<br>[-0.439,1.178]
Saxony-Anhalt # Mainstream medicine		0.183<br>[-1.002,1.368]
Schleswig Holstein # Mainstream medicine		-0.0218<br>[-0.643,0.599]
Thuringia # Mainstream medicine		0.376<br>[-0.777,1.530]
Foreign country # Mainstream medicine		0.674<br>[-0.603,1.950]

Baden-Wuerttemberg # Homeopathy	-0.0206<br>[-0.286,0.244]
Bavaria # Homeopathy	-0.112<br>[-0.373,0.149]
Berlin # Homeopathy	-0.616**<br>[-1.073,-0.158]
Brandenburg # Homeopathy	0.290<br>[-0.195,0.776]
Bremen # Homeopathy	0.416<br>[-0.167,0.999]
Hamburg # Homeopathy	0.0214<br>[-0.562,0.605]
Hesse # Homeopathy	-0.0466<br>[-0.378,0.285]
Mecklenburg-Vorpommern # Homeopathy	-0.103<br>[-1.092,0.886]
Lower Saxony # Homeopathy	0.0670<br>[-0.208,0.342]
North Rhine-Westphalia # Homeopathy	Reference
Rhineland Palatinate # Homeopathy	-0.465*<br>[-0.906,-0.0249]
Saarland # Homeopathy	-0.112<br>[-0.503,0.278]
Saxony # Homeopathy	-0.352<br>[-0.798,0.0942]
Saxony-Anhalt # Homeopathy	0.505<br>[-0.295,1.306]
Schleswig Holstein # Homeopathy	-0.0762<br>[-0.481,0.329]
Thuringia # Homeopathy	-0.619<br>[-1.263,0.0255]
Foreign country # Homeopathy	-0.0781<br>[-0.926,0.770]
Baden-Wuerttemberg # Waldorf education	-0.0867<br>[-0.403,0.229]
Bavaria # Waldorf education	-0.396**<br>[-0.694,-0.0974]
Berlin # Waldorf education	-0.0443<br>[-0.542,0.453]
Brandenburg # Waldorf education	-0.449<br>[-1.015,0.116]
Bremen # Waldorf education	-0.214<br>[-1.023,0.596]
Hamburg # Waldorf education	-0.301<br>[-0.794,0.192]
Hesse # Waldorf education	-0.231<br>[-0.677,0.215]

Mecklenburg-Vorpommern # Waldorf education			0.516<br>[-0.428,1.459]
Lower Saxony # Waldorf education			0.282<br>-0.0867
North Rhine-Westphalia # Waldorf education			Reference
Rhineland Palatinate # Waldorf education			0.0474<br>[-0.400,0.494]
Saarland # Waldorf education			0.0542<br>[-0.465,0.573]
Saxony # Waldorf education			-0.0719<br>[-0.628,0.484]
Saxony-Anhalt # Waldorf education			-0.00985<br>[-0.836,0.816]
Schleswig Holstein # Waldorf education			0.299<br>[-0.156,0.755]
Thuringia # Waldorf education			-0.182<br>[-0.980,0.617]
Foreign country # Waldorf education			0.156<br>[-0.836,1.147]
Constant	2.594***<br>[2.192,2.996]	2.165***<br>[1.801,2.529]	2.322***<br>[1.916,2.727]
Adj. R^2	0.384	0.379	0.386

Note: All interaction models are based on the full model M1.5 from Supplementary Table 7. N = 7,391. Data weighted by state, age group, gender and voting intention to better represent the German population. Exponentiated coefficients; 95% confidence intervals in brackets.* p < 0.05, ** p < 0.01, *** p < 0.001; GAL, green/alternative/liberal; TAN, traditional/authoritarian/nationalist.

Supplementary Table 11: Analysis II – Impact on the self-perceived relative importance of six concrete reasons for SARS-CoV-2 immunization decisions (unstandardized b-coefficients from multiple linear regression)

	Dependent variables: Drivers of SARS-CoV-2 immunization decisions (scale: 0–1; 0 = not at all important; 1 = very important)					
	M2a<br>Protecting self	M2b<br>Protecting others	M2c<br>Medical advice / recommendation by public health authorities (e.g. STIKO)	M2d<br>Participation in public events	M2e<br>Vocational mandates	M2f<br>Peer pressure
Positive attitudes towards…						
Waldorf education	-0.0372*	-0.0117	0.0281	0.0685**	0.0542*	0.0177
(scale: 0–1)	[-0.0683,-0.00609]	[-0.0458,0.0224]	[-0.0123,0.0685]	[0.0233,0.114]	[0.00358,0.105]	[-0.0213,0.0566]
Homeopathy	-0.0464***	-0.0563***	-0.0751***	0.0627**	0.0786***	0.0205
(scale: 0–1)	[-0.0735,-0.0193]	[-0.0863,-0.0262]	[-0.111,-0.0395]	[0.0235,0.102]	[0.0336,0.124]	[-0.0139,0.0549]
Mainstream medicine	0.272***	0.161***	0.391***	-0.0368	-0.0346	-0.0219
(scale: 0–1)	[0.223,0.321]	[0.112,0.211]	[0.338,0.444]	[-0.0965,0.0228]	[-0.103,0.0342]	[-0.0754,0.0317]
Religious denomination (Reference: no denomination)						
Roman-Catholic	0.0184	0.0365***	0.0222	-0.0133	0.0327*	0.0147
	[-0.0000375,0.0369]	[0.0158,0.0572]	[-0.00309,0.0474]	[-0.0407,0.0142]	[0.00174,0.0637]	[-0.00886,0.0383]
Protestant	0.00669	0.0237*	0.0389**	-0.0445**	0.0200	0.0241
	[-0.0122,0.0255]	[0.00262,0.0447]	[0.0125,0.0652]	[-0.0726,-0.0164]	[-0.0122,0.0522]	[-0.000752,0.0489]
Evangel. Free Church	0.00948	0.0270	0.0443*	0.00473	0.0671**	0.0363*
	[-0.0171,0.0361]	[-0.000698,0.0548]	[0.0102,0.0785]	[-0.0317,0.0412]	[0.0243,0.110]	[0.00294,0.0697]
Orthodox	-0.0607	0.0584	0.0642	0.0916	-0.0685	0.0296
	[-0.245,0.123]	[-0.0301,0.147]	[-0.0633,0.192]	[-0.0639,0.247]	[-0.241,0.105]	[-0.207,0.266]
Jewish	-0.233	-0.0610	-0.0547	-0.0499	0.235	0.0241
	[-0.520,0.0542]	[-0.252,0.130]	[-0.355,0.246]	[-0.295,0.195]	[-0.112,0.582]	[-0.181,0.229]
Muslim	0.0256	0.00813	0.0853	-0.0686	0.222*	0.0616
	[-0.0566,0.108]	[-0.121,0.137]	[-0.00909,0.180]	[-0.220,0.0833]	[0.0411,0.402]	[-0.0797,0.203]
Other	0.0213	0.0249	-0.00711	-0.0476	0.0358	-0.0127
	[-0.0406,0.0832]	[-0.0418,0.0916]	[-0.0856,0.0714]	[-0.129,0.0340]	[-0.0699,0.141]	[-0.0752,0.0498]
Left = 0 / Right = 1	-0.0288	-0.0861*	-0.0357	0.0611	0.0981*	0.0355
(scale: 0–1)	[-0.0935,0.0360]	[-0.157,-0.0153]	[-0.114,0.0422]	[-0.0226,0.145]	[0.00258,0.194]	[-0.0381,0.109]
GAL = 0 / TAN = 1	-0.0313	-0.120***	-0.0769*	0.108***	0.0124	-0.0128

(scale: 0–1)	[-0.0789,0.0163]	[-0.171,-0.0684]	[-0.136,-0.0179]	[0.0450,0.171]	[-0.0581,0.0828]	[-0.0688,0.0431]
Voting Intention (Reference: CDU/CSU: Christian democrats)						
SPD (Social democrats)	0.00541	-0.0179	-0.00662	-0.00297	-0.00809	-0.00631
	[-0.0179,0.0287]	[-0.0443,0.00855]	[-0.0390,0.0257]	[-0.0387,0.0328]	[-0.0492,0.0330]	[-0.0376,0.0250]
Greens	0.0136	-0.0243	-0.0315	-0.0149	-0.0556*	-0.0113
	[-0.0106,0.0378]	[-0.0516,0.00308]	[-0.0655,0.00239]	[-0.0518,0.0220]	[-0.0983,-0.0129]	[-0.0440,0.0213]
FDP (Liberals)	-0.0727***	-0.0981***	-0.111***	0.0633**	-0.0310	-0.0173
	[-0.106,-0.0397]	[-0.133,-0.0629]	[-0.150,-0.0716]	[0.0232,0.103]	[-0.0798,0.0179]	[-0.0539,0.0192]
Left-Party (Socialists)	-0.0571*	-0.0683**	-0.126***	-0.0130	-0.0676*	-0.0458
	[-0.101,-0.0134]	[-0.113,-0.0233]	[-0.178,-0.0738]	[-0.0695,0.0436]	[-0.133,-0.00261]	[-0.0953,0.00377]
AfD (Populist radical-right)	-0.319***	-0.321***	-0.257***	-0.0165	-0.0568*	-0.0646***
	[-0.364,-0.273]	[-0.366,-0.276]	[-0.301,-0.213]	[-0.0641,0.0312]	[-0.110,-0.00383]	[-0.103,-0.0264]
Other (including Covid-19 protest party)	-0.107***	-0.133***	-0.125***	0.0237	-0.0152	-0.00883
	[-0.149,-0.0652]	[-0.174,-0.0914]	[-0.168,-0.0825]	[-0.0241,0.0714]	[-0.0701,0.0396]	[-0.0495,0.0318]
Solidarity (scale: 0–1)	0.137***	0.357***	0.163***	-0.131***	-0.0548	-0.0369
	[0.0813,0.192]	[0.296,0.418]	[0.0916,0.234]	[-0.205,-0.0574]	[-0.138,0.0282]	[-0.100,0.0265]
Agreeableness (Big 5) (scale: 0–1)	-0.0672***	0.0353	0.0175	0.0233	0.0535	0.0722**
	[-0.107,-0.0273]	[-0.00818,0.0788]	[-0.0350,0.0700]	[-0.0341,0.0807]	[-0.0114,0.118]	[0.0235,0.121]
Conscientiousness (Big 5) (scale: 0–1)	0.0413*	0.0978***	0.0485	-0.0551*	0.110***	-0.0762**
	[0.00296,0.0796]	[0.0566,0.139]	[-0.00153,0.0986]	[-0.110,-0.000675]	[0.0485,0.172]	[-0.125,-0.0279]
Extraversion (Big 5) (scale: 0–1)	0.0246	0.0327	0.0425*	0.157***	0.0543*	0.0925***
	[-0.00714,0.0564]	[-0.00110,0.0665]	[0.000188,0.0847]	[0.114,0.201]	[0.00435,0.104]	[0.0546,0.130]
Neuroticism (Big 5) (scale: 0–1)	0.0322	0.0572**	0.0831***	0.0103	-0.0355	0.0518*
	[-0.00230,0.0667]	[0.0203,0.0941]	[0.0370,0.129]	[-0.0393,0.0598]	[-0.0911,0.0202]	[0.00818,0.0953]
Openness (Big 5) (scale: 0–1)	-0.0105	0.0271	-0.00657	-0.0191	0.0518*	-0.0135
	[-0.0427,0.0218]	[-0.00778,0.0621]	[-0.0486,0.0355]	[-0.0649,0.0266]	[0.000195,0.103]	[-0.0522,0.0251]
State (Reference: North Rhine-Westphalia)						
Baden-Wuerttemberg	-0.0329**	-0.00939	-0.0301	0.0338	-0.0179	-0.00947
	[-0.0563,-0.00941]	[-0.0350,0.0162]	[-0.0614,0.00120]	[-0.000527,0.0682]	[-0.0573,0.0214]	[-0.0402,0.0212]
Bavaria	-0.0331**	-0.0193	-0.0245	0.0162	-0.00888	-0.01000
	[-0.0561,-0.0100]	[-0.0447,0.00621]	[-0.0548,0.00582]	[-0.0162,0.0485]	[-0.0459,0.0281]	[-0.0389,0.0189]

Berlin	-0.00676	0.00656	0.0101	0.0659*	-0.0160	0.0276
	[-0.0457,0.0322]	[-0.0330,0.0461]	[-0.0418,0.0621]	[0.0152,0.117]	[-0.0769,0.0448]	[-0.0207,0.0759]
Brandenburg	-0.00536	0.00668	0.00197	0.0162	0.00244	-0.0386
	[-0.0495,0.0388]	[-0.0363,0.0497]	[-0.0523,0.0562]	[-0.0429,0.0752]	[-0.0670,0.0719]	[-0.0860,0.00883]
Bremen	0.0485*	-0.00878	-0.0271	0.00613	0.00263	-0.0770*
	[0.00160,0.0953]	[-0.0780,0.0605]	[-0.119,0.0642]	[-0.0827,0.0950]	[-0.118,0.123]	[-0.151,-0.00266]
Hamburg	0.000768	0.00240	0.0448	0.0427	-0.0556	0.0422
	[-0.0460,0.0475]	[-0.0507,0.0555]	[-0.0205,0.110]	[-0.0276,0.113]	[-0.133,0.0221]	[-0.0207,0.105]
Hesse	-0.00754	-0.0139	0.0202	0.0277	0.00288	0.0155
	[-0.0369,0.0218]	[-0.0477,0.0200]	[-0.0177,0.0582]	[-0.0154,0.0709]	[-0.0455,0.0513]	[-0.0232,0.0542]
Mecklenburg-Vorpommern	-0.0328	-0.0267	0.0153	0.0266	0.0430	0.0641
	[-0.0922,0.0267]	[-0.0820,0.0285]	[-0.0582,0.0887]	[-0.0479,0.101]	[-0.0502,0.136]	[-0.00878,0.137]
Lower Saxony	-0.0148	-0.0134	-0.0123	-0.0166	0.0189	-0.00323
	[-0.0400,0.0104]	[-0.0426,0.0158]	[-0.0471,0.0226]	[-0.0555,0.0223]	[-0.0260,0.0638]	[-0.0374,0.0309]
Rhineland Palatinate	0.00592	-0.0194	-0.00832	0.0224	-0.0383	-0.0373
	[-0.0295,0.0414]	[-0.0599,0.0210]	[-0.0576,0.0410]	[-0.0269,0.0718]	[-0.0948,0.0182]	[-0.0774,0.00275]
Saarland	-0.000631	0.00455	-0.00242	-0.000324	-0.0616	-0.0809*
	[-0.0631,0.0618]	[-0.0496,0.0587]	[-0.0791,0.0743]	[-0.0896,0.0890]	[-0.160,0.0366]	[-0.155,-0.00627]
Saxony	-0.0411	-0.0229	-0.0298	0.0227	0.0273	-0.0363
	[-0.0827,0.000431]	[-0.0642,0.0184]	[-0.0783,0.0188]	[-0.0305,0.0759]	[-0.0314,0.0860]	[-0.0799,0.00718]
Saxony-Anhalt	0.0297	-0.00458	-0.0443	-0.0275	0.0103	-0.0368
	[-0.0203,0.0796]	[-0.0621,0.0530]	[-0.115,0.0266]	[-0.0996,0.0447]	[-0.0724,0.0930]	[-0.0937,0.0201]
Schleswig Holstein	0.0160	-0.00613	-0.0618*	-0.0245	0.0179	-0.00522
	[-0.0157,0.0477]	[-0.0470,0.0348]	[-0.116,-0.00767]	[-0.0814,0.0325]	[-0.0473,0.0832]	[-0.0514,0.0410]
Thuringia	-0.00942	0.00151	-0.0218	-0.0162	-0.0751*	-0.0161
	[-0.0635,0.0446]	[-0.0516,0.0546]	[-0.0824,0.0388]	[-0.0815,0.0491]	[-0.144,-0.00665]	[-0.0693,0.0371]
Foreign country	-0.0972**	-0.0742	-0.0860	0.00489	-0.0943*	-0.0305
	[-0.167,-0.0272]	[-0.149,0.000480]	[-0.173,0.000955]	[-0.0697,0.0795]	[-0.181,-0.00763]	[-0.0913,0.0304]
Gender (Reference: Male)						
Female	0.00227	0.0215*	0.0406***	-0.0320**	-0.00146	-0.00930
	[-0.0140,0.0185]	[0.00344,0.0396]	[0.0187,0.0624]	[-0.0563,-0.00782]	[-0.0287,0.0258]	[-0.0300,0.0114]
Non-binary	-0.0598	0.0526	-0.0108	-0.0753	-0.0356	0.0259
	[-0.153,0.0338]	[-0.0190,0.124]	[-0.102,0.0798]	[-0.188,0.0371]	[-0.176,0.105]	[-0.0720,0.124]

Age group (Reference: 18-30)						
31-45	0.0152	-0.00284	-0.00186	-0.0975***	0.0665***	-0.0511***
	[-0.00904,0.0395]	[-0.0276,0.0219]	[-0.0308,0.0271]	[-0.129,-0.0661]	[0.0315,0.102]	[-0.0791,-0.0230]
46-60	0.0483***	0.00432	-0.0121	-0.0928***	0.101***	-0.0751***
	[0.0250,0.0716]	[-0.0197,0.0284]	[-0.0412,0.0171]	[-0.124,-0.0616]	[0.0654,0.137]	[-0.102,-0.0478]
> 60	0.0795***	0.0184	0.0370*	-0.0589***	0.0279	0.000861
	[0.0563,0.103]	[-0.00560,0.0425]	[0.00784,0.0661]	[-0.0900,-0.0277]	[-0.00775,0.0635]	[-0.0273,0.0290]
Educational attainment (Reference: Low)						
Middle	-0.0348	-0.0507*	-0.0476	0.0208	-0.0413	-0.0592*
	[-0.0705,0.00103]	[-0.0906,-0.0107]	[-0.0990,0.00370]	[-0.0328,0.0744]	[-0.105,0.0222]	[-0.110,-0.00828]
High	-0.0506**	-0.0695***	-0.0284	0.0111	-0.0627*	-0.0403
	[-0.0849,-0.0163]	[-0.108,-0.0313]	[-0.0780,0.0212]	[-0.0402,0.0624]	[-0.123,-0.00227]	[-0.0895,0.00883]
Household income (Reference: I find it very difficult to make ends meet)						
I find it quite difficult to make ends meet	0.00219	0.00923	-0.00779	0.0670	0.0662	-0.000186
	[-0.0629,0.0673]	[-0.0501,0.0685]	[-0.0820,0.0664]	[-0.0158,0.150]	[-0.0246,0.157]	[-0.0671,0.0668]
I can make ends meet	0.00773	0.0108	0.000922	0.101**	0.0598	0.0246
	[-0.0509,0.0664]	[-0.0410,0.0626]	[-0.0664,0.0682]	[0.0265,0.175]	[-0.0236,0.143]	[-0.0369,0.0862]
I can live quite comfortably on the income	0.0254	0.0178	0.0191	0.130***	0.0534	0.0247
	[-0.0327,0.0835]	[-0.0336,0.0692]	[-0.0477,0.0859]	[0.0562,0.204]	[-0.0295,0.136]	[-0.0364,0.0858]
I can live very comfortably on the income	0.0120	0.0161	0.00189	0.112**	0.00880	0.0224
	[-0.0477,0.0717]	[-0.0373,0.0696]	[-0.0677,0.0714]	[0.0360,0.189]	[-0.0770,0.0946]	[-0.0414,0.0863]
does not apply (no own household income)	0.0301	-0.0424	0.0179	0.180**	0.0182	0.125*
	[-0.0562,0.117]	[-0.140,0.0552]	[-0.0997,0.136]	[0.0647,0.295]	[-0.112,0.148]	[0.0135,0.236]
Constant	0.620***	0.470***	0.209**	0.474***	0.167*	0.265***
	[0.519,0.721]	[0.361,0.578]	[0.0796,0.339]	[0.338,0.609]	[0.00929,0.325]	[0.141,0.388]
Adj. R^2	0.239	0.288	0.179	0.0695	0.0564	0.0338

Note: N = 6,657. Data weighted by state, age group, gender and voting intention to better represent the German population.
Exponentiated coefficients; 95% confidence intervals in brackets.* $p < 0.05$, ** $p < 0.01$, *** $p < 0.001$; GAL, green/alternative/liberal; TAN, traditional/authoritarian/nationalist.

Supplementary Table 12: Analysis II crosscheck – Ordinal logistic regression explaining the level of self-perceived relative importance of six concrete reasons for SARS-CoV-2 immunization decisions (odds ratios)

	M2a_or<br>Protecting self	M2b_or<br>Protecting others	M2c_or<br>Medical advice/ recommendation by public health authorities (e.g. STIKO)	M2d_or<br>Participation in public events	M2e_or<br>Vocational mandates	M2f_or<br>Peer pressure
Dependent variables: Drivers of SARS-CoV-2 immunization decisions (levels: 0 = Highly unimportant, 0.25 = Mostly unimportant, 0.5 = Semi-important, 0.75 = Mostly important, 1 = Highly important)						
Positive attitudes towards…						
Waldorf education	0.750	0.918	1.158	1.414**	1.321*	1.146
(scale: 0–1)	[0.555,1.013]	[0.696,1.210]	[0.897,1.496]	[1.088,1.837]	[1.020,1.711]	[0.878,1.496]
Homeopathy	0.622***	0.658***	0.603***	1.472***	1.526***	1.119
(scale: 0–1)	[0.477,0.811]	[0.517,0.837]	[0.482,0.754]	[1.173,1.847]	[1.214,1.917]	[0.886,1.411]
Mainstream medicine	11.39***	3.850***	12.21***	0.825	0.844	0.881
(scale: 0–1)	[7.538,17.22]	[2.638,5.620]	[8.644,17.24]	[0.578,1.176]	[0.591,1.205]	[0.610,1.272]
Religious denomination (Reference: no denomination)						
Roman-Catholic	1.150	1.312**	1.154	0.920	1.189*	1.079
	[0.953,1.388]	[1.104,1.558]	[0.989,1.348]	[0.788,1.074]	[1.022,1.383]	[0.921,1.265]
Protestant	0.998	1.200*	1.304**	0.775**	1.107	1.167
	[0.819,1.216]	[1.005,1.432]	[1.103,1.541]	[0.664,0.906]	[0.942,1.301]	[0.991,1.374]
Evangel. Free Church	1.054	1.196	1.335**	1.027	1.387**	1.210
	[0.823,1.350]	[0.959,1.491]	[1.078,1.653]	[0.837,1.261]	[1.122,1.714]	[0.969,1.511]
Orthodox	0.735	1.287	1.353	1.430	0.775	0.771
	[0.226,2.389]	[0.489,3.387]	[0.683,2.677]	[0.593,3.446]	[0.326,1.839]	[0.0948,6.274]
Jewish	0.246	0.667	0.709	0.763	3.683	1.202
	[0.0265,2.288]	[0.153,2.905]	[0.0483,10.40]	[0.225,2.587]	[0.658,20.63]	[0.325,4.443]
Muslim	0.887	1.273	1.581	0.710	3.105*	1.370
	[0.374,2.101]	[0.446,3.635]	[0.872,2.865]	[0.306,1.644]	[1.161,8.307]	[0.597,3.143]
Other	1.240	1.276	0.950	0.789	1.212	0.923
	[0.689,2.231]	[0.762,2.139]	[0.581,1.553]	[0.497,1.253]	[0.702,2.092]	[0.579,1.470]
Left = 0/Right = 1	0.634	0.475**	0.826	1.391	1.740*	1.440
(scale: 0–1)	[0.351,1.142]	[0.271,0.831]	[0.507,1.345]	[0.850,2.276]	[1.062,2.851]	[0.862,2.407]
GAL = 0/TAN = 1	1.014	0.394***	0.595**	1.856***	1.050	0.831
(scale: 0–1)	[0.652,1.576]	[0.263,0.590]	[0.412,0.858]	[1.289,2.672]	[0.733,1.502]	[0.566,1.222]
Voting Intention (Reference: CDU/CSU: Christian democrats)						
SPD (Social democrats)	1.093	0.867	0.956	0.976	1.000	0.951

	[0.854,1.399]	[0.697,1.078]	[0.781,1.170]	[0.797,1.194]	[0.817,1.225]	[0.769,1.175]
Greens	1.154	0.837	0.816	0.908	0.789*	0.970
	[0.897,1.485]	[0.665,1.054]	[0.660,1.009]	[0.739,1.117]	[0.638,0.976]	[0.779,1.209]
FDP (Liberals)	0.569***	0.505***	0.524***	1.476**	0.873	0.879
	[0.437,0.742]	[0.397,0.643]	[0.413,0.665]	[1.160,1.879]	[0.683,1.116]	[0.687,1.124]
Left-Party (Socialists)	0.593**	0.629*	0.458***	0.914	0.709	0.711
	[0.405,0.867]	[0.439,0.901]	[0.335,0.628]	[0.667,1.252]	[0.501,1.003]	[0.505,1.001]
AfD (Populist radical-right)	0.135***	0.157***	0.211***	0.978	0.735*	0.616***
	[0.100,0.182]	[0.116,0.213]	[0.157,0.283]	[0.734,1.304]	[0.560,0.964]	[0.463,0.819]
Other (including Covid-19 protest party)	0.540***	0.424***	0.484***	1.125	0.937	0.989
	[0.388,0.753]	[0.315,0.572]	[0.374,0.625]	[0.853,1.483]	[0.710,1.236]	[0.753,1.299]
Solidarity (scale: 0–1)	3.302***	17.30***	2.901***	0.435***	0.791	0.734
	[1.978,5.510]	[10.73,27.89]	[1.845,4.560]	[0.284,0.669]	[0.515,1.213]	[0.474,1.136]
Agreeableness (Big 5) (scale: 0–1)	0.511***	1.342	1.133	1.126	1.259	1.740**
	[0.344,0.760]	[0.946,1.904]	[0.816,1.574]	[0.809,1.566]	[0.902,1.758]	[1.246,2.431]
Conscientiousness (Big 5) (scale: 0–1)	1.696**	2.659***	1.389*	0.742	1.642**	0.514***
	[1.176,2.445]	[1.897,3.727]	[1.012,1.907]	[0.543,1.013]	[1.204,2.238]	[0.371,0.713]
Extraversion (Big 5) (scale: 0–1)	1.262	1.363*	1.338*	2.406***	1.319*	1.900***
	[0.927,1.717]	[1.039,1.788]	[1.026,1.744]	[1.875,3.088]	[1.021,1.705]	[1.469,2.458]
Neuroticism (Big 5) (scale: 0–1)	1.189	1.404*	1.658***	1.047	0.805	1.536**
	[0.846,1.672]	[1.036,1.901]	[1.240,2.216]	[0.789,1.388]	[0.604,1.072]	[1.143,2.063]
Openness (Big 5) (scale: 0–1)	1.054	1.361*	0.934	0.932	1.309*	0.904
	[0.769,1.444]	[1.022,1.813]	[0.718,1.215]	[0.716,1.215]	[1.005,1.706]	[0.694,1.177]
State (Reference: North Rhine-Westphalia)						
Baden-Wuerttemberg	0.720**	0.879	0.822*	1.185	0.947	0.957
	[0.567,0.915]	[0.708,1.091]	[0.679,0.996]	[0.975,1.440]	[0.780,1.149]	[0.784,1.170]
Bavaria	0.716**	0.845	0.843	1.065	0.977	0.914
	[0.572,0.896]	[0.684,1.043]	[0.698,1.019]	[0.891,1.275]	[0.813,1.174]	[0.752,1.111]
Berlin	0.882	1.042	1.102	1.427*	0.943	1.166
	[0.611,1.272]	[0.749,1.449]	[0.797,1.523]	[1.064,1.914]	[0.688,1.292]	[0.845,1.607]
	0.853	0.961	0.984	1.075	1.018	0.753

Brandenburg						
	[0.569,1.278]	[0.682,1.352]	[0.701,1.381]	[0.776,1.488]	[0.718,1.443]	[0.529,1.072]
Bremen	2.338	0.812	0.824	0.951	1.125	0.656
	[0.934,5.855]	[0.456,1.448]	[0.482,1.408]	[0.591,1.532]	[0.605,2.093]	[0.384,1.122]
Hamburg	0.945	0.975	1.274	1.247	0.740	1.242
	[0.596,1.499]	[0.635,1.498]	[0.816,1.988]	[0.827,1.880]	[0.495,1.105]	[0.819,1.883]
Hesse	0.925	0.911	1.130	1.156	1.041	1.034
	[0.696,1.229]	[0.694,1.197]	[0.889,1.438]	[0.903,1.481]	[0.819,1.323]	[0.799,1.338]
Mecklenburg-Vorpommern	0.726	0.811	1.119	1.125	1.223	1.482
	[0.450,1.172]	[0.528,1.246]	[0.708,1.769]	[0.734,1.724]	[0.752,1.991]	[0.942,2.331]
Lower Saxony	0.913	0.906	0.913	0.906	1.127	0.980
	[0.689,1.209]	[0.708,1.159]	[0.736,1.133]	[0.730,1.124]	[0.902,1.408]	[0.782,1.229]
Rhineland Palatinate	1.050	0.775	0.974	1.142	0.820	0.827
	[0.718,1.533]	[0.564,1.064]	[0.714,1.329]	[0.868,1.502]	[0.615,1.093]	[0.626,1.092]
Saarland	1.251	0.850	0.888	0.946	0.742	0.499*
	[0.612,2.560]	[0.521,1.387]	[0.577,1.368]	[0.576,1.553]	[0.440,1.250]	[0.278,0.896]
Saxony	0.726	0.744	0.789	1.141	1.187	0.709*
	[0.511,1.032]	[0.540,1.025]	[0.587,1.062]	[0.842,1.548]	[0.887,1.588]	[0.512,0.982]
Saxony-Anhalt	1.274	0.908	0.755	0.824	1.064	0.714
	[0.739,2.198]	[0.575,1.433]	[0.500,1.141]	[0.556,1.222]	[0.705,1.606]	[0.457,1.116]
Schleswig Holstein	1.079	0.866	0.644**	0.901	1.100	1.002
	[0.731,1.592]	[0.613,1.224]	[0.465,0.893]	[0.652,1.244]	[0.794,1.524]	[0.740,1.356]
Thuringia	0.956	1.000	0.860	0.893	0.694*	0.905
	[0.564,1.619]	[0.677,1.476]	[0.587,1.259]	[0.624,1.278]	[0.484,0.995]	[0.633,1.296]
Foreign country	0.400***	0.553*	0.607	1.028	0.587*	0.863
	[0.241,0.662]	[0.318,0.962]	[0.342,1.078]	[0.688,1.534]	[0.352,0.979]	[0.553,1.346]
Gender (Reference: Male)						
Female	1.033	1.196*	1.278***	0.855*	0.966	0.898
	[0.873,1.222]	[1.030,1.390]	[1.114,1.465]	[0.744,0.982]	[0.843,1.108]	[0.780,1.032]
Non-binary	0.651	2.223	0.861	0.667	0.759	1.185
	[0.303,1.397]	[0.990,4.989]	[0.495,1.499]	[0.346,1.288]	[0.301,1.914]	[0.587,2.393]
Age group (Reference: 18-30)						
31-45	1.181	1.030	1.011	0.567***	1.421***	0.718***
	[0.960,1.452]	[0.845,1.256]	[0.845,1.208]	[0.474,0.678]	[1.191,1.695]	[0.596,0.865]
46-60	1.525***	1.036	0.959	0.577***	1.658***	0.612***

	[1.237,1.882]	[0.852,1.261]	[0.802,1.148]	[0.483,0.690]	[1.388,1.981]	[0.509,0.736]
> 60	2.170***	1.104	1.268*	0.698***	1.104	1.013
	[1.744,2.701]	[0.906,1.347]	[1.057,1.521]	[0.584,0.834]	[0.918,1.326]	[0.845,1.214]
Educational attainment (Reference: Low)						
Middle	0.623*	0.630*	0.747	1.129	0.808	0.710
	[0.410,0.946]	[0.442,0.898]	[0.543,1.028]	[0.830,1.537]	[0.583,1.120]	[0.503,1.002]
High	0.512**	0.539***	0.823	1.058	0.749	0.867
	[0.340,0.771]	[0.384,0.758]	[0.603,1.122]	[0.788,1.420]	[0.549,1.022]	[0.622,1.208]
Household income (Reference: I find it very difficult to make ends meet)						
I find it quite difficult to make ends meet	0.945	1.165	1.015	1.607	1.549	1.095
	[0.555,1.611]	[0.731,1.857]	[0.645,1.599]	[0.985,2.622]	[0.937,2.560]	[0.661,1.813]
I can make ends meet	1.051	1.184	1.014	1.898**	1.481	1.308
	[0.647,1.706]	[0.783,1.789]	[0.673,1.526]	[1.226,2.938]	[0.927,2.367]	[0.821,2.082]
I can live quite comfortably on the income	1.274	1.232	1.118	2.216***	1.456	1.339
	[0.787,2.061]	[0.818,1.857]	[0.746,1.677]	[1.434,3.425]	[0.913,2.322]	[0.843,2.128]
I can live very comfortably on the income	1.076	1.246	1.026	2.068**	1.152	1.218
	[0.652,1.777]	[0.812,1.914]	[0.672,1.566]	[1.320,3.239]	[0.713,1.863]	[0.753,1.970]
does not apply (no own household income)	1.289	0.823	1.130	2.947**	1.285	2.532**
	[0.593,2.801]	[0.395,1.715]	[0.526,2.428]	[1.494,5.812]	[0.609,2.709]	[1.268,5.057]
cut1	0.198***	0.494	1.283	0.220***	1.886	0.838
	[0.0777,0.503]	[0.211,1.156]	[0.574,2.870]	[0.0999,0.486]	[0.821,4.336]	[0.357,1.966]
cut2	0.490	1.375	3.930***	0.728	4.401***	2.882*
	[0.194,1.243]	[0.590,3.207]	[1.760,8.777]	[0.331,1.601]	[1.913,10.13]	[1.228,6.764]
cut3	1.068	3.779**	10.30***	1.855	8.164***	7.385***
	[0.423,2.701]	[1.617,8.834]	[4.605,23.03]	[0.843,4.080]	[3.542,18.81]	[3.138,17.38]
cut4	3.857**	16.10***	47.61***	6.368***	18.08***	22.08***
	[1.523,9.767]	[6.853,37.81]	[21.15,107.2]	[2.890,14.03]	[7.827,41.75]	[9.305,52.39]
Mc Fadden Pseudo R^2	0.103	0.114	0.0652	0.0263	0.0216	0.0183
AIC	72644.4	89367.0	114548.2	121682.0	118313.8	101504.2

Note: The models correspond to those in Table 11, except for using the outcome variable as an ordinal scale and thus applying ordinal logistic regression. The ordinal logistic regression yields very much the same results in terms of significance and direction as the multiple linear models from Table 11. N = 6,657. Data weighted by state, age group, gender and voting intention to better represent the German population. Exponentiated coefficients; 95% confidence intervals in brackets.* $p < 0.05$, ** $p < 0.01$, *** $p < 0.001$; GAL, green/alternative/liberal; TAN, traditional/authoritarian/nationalist; AIC, Akaike Information Criterion.

Supplementary Table 13: Analysis III – Comparison between the impact on SARS-CoV-2 vaccine uptake and the impact on views towards routine pediatric immunization (MMR) (unstandardized b-coefficients from multiple linear regression + odds ratios from crosscheck with ordinal logistic regression)

Dependent variable:	# SARS-CoV-2 vaccine doses Rescaled to 0–1: 0 = 0 doses, 0.25 = 1 dose, 0.5 = 2 doses, 0.75 = 3 doses, 1 = 4 doses	How useful do you consider routine pediatric immunization, e.g. against measles/mumps/rubella (MMR)? scale: 0–1 0 = not at all meaningful, 1 = very meaningful	
	M1.5-rescaled Linear regression (b-coefficients)	M3 Linear regression (b-coefficients)	M3-crosscheck Ordinal Logit (odds ratios)
Positive attitudes towards…			
Waldorf education (scale: 0–1)	-0.0335** [-0.0581,-0.00887]	-0.0365** [-0.0586,-0.0144]	0.517*** [0.364,0.733]
Homeopathy (scale: 0–1)	-0.0938*** [-0.115,-0.0722]	-0.0903*** [-0.110,-0.0703]	0.194*** [0.142,0.266]
Mainstream medicine (scale: 0–1)	0.259*** [0.221,0.296]	0.258*** [0.223,0.293]	26.76*** [17.87,40.08]
Religious denomination (Reference: no denomination)			
Roman-Catholic	0.0183* [0.00414,0.0324]	0.0107 [-0.00239,0.0238]	1.149 [0.917,1.440]
Protestant	0.00759 [-0.00725,0.0224]	0.00244 [-0.0115,0.0164]	1.043 [0.822,1.323]
Evangelical Free Church	0.00619 [-0.0141,0.0264]	0.00469 [-0.0148,0.0242]	1.014 [0.743,1.385]
Orthodox	-0.0998 [-0.202,0.00263]	-0.0519 [-0.162,0.0588]	0.724 [0.224,2.338]
Jewish	0.0821 [-0.137,0.301]	0.0915 [-0.00325,0.186]	2.413 [0.392,14.86]
Muslim	-0.0169 [-0.103,0.0690]	-0.0421 [-0.123,0.0390]	0.487 [0.142,1.668]
Other	-0.0134 [-0.0664,0.0397]	-0.00913 [-0.0549,0.0366]	0.977 [0.550,1.736]
Left = 0 / Right = 1 (scale: 0–1)	-0.00946 [-0.0615,0.0426]	-0.0251 [-0.0699,0.0198]	0.616 [0.335,1.134]
GAL = 0 / TAN = 1 (scale: 0–1)	-0.00485 [-0.0425,0.0328]	0.0466** [0.0124,0.0808]	2.036** [1.248,3.320]
Voting Intention (Reference: CDU/CSU: Christian democrats)			
SPD (Social democrats)	0.0127 [-0.00400,0.0295]	0.00181 [-0.0143,0.0179]	1.029 [0.747,1.418]
Greens	0.00992 [-0.00887,0.0287]	0.00559 [-0.0115,0.0227]	1.077 [0.792,1.464]
FDP (Liberals)	-0.0581*** [-0.0823,-0.0339]	-0.00964 [-0.0293,0.0101]	0.783 [0.555,1.106]
Left-Party (Socialists)	-0.0401* [-0.0737,-0.00651]	-0.0131 [-0.0430,0.0169]	0.797 [0.515,1.232]
AfD (Populist radical-right)	-0.290*** [-0.322,-0.258]	-0.106*** [-0.132,-0.0798]	0.330*** [0.247,0.442]
Other (including Covid-19 protest party)	-0.179*** [-0.212,-0.147]	-0.0998*** [-0.127,-0.0730]	0.407*** [0.298,0.554]

Solidarity<br>(scale: 0–1)	0.0434*<br>[0.000921,0.0858]	0.0878***<br>[0.0479,0.128]	4.102***<br>[2.307,7.294]
Agreeableness (Big 5)<br>(scale: 0–1)	-0.0371*<br>[-0.0679,-0.00639]	-0.0360*<br>[-0.0652,-0.00673]	0.619*<br>[0.385,0.996]
Conscientiousness (Big 5)<br>(scale: 0–1)	0.00707<br>[-0.0235,0.0376]	0.00844<br>[-0.0179,0.0347]	1.525*<br>[1.000,2.325]
Extraversion (Big 5)<br>(scale: 0–1)	0.0239<br>[-0.000361,0.0481]	0.0145<br>[-0.00695,0.0360]	1.230<br>[0.860,1.760]
Neuroticism (Big 5)<br>(scale: 0–1)	0.0617***<br>[0.0347,0.0888]	0.00199<br>[-0.0224,0.0264]	0.907<br>[0.609,1.351]
Openness (Big 5)<br>(scale: 0–1)	-0.0157<br>[-0.0406,0.00930]	0.000891<br>[-0.0210,0.0228]	1.044<br>[0.728,1.497]
State (Reference: North Rhine-Westphalia)			
Baden-Wuerttemberg	-0.0370***<br>[-0.0544,-0.0197]	-0.00341<br>[-0.0199,0.0131]	0.917<br>[0.681,1.233]
Bavaria	-0.0371***<br>[-0.0553,-0.0189]	-0.0140<br>[-0.0309,0.00283]	0.734*<br>[0.567,0.950]
Berlin	-0.0485**<br>[-0.0795,-0.0174]	-0.0234<br>[-0.0528,0.00596]	0.719<br>[0.476,1.086]
Brandenburg	0.0164<br>[-0.0191,0.0519]	0.00176<br>[-0.0338,0.0373]	1.026<br>[0.592,1.779]
Bremen	0.0375<br>[-0.00691,0.0820]	0.00983<br>[-0.0450,0.0647]	1.247<br>[0.484,3.213]
Hamburg	-0.000998<br>[-0.0406,0.0386]	0.0207<br>[-0.00879,0.0502]	1.377<br>[0.754,2.515]
Hesse	-0.0252*<br>[-0.0497,-0.000787]	-0.0108<br>[-0.0330,0.0114]	0.840<br>[0.600,1.177]
Mecklenburg-Vorpommern	-0.0531*<br>[-0.106,-0.000417]	0.0154<br>[-0.0195,0.0502]	1.095<br>[0.593,2.022]
Lower Saxony	-0.00302<br>[-0.0222,0.0162]	0.00332<br>[-0.0131,0.0197]	0.945<br>[0.687,1.301]
Rhineland Palatinate	-0.0247<br>[-0.0528,0.00343]	-0.0115<br>[-0.0367,0.0138]	0.680*<br>[0.470,0.983]
Saarland	-0.0159<br>[-0.0560,0.0243]	-0.0353<br>[-0.0796,0.00902]	0.524*<br>[0.285,0.965]
Saxony	-0.0841***<br>[-0.116,-0.0525]	0.00509<br>[-0.0223,0.0325]	1.058<br>[0.681,1.642]
Saxony-Anhalt	-0.0163<br>[-0.0638,0.0313]	0.0343<br>[-0.00476,0.0734]	2.115<br>[0.977,4.580]
Schleswig Holstein	0.0122<br>[-0.0139,0.0383]	-0.000600<br>[-0.0284,0.0272]	0.906<br>[0.549,1.493]
Thuringia	-0.0276<br>[-0.0655,0.0102]	-0.00437<br>[-0.0387,0.0299]	0.842<br>[0.530,1.337]
Foreign country	-0.0645**<br>[-0.113,-0.0159]	-0.0141<br>[-0.0628,0.0345]	0.782<br>[0.420,1.457]
Gender (Reference: Male)			
Female	-0.00688<br>[-0.0200,0.00621]	0.0103<br>[-0.00102,0.0217]	1.130<br>[0.931,1.371]

Non-binary	0.00486	0.0123	0.893
	[-0.0685,0.0782]	[-0.0363,0.0609]	[0.415,1.922]
Age group (Reference: 18-30)			
31-45	-0.00655	-0.000602	0.935
	[-0.0231,0.00998]	[-0.0150,0.0138]	[0.701,1.246]
46-60	0.0140	-0.0164*	0.716*
	[-0.00294,0.0310]	[-0.0311,-0.00165]	[0.545,0.940]
> 60	0.0877***	-0.0236**	0.621***
	[0.0707,0.105]	[-0.0394,-0.00787]	[0.469,0.823]
Educational attainment (Reference: Low)			
Middle	-0.0177	0.00892	0.993
	[-0.0482,0.0128]	[-0.0217,0.0395]	[0.677,1.458]
High	-0.0271	-0.00463	0.829
	[-0.0561,0.00180]	[-0.0340,0.0247]	[0.575,1.197]
Household income (Reference: I find it very difficult to make ends meet)			
I find it quite difficult to make ends meet	0.0280	0.0208	1.110
	[-0.0240,0.0801]	[-0.0242,0.0657]	[0.696,1.771]
I can make ends meet	0.0391	0.0375	1.452
	[-0.00786,0.0860]	[-0.00361,0.0785]	[0.951,2.216]
I can live quite comfortably on the income	0.0461	0.0370	1.501
	[-0.000438,0.0927]	[-0.00365,0.0776]	[0.985,2.288]
I can live very comfortably on the income	0.0244	0.0470*	1.972**
	[-0.0233,0.0722]	[0.00564,0.0884]	[1.229,3.163]
does not apply (no own household income)	0.0129	-0.0156	0.542
	[-0.0542,0.0799]	[-0.0731,0.0418]	[0.259,1.137]
Constant	0.542***	0.693***	
	[0.459,0.625]	[0.621,0.766]	
cut1			0.0901***
			[0.0342,0.237]
cut2			0.220**
			[0.0841,0.576]
cut3			2.336
			[0.889,6.134]
Adj. R^2 / Mc Fadden Pseudo R^2	0.379	0.236	0.185
AIC			45776.2

Note: Model M1.5-rescaled corresponds to M1.5 from Supplementary Table 7 with the exception that it uses the dependent variable rescaled to the scale from 0 to 1. This is done to ease comparability to model M3 in which the dependent variable "views towards routine pediatric immunization" also measures on a scale 0–1. The b-coefficients of the two models can therefore be easily compared with each other. With respect to the three main independent variables (Waldorf, homeopathy and mainstream medicine) the models show very similar effects. Model M3-crosscheck applies ordinal logistic regression to the data from M3. With respect to direction and significance of the effects this different modeling strategy does not show any different results. N = 7,391. Data weighted by state, age group, gender and voting intention to better represent the German population. Exponentiated coefficients; 95% confidence intervals in brackets.* $p < 0.05$, ** $p < 0.01$, *** $p < 0.001$; GAL, green/alternative/liberal; TAN, traditional/authoritarian/nationalist; AIC, Akaike Information Criterion.

Supplementary Figure 1: Estimated number of SARS-CoV-2 vaccine doses by attitudes towards Waldorf education, homeopathy and mainstream medicine over age group and gender (+ 95% CI)

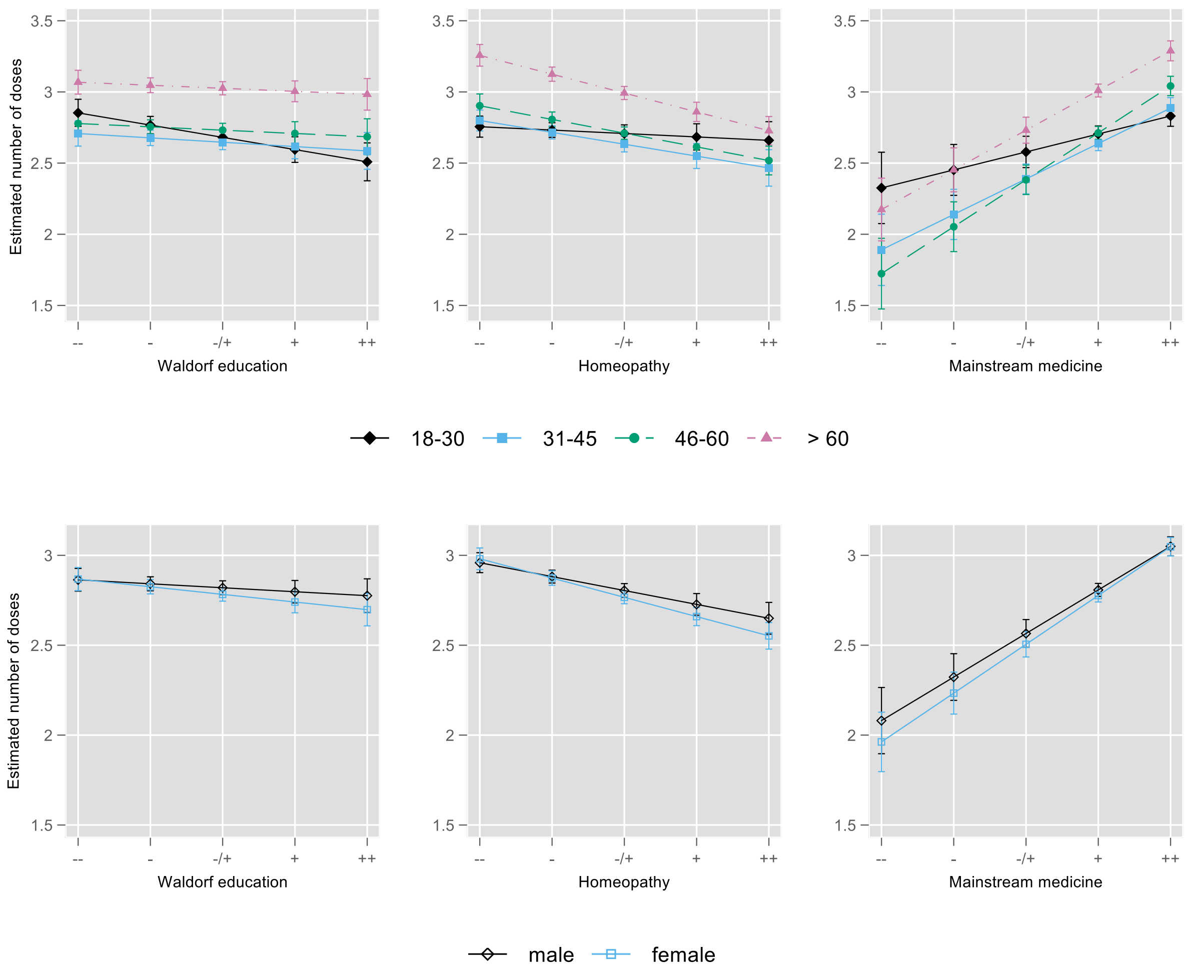

Note: N = 7,391. Data weighted by federal state, age group, gender und party affiliation to better represent the German population. The graphs in the top row are based on model "Interaction 1: Attitudes x Age", those in the lower row on model "Interaction 2: Attitudes x gender" both from Supplementary Table 10. The figure presents the predicted number of SARS-CoV-2 immunization doses by the three attitudes variables and age as well as gender. While a positive attitude towards mainstream medicine is always associated with a higher number of doses, this is not so much the case for the youngest age group (18-30 years). For the attitude towards homeopathy this picture is reversed. Here a more positive attitude is associated with a lower estimated number of immunization doses particularly for the older generations, while attitudes towards homeopathy play no major role in VacHes for those 18-30 years old. For the attitudes towards Waldorf education the picture is again different. Here, for the older age groups (> 30) the model estimates no major difference in the number of doses for those in favor of, or those opposed to, this form of schooling. Only for the youngest age group (18-30) a positive attitude towards Waldorf schools is negatively related to the number of doses. Regarding gender, we found no significant differences in the effects of the three attitude variables for females and males on the number of doses.

Supplementary Figure 2: Average Marginal Effect of attitudes towards Waldorf schools, homeopathy and mainstream medicine on the number of SARS-CoV-2 vaccine doses by state (+ 95% CI)

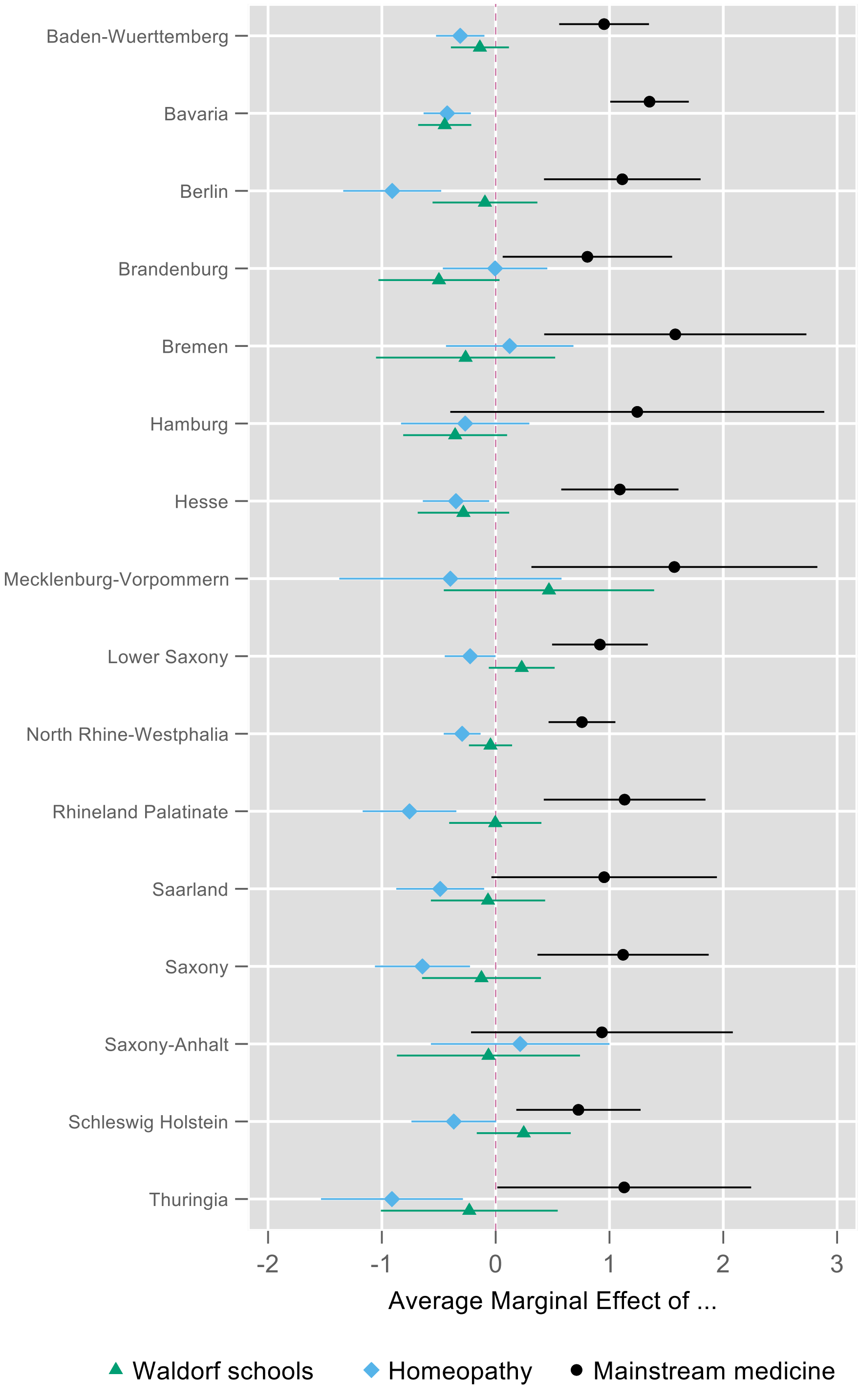

Note: N = 7,391. Data weighted by state, age group, gender und party affiliation to better represent the German population. The graph is based on model "Interaction 3: Attitudes x state" from Supplementary Table 10. The figure presents Average Marginal Effects (AMEs) for the three attitudes variables by state. In nearly all states, the attitudes towards mainstream medicine are associated with a significantly higher number of doses ($p < 0.05$). Only in some states where few participants took part in the survey, such as Hamburg or Saxony-Anhalt, the effects are not significantly different from zero. Yet, they are always at least by trend positive. With regard to the other two variables we find that a more positive attitude towards homeopathy has a significantly (p <

0.05) negative effect in 10 out of the 16 states (e.g. in Baden-Wuerttemberg, Bavaria, Berlin, Rhineland-Palatinate, Saxony, Thuringia) and that the only significant ($p < 0.05$) effect with regard to Waldorf education can be found in Bavaria (Brandenburg barely misses $p < 0.05$). Only in this southern state is a more positive attitude towards Waldorf education associated with a lower number of vaccine doses. Yet, the trend is also quite clear for this variable. In 13 out of 16 states the AME is negative which shows that if the attitude towards Waldorf education has any impact on VacHes, it is a negative one in most states.

Supplementary Figure 3: Distribution of main demographic variables in the sample compared to reality

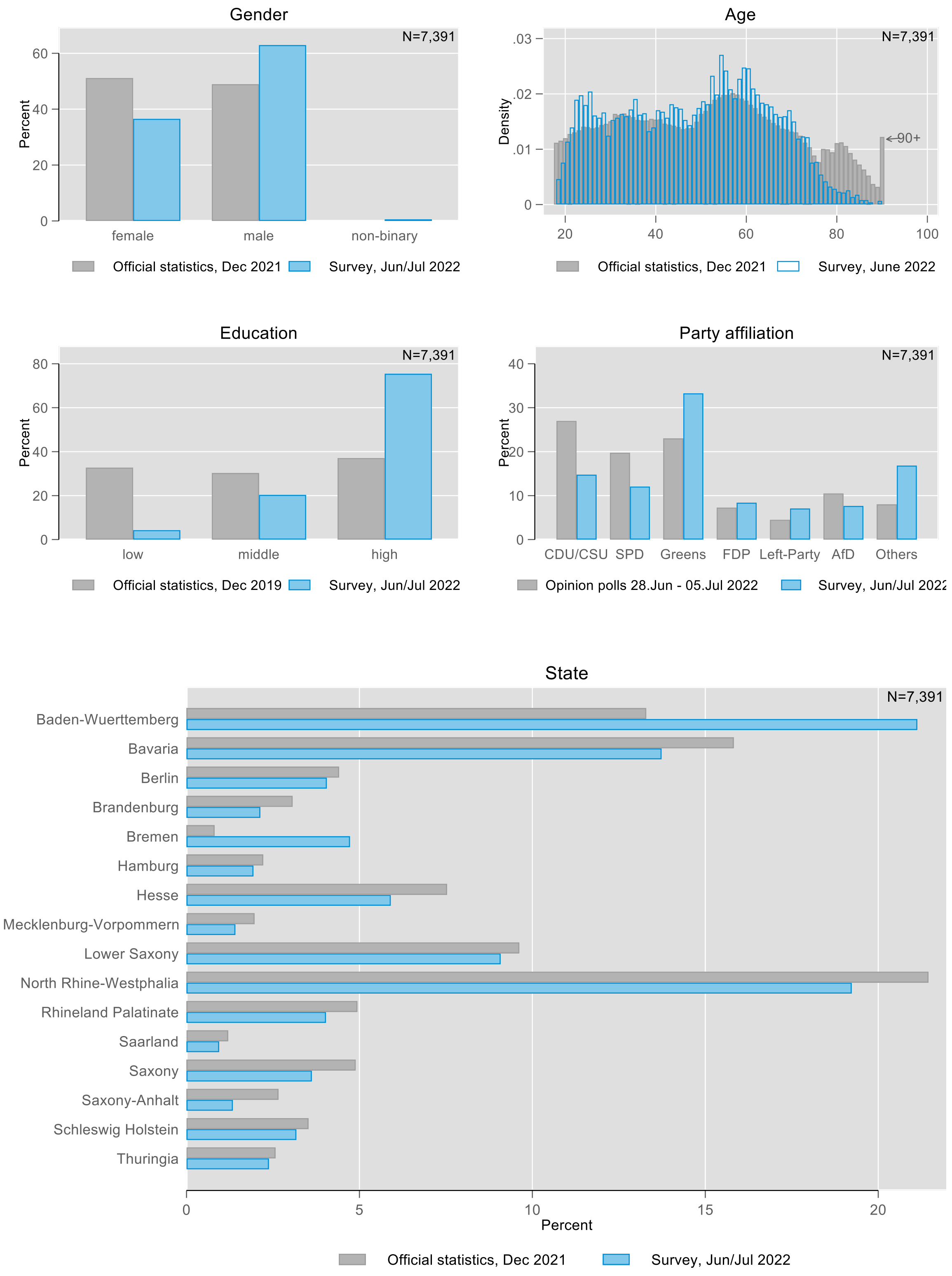

Note: Official statistics from the Federal Statistical Office of Germany, Destatis (https://www-genesis.destatis.de/genesis/online). Opinion poll data is the mean of the Forschungsgruppe Wahlen, FORSA, Infratest Dimap, and YouGov polls (source: https://www.wahlrecht.de/umfragen/).

Supplementary Figure 4: Predicted number of SARS-CoV-2 vaccine doses (+ 95% CI): Crosscheck multiple linear regression vs. Poisson regression

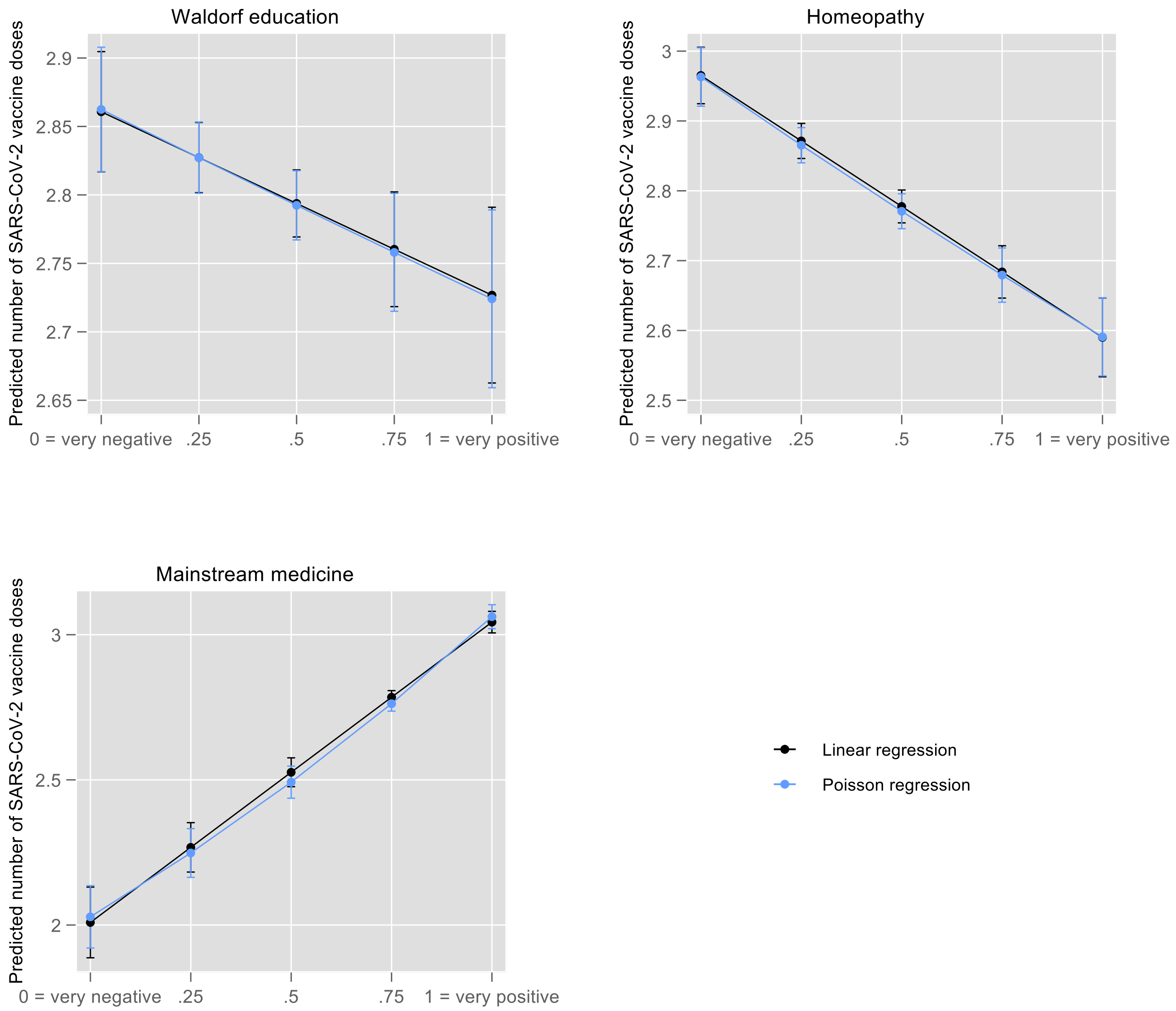

Note: The three graphs present the predicted number of SARS-CoV-2 doses for all values of the three main independent variables (attitudes towards Waldorf education, homeopathy and mainstream medicine) – for this estimation all other variables are set to their respective mean. The black lines are based on the main model M1.5 from Analysis I (Supplementary Table 7), the blue lines are based a Poisson model with an identical specification of included variables. Since the differences between the two lines are marginal in all three plots, the use of a multiple linear regression model with its easier to interpret b-coefficients is warranted. N = 7,391. Data weighted by state, age group, gender und party affiliation to better represent the German population.

Literature

1. Weidmann, N. *Esoteric Beliefs and Opposition to Corona Restrictions*. https://kops.uni-konstanz.de/entities/publication/b1453a44-a697-4386-ae4d-68241e592e2a (2022).
2. Bartelme, R. R. Anthroposophic Medicine: A Short Monograph and Narrative Review—Foundations, Essential Characteristics, Scientific Basis, Safety, Effectiveness and Misconceptions. *Glob Adv Health Med* **9**, 216495612097363 (2020).
3. Salchegger, S., Wallner-Paschon, C. & Bertsch, C. Explaining Waldorf students' high motivation but moderate achievement in science: is inquiry-based science education the key? *Large-scale Assess Educ* **9**, 14 (2021).
4. Büssing, A., Ostermann, T., Majorek, M. & Matthiessen, P. F. Eurythmy Therapy in clinical studies: a systematic literature review. *BMC Complement Altern Med* **8**, 8 (2008).
5. Hague Circle. *Waldorf World List*. https://www.freunde-waldorf.de/fileadmin/user_upload/images/Waldorf_World_List/Waldorf_World_List.pdf (2022).
6. Destatis. *Freie Waldorfschulen: 2018 so Viele Kinder Wie Nie Zuvor Eingeschult*. https://www.destatis.de/DE/Presse/Pressemitteilungen/2019/09/PD19_364_211.html (2019).
7. Sobo, E. J. Social Cultivation of Vaccine Refusal and Delay among Waldorf (Steiner) School Parents: Social Cultivation of Vaccine Refusal. *MEDICAL ANTHROPOLOGY QUARTERLY* **29**, 381–399 (2015).
8. Pfaff, G., Leher, A., Fechler, A. & Ouédraogo, N. Immunization coverage among children in Waldorf kindergartens, South West Germany 2015-2016. *European Journal of Public Health* **27**, (2017).
9. Arenz, S. *et al.* Der Masernausbruch in Coburg: Was lässt sich daraus lernen? *Deutsches Ärzteblatt* **100**, A3245–A3249 (2003).
10. Bätzing-Feigenbaum, J. *et al.* Spotlight on measles 2010: Preliminary report of an ongoing measles outbreak in a subpopulation with low vaccination coverage in Berlin, Germany, January-March 2010. *Eurosurveillance* **15**, (2010).
11. Roggendorf, H., Mankertz, A., Kundt, R. & Roggendorf, M. Spotlight on measles 2010: Measles outbreak in a mainly unvaccinated community in Essen, Germany, March – June 2010. *Eurosurveillance* **15**, (2010).
12. Yasgur, J. Book Review of The Healing Power of Planetary Metals in Anthroposophic and Homeopathic Medicine by Henning M. Schramm. *Homœopathic Links* **29**, 159–159 (2016).
13. Kienle, G. S. *et al.* Anthroposophic Medicine: An Integrative Medical System Originating in Europe. *Glob Adv Health Med* **2**, 20–31 (2013).
14. Goldner, C. Offenbart in 'mystischer Schau'. *Süddeutsche Zeitung* (2010).
15. National Center for Complementary and Integrative Health. *Homeopathy: What You Need To Know*. https://www.nccih.nih.gov/health/homeopathy (2021).
16. Ernst, E. Homeopathy: what does the "best" evidence tell us? *Medical Journal of Australia* **192**, 458–460 (2010).

17. Shang, A. *et al.* Are the clinical effects of homoeopathy placebo effects? Comparative study of placebo-controlled trials of homoeopathy and allopathy. *The Lancet* **366**, 726–732 (2005).

18. House of Commons, Science and Technology Committee. *Fourth Report. Evidence Check 2: Homeopathy*. https://publications.parliament.uk/pa/cm200910/cmselect/cmsctech/45/4502.htm (2010).

19. National Health and Medical Research Council. *NHMRC Information Paper: Evidence on the Effectiveness of Homeopathy for Treating*. https://www.nhmrc.gov.au/sites/default/files/images/nhmrc-information-paper-effectiveness-of-homeopathy.pdf (2015).

20. European Academies' Science Advisory Council. *Homeopathic Products and Practices: Assessing the Evidence and Ensuring Consistency in Regulating Medical Claims in the EU*. https://easac.eu/fileadmin/PDF_s/reports_statements/EASAC_Homepathy_statement_web_final.pdf (2017).

21. Hornsey, M. J., Lobera, J. & Díaz-Catalán, C. Vaccine hesitancy is strongly associated with distrust of conventional medicine, and only weakly associated with trust in alternative medicine. *Social Science & Medicine* **255**, 113019 (2020).

22. Choi, Y. & Fox, A. M. Mistrust in public health institutions is a stronger predictor of vaccine hesitancy and uptake than Trust in Trump. *Social Science & Medicine* **314**, 115440 (2022).

23. Jennings, W. *et al.* Trust and vaccine hesitancy during the COVID-19 pandemic: A cross-national analysis. *Vaccine: X* **14**, 100299 (2023).

24. Ernst, E. Anthroposophy: A Risk Factor for Noncompliance With Measles Immunization. *Pediatric Infectious Disease Journal* **30**, 187–189 (2011).

25. Mayer, T. *Covid Vaccines from a Spiritual Perspective: Consequences for the Soul and Spirit and for Life after Death*. (Clairview Books, 2022).

26. Schoen-Angerer, T., Breitkreuz, T., Girke, M. & Soldner, G. Unwissenschaftlich und manipulativ. *Das Goetheanum* (2022).

27. IfD Allensbach. *Homöopathie: Nutzung Und Wertschätzung in Der Bevölkerung*. https://www.ifd-allensbach.de/fileadmin/IfD/sonstige_pdfs/2023_03_15_Pressemitteilung_Homoeopathie.pdf (2023).

28. Williams, J. T. B., Rice, J. D. & O'Leary, S. T. Associations between religion, religiosity, and parental vaccine hesitancy. *Vaccine: X* **9**, 100121 (2021).

29. Troiano, G. & Nardi, A. Vaccine hesitancy in the era of COVID-19. *Public Health* **194**, 245–251 (2021).

30. Garcia, L. L. & Yap, J. F. C. The role of religiosity in COVID-19 vaccine hesitancy. *Journal of Public Health* **43**, e529–e530 (2021).

31. Marti, M., de Cola, M., MacDonald, N. E., Dumolard, L. & Duclos, P. Assessments of global drivers of vaccine hesitancy in 2014—Looking beyond safety concerns. *PLoS ONE* **12**, e0172310 (2017).

32. Martinez, E. Z. *et al.* Brazilian Adults' Attitudes and Practices Regarding the Mandatory COVID-19 Vaccination and Their Hesitancy towards Childhood Vaccination. *Vaccines* **10**, 1853 (2022).
33. Nagar, S. & Ashaye, T. A Shot of Faith—Analyzing Vaccine Hesitancy in Certain Religious Communities in the United States. *Am J Health Promot* **36**, 765–767 (2022).
34. Corcoran, K. E., Scheitle, C. P. & DiGregorio, B. D. Christian nationalism and COVID-19 vaccine hesitancy and uptake. *Vaccine* **39**, 6614–6621 (2021).
35. Frerk, C. *Religionszugehörigkeiten 2020*. https://fowid.de/meldung/religionszugehoerigkeiten-2020 (2021).
36. Hussain, A., Ali, S., Ahmed, M. & Hussain, S. The Anti-vaccination Movement: A Regression in Modern Medicine. *Cureus* (2018) doi:10.7759/cureus.2919.
37. Eggertson, L. Lancet retracts 12-year-old article linking autism to MMR vaccines. *Canadian Medical Association Journal* **182**, E199–E200 (2010).
38. He, K., Mack, W. J., Neely, M., Lewis, L. & Anand, V. Parental Perspectives on Immunizations: Impact of the COVID-19 Pandemic on Childhood Vaccine Hesitancy. *J Community Health* **47**, 39–52 (2022).
39. Opel, D. J. *et al.* Parent Attitudes Towards Childhood Vaccines After the Onset of SARS-CoV-2 in the United States. *Academic Pediatrics* **22**, 1407–1413 (2022).
40. Alfieri, N. L. *et al.* Parental COVID-19 vaccine hesitancy for children: vulnerability in an urban hotspot. *BMC Public Health* **21**, 1662 (2021).
41. Kara, A. *et al.* Alteration in vaccination rates and an evaluation of physicians' perceptions of the possible impact of the SARS-CoV-2 pandemic on childhood vaccinations in Ankara, Turkey. *Human Vaccines & Immunotherapeutics* **17**, 3457–3462 (2021).
42. Salazar, T. L., Pollard, D. L., Pina-Thomas, D. M. & Benton, M. J. Parental vaccine hesitancy and concerns regarding the COVID-19 virus. *Journal of Pediatric Nursing* **65**, 10–15 (2022).
43. Durmaz, N., Suman, M., Ersoy, M. & Örün, E. Parents' Attitudes toward Childhood Vaccines and COVID-19 Vaccines in a Turkish Pediatric Outpatient Population. *Vaccines* **10**, 1958 (2022).
44. LaVail, K. H. & Kennedy, A. M. The Role of Attitudes About Vaccine Safety, Efficacy, and Value in Explaining Parents' Reported Vaccination Behavior. *Health Educ Behav* **40**, 544–551 (2013).
45. Greyson, D. & Bettinger, J. A. How do mothers' vaccine attitudes change over time? *SSM - Qualitative Research in Health* **2**, 100060 (2022).
46. Sorell, T. & Butler, J. The Politics of Covid Vaccine Hesitancy and Opposition. *Political Quarterly* **93**, 347–351 (2022).
47. Albrecht, D. Vaccination, politics and COVID-19 impacts. *BMC Public Health* **22**, 96 (2022).

48. Jäckle, S. & Timmis, J. K. Left–Right-Position, party affiliation and regional differences explain low COVID-19 vaccination rates in Germany. *Microbial Biotechnology* **16**, 662–677 (2023).

49. Lewandowsky, M., Leonhardt, C. & Blätte, A. Germany - The Alternative for Germany in the Covid-19 Pandemic. in *Populists and the Pandemic* 237–249 (Routledge, London, 2022). doi:10.4324/9781003197614-21.

50. John, O. P., Naumann, L. P. & Soto, C. J. Paradigm shift to the integrative Big Five trait taxonomy. in *Handbook of Personality Theory and Research* (eds. John, O. P., Robins, R. W. & Pervin, L. A.) 114–156 (Guilford Publications, 2008).

51. Gerber, A. S., Huber, G. A., Doherty, D., Dowling, C. M. & Panagopoulos, C. Big Five Personality Traits and Responses to Persuasive Appeals: Results from Voter Turnout Experiments. *Polit Behav* **35**, 687–728 (2013).

52. Huber, B., Goyanes, M. & Gil De Zúñiga, H. Linking Extraversion to Collective and Individual Forms of Political Participation: The Mediating Role of Political Discussion. *Social Science Quarterly* **102**, 1289–1310 (2021).

53. Howard, M. C. The good, the bad, and the neutral: Vaccine hesitancy mediates the relations of Psychological Capital, the Dark Triad, and the Big Five with vaccination willingness and behaviors. *Personality and Individual Differences* **190**, 111523 (2022).

54. Reagu, S., Jones, R. M. & Alabdulla, M. COVID-19 Vaccine Hesitancy and Personality Traits; Results from a Large National Cross-Sectional Survey in Qatar. *Vaccines* **11**, 189 (2023).

55. Nanteer-Oteng, E., Kretchy, I. A., Nanteer, D. O., Kretchy, J.-P. & Osafo, J. Hesitancy towards COVID-19 vaccination: The role of personality traits, anti-vaccine attitudes and illness perception. *PLOS Glob Public Health* **2**, e0001435 (2022).

56. Seither, R., Laury, J., Mugerwa-Kasujja, A., Knighton, C. L. & Black, C. L. Vaccination Coverage with Selected Vaccines and Exemption Rates Among Children in Kindergarten — United States, 2020–21 School Year. *MMWR Morb. Mortal. Wkly. Rep.* **71**, 561–568 (2022).

57. Brennan, J. M. *et al.* Trends in Personal Belief Exemption Rates Among Alternative Private Schools: Waldorf, Montessori, and Holistic Kindergartens in California, 2000–2014. *Am J Public Health* **107**, 108–112 (2017).

58. Herzig van Wees, S., Abunnaja, K. & Mounier-Jack, S. *Understanding and Explaining the Link between Anthroposophy and Vaccine Hesitancy: A Systematic Review*. https://www.researchsquare.com/article/rs-2208907/v1 (2022) doi:10.21203/rs.3.rs-2208907/v1.

59. Cheung, F. TCM: Made in China. *Nature* **480**, S82–S83 (2011).

60. Elahee, S. F., Mao, H., Zohra, F., Faruque, S. M. B. & Shen, X. Homeopathic Medicine Versus Traditional Chinese Medicine: An Analytical Overview. *Chinese Medicine and Culture* **3**, 1–9 (2020).

61. Eigenschink, M., Dearing, L., Dablander, T. E., Maier, J. & Sitte, H. H. A critical examination of the main premises of Traditional Chinese Medicine. *Wien Klin Wochenschr* **132**, 260–273 (2020).

62. Guo, Y. *et al.* Acceptability of Traditional Chinese Medicine in Chinese People Based on 10-Year's Real World Study With Mutiple Big Data Mining. *Front. Public Health* **9**, 811730 (2022).

63. Luo, W. & Song, S. Perceived Benefits and Barriers to Chinese COVID-19 Vaccine Uptake Among Young Adults in China. *Front. Public Health* **10**, 825874 (2022).

64. Matysiak-Klose, D. *et al.* STIKO-Empfehlung zur Optimierung der Grundimmunisierung nach einmaliger Gabe von COVID-19 Vaccine Janssen und zur Auffrischung mit einer dritten Impfstoffdosis und die dazugehörige wissenschaftliche Begründung. *Epidemiologisches Bulletin* **2022**, 32–41 (2022).

65. Rammstedt, B., Kemper, C. J., Klein, M. C., Beierlein, C. & Kovaleva, A. *Big Five Inventory (BFI-10)*. https://zis.gesis.org/DoiId/zis76 (2014).
